# Natural Products for the Prevention and Control of the COVID-19 Pandemic: Sustainable Bioresources

**DOI:** 10.3389/fphar.2021.758159

**Published:** 2021-12-01

**Authors:** Rajeev K. Singla, Xuefei He, Hitesh Chopra, Christos Tsagkaris, Li Shen, Mohammad Amjad Kamal, Bairong Shen

**Affiliations:** ^1^ Institutes for Systems Genetics, Frontiers Science Center for Disease-Related Molecular Network, West China Hospital, Sichuan University, Chengdu, China; ^2^ iGlobal Research and Publishing Foundation, New Delhi, India; ^3^ Chitkara College of Pharmacy, Chitkara University, Rajpura, India; ^4^ Faculty of Medicine, University of Crete, Heraklion, Greece; ^5^ West China School of Nursing/Institutes for Systems Genetics, Frontiers Science Center for Disease-related Molecular Network, West China Hospital, Sichuan University, Chengdu, China; ^6^ King Fahd Medical Research Center, King Abdulaziz University, Jeddah, Saudi Arabia; ^7^ Enzymoics; Novel Global Community Educational Foundation, Hebersham, NSW, Australia

**Keywords:** SARS-CoV-2, complementary medicine, secondary metabolites, polyherbal formulation, intestinal microbiota, pro-inflammatory markers

## Abstract

**Background:** The world has been unprecedentedly hit by a global pandemic which broke the record of deadly pandemics that faced humanity ever since its existence. Even kids are well-versed in the terminologies and basics of the SARS-CoV-2 virus and COVID-19 now. The vaccination program has been successfully launched in various countries, given that the huge global population of concern is still far behind to be vaccinated. Furthermore, the scarcity of any potential drug against the COVID-19-causing virus forces scientists and clinicians to search for alternative and complementary medicines on a war-footing basis.

**Aims and Objectives:** The present review aims to cover and analyze the etiology and epidemiology of COVID-19, the role of intestinal microbiota and pro-inflammatory markers, and most importantly, the natural products to combat this deadly SARS-CoV-2 virus.

**Methods:** A primary literature search was conducted through PubMed and Google Scholar using relevant keywords. Natural products were searched from January 2020 to November 2020. No timeline limit has been imposed on the search for the biological sources of those phytochemicals. Interactive mapping has been done to analyze the multi-modal and multi-target sources.

**Results and Discussion:** The intestinal microbiota and the pro-inflammatory markers that can serve the prognosis, diagnosis, and treatment of COVID-19 were discussed. The literature search resulted in yielding 70 phytochemicals and ten polyherbal formulations which were scientifically analyzed against the SARS-CoV-2 virus and its targets and found significant. Retrospective analyses led to provide information about 165 biological sources that can also be screened if not done earlier.

**Conclusion:** The interactive analysis mapping of biological sources with phytochemicals and targets as well as that of phytochemical class with phytochemicals and COVID-19 targets yielded insights into the multitarget and multimodal evidence-based complementary medicines.

## 1 Introduction

A virus can be defined as a dead or alive particle that completely relies on the host to thrive and replicate further (Fermin, 2018). Plants, animals and humans can serve as hosts. In general, viruses can be classified on the basis of their replication and growth mechanism (Lodish et al., 2000). The most common virus is influenza (flu) which generally causes chills, headaches, muscle pain, and fever and can survive for about 18–20 days in humans (Eccles, 2005). A virus may be transmitted from host to host (E.g. Coronavirus) (Riou and Althaus, 2020). Coronaviruses have existed for a long time as microbial flora or pathogens in bats, camels, and cats (Singla et al., 2020). The first documented infectious outbreak and public health emergency associated with coronaviruses was identified in 2003 in the form of severe acute respiratory syndrome (SARS) (Yang Y. et al., 2020).

Currently, the world is experiencing the fifth pandemic after the 1918 flu (Liu YC. et al., 2020). The cause of the present pandemic is the novel coronavirus disease (COVID-19), a communicable viral infection caused by the severe acute respiratory syndrome coronavirus-2 (SARS-CoV-2) (Zheng, 2020). At the end of 2019, SARS-CoV-2 was first identified in Wuhan city in the People’s Republic of China (PRC) and then spread globally as a pandemic. The virus may get transmitted from human to human through respiratory droplets produced in high quantities during coughing, sneezing, shouting, singing and even talking. The virus can survive on various surfaces from a few seconds to many days. For example, it may remain on plastic for up to two to 3 days, stainless steel for up to two or 3 days, cardboard for up to 1 day, and copper for up to 4 hours (van Doremalen et al., 2020). It has been found that the infection is associated with worse outcomes in individuals with comorbidities and/or immune compromise (Wei J. et al., 2020). The spread of the infection and the lack of etiological treatment has necessitated country and region-wide restrictive measures including travel bans, lockdowns and social distancing practices. These measures in combination with personal protective equipment and personal hygiene have commendably lowered the spread of the virus in expectation of vaccines and etiological treatments. However, financial, professional and social activity have been negatively affected, making the discovery of effective treatment regimens a dire need. (Atalan, 2020).

## 2 Methodology

The authors performed a literature search with keywords, related to different phytochemical classes, natural products, microbiota, pro-inflammatory markers, SARS, coronavirus, and COVID-19 related terminologies, literature was collected from PubMed and Google Scholar search engines. Natural products were searched from January 2020 to November 2020. No time limit was applied to the search of studies related to the etiology and epidemiology of COVID-19, intestinal microbiota and pro-inflammatory markers, biological products, their origin and mechanisms of action. Relevant clinical studies focusing on natural products have been searched without a time limit as well. Articles published in languages other than English, review articles, short communications, articles published in non-peer—reviewed sources, including those without PubMed Identification (PMID) or Digital Object Identifier (DOI) were excluded to ensure the credibility and reproducibility of the study.

## 3 COVID-19: Etiology and Epidemiology

### 3.1 Etiology

Coronaviruses are positive-stranded RNA viruses with a crown-like appearance under an electron microscope due to the presence of spike glycoproteins (S protein) (Yan et al., 2020). The subfamily of *orthocoronavirinae* in the *Coronaviridae* family is subdivided into four CoVs genera, i.e., alphacoronavirus (alphaCoV), betacoronavirus (betaCoV), deltacoronavirus (deltaCoV), and gammacoronavirus (gammaCoV) (Chan et al., 2013). Genomic evaluation showed that bats and rodents are the gene sources of alphaCoVs and betaCoVs, respectively, while the avian species are sources of deltaCoVs and gammaCoVs (Su et al., 2016). The virus can cause respiratory, enteric, hepatic, and neurological diseases (Kahn and McIntosh, 2005). HCoV-OC43 and HCoV-HKU1 (lineage A betaCoVs); HCoV-229E, and HCoV-NL63 (alphaCoVs) have been identified as the human CoVs. Most of them are associated with mild immune responses such as common colds and upper respiratory tract infections, especially in immunocompromised people. However, SARS-CoV, SARS-CoV-2, and MERS-CoV (lineage B and C betaCoVs, respectively) are epidemic causing variables associated with adverse outcomes in subjects of all ages. Exposing the virus to heat treatment at a temperature above 75°C for 3 min results in its inactivation (Abraham et al., 2020; Raeiszadeh and Adeli, 2020). Exposure to higher temperatures causes a decrease in the replication rate. It is also inactivated by lipid solubilizing solvents, such as ether, ethanol, chlorine-containing disinfectants, peroxyacetic acid, and etc (Jing et al., 2020).

SARS-CoV-2 has a single-stranded RNA envelope. For its characterization, a metagenomic next-generation sequencing approach was applied, which is 29881 bp in length and encodes 9,860 amino acids (Chen L. et al., 2020). Two types of proteins are expressed as structural and non-structural using gene fragmentation (Mousavizadeh and Ghasemi, 2020). The S, E, M, and N gene codes are for structural proteins, whereas non-structural 3-chymotrypsin-like protease, papain-like protease, and RNA-dependent RNA polymerase are encoded by the ORF region. The S glycoproteins are present in the surface of SARS-CoV-2 that binds to the ACE2 host cell receptor and potentiates the penetration of the virus to the cell. As the S protein binds to the receptor, the TM protease Serine 2, positioned at the host cell membrane, helps in entering into the cell and activating the S protein. As the virus gets cell entry, the viral RNA is released in the process of RNA replication. Then, transcription takes place through protein cleavage and the assembly of the replicase-transcriptase complex (Chen L. et al., 2020). Structural proteins are synthesized, assembled, and packaged in the host cell and viral particles are released further.

### 3.2 Transmission

**FIGURE 1:**
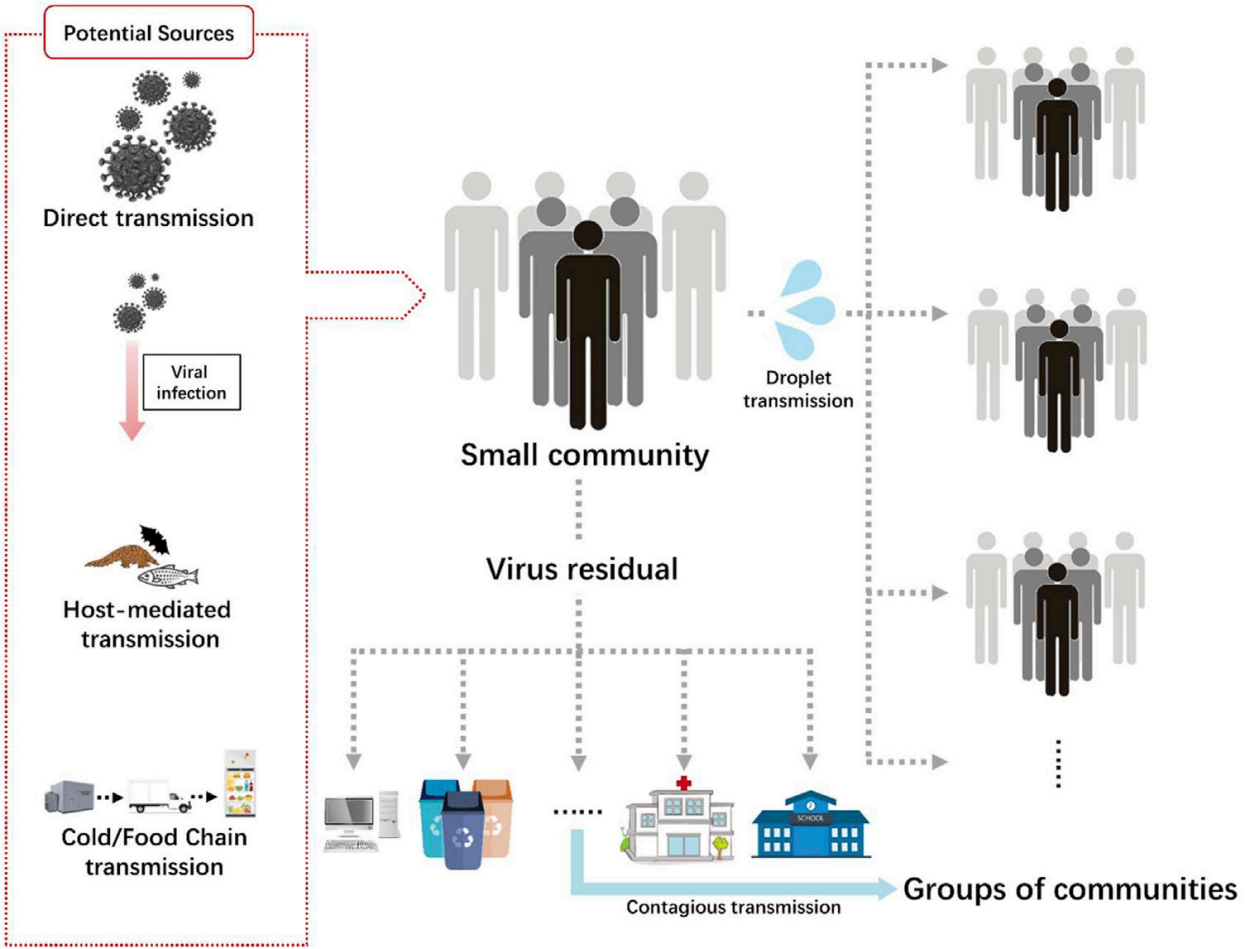
Transmission routes of SARS-CoV-2.

The transmission routes of SARS-CoV-2 are shown in Figure 1. The first case was identified in a seafood market in Wuhan, China; however, other cases were not linked with it. Human to human transmission occurred later and people acted as hosts and carriers of the virus (Riou and Althaus, 2020). The presentation of the infection included fever, dry cough, tiredness, arthralgia, anosmia (loss of smell) and loss of taste. Symptomatic individuals were isolated and kept in quarantine for a certain period of time. Viral transmission was associated with respiratory droplets from coughing and sneezing (Dhand and Li, 2020). Asymptomatic individuals can also transmit the infection. Given that they are not quarantined, they may spread the infection up to 80% more than symptomatic individuals, who are diagnosed and isolated on time (Ford et al., 2020). There is some evidence that the transmission of the virus is more prevalent in intensive care units (ICUs), compared with general wards, perhaps due to the abundance of devices producing aerosols. This applies to COVID-19 patients hospitalized in such departments among non—COVID-19 patients. Such a comparison is not applicable to COVID-19 wards, where all the patients are infected. Additionally, the virus can be found on floors, computer mice, trash bins, and door handles and people can be infected through hand contact with the contaminated surfaces (Guo et al., 2020). Based on data from China CDC and local CDCs, it has been found that the virus can remain incubated for about three to 7 days and the time from infection to symptoms takes 12.5 days (Li Q. et al., 2020). The data showed that the virus gets doubly replicated every 7 days (T.K and G, 2020).

With a particle size lower than 100 μm, airborne transmission is primarily suspected of transmitting SARS-CoV-2 (Jayaweera et al., 2020). Aerosols may originate from dental activities and various medical surgeries and procedures, such as endotracheal intubation, bronchoscopy, open suctioning, nebulized treatment administration, manual ventilation before intubation, turning the patient into the prone position, disconnecting the patient from the ventilator, non-invasive positive-pressure ventilation, tracheostomy, and cardiopulmonary resuscitation. Furthermore, aerosols may be produced by a droplet oozed during a normal conversation or an infected subject coughing and sneezing (Tran et al., 2012). These findings have also been corroborated by many studies. In a study by Lai et al., many healthcare workers were infected while they were treating the patients in Tongji Hospital in Wuhan, China (Lai X. et al., 2020). The study shows that 9,684 healthcare workers were undertaken and 110 of them had COVID-19 with an infection rate of 1.1%. A major infection rate of about 71.8% was found in nurses (70 nurses), with a median age of 36.5 years. However, no surfaces were tested positive for COVID. The commonly observed symptoms were fever, myalgia or fatigue, cough, sore throat, and muscle ache. For taking precautions, the World Health Organization (WHO) recommended a set of protocols to be followed.

Another mode of SARS-CoV-2 transmission is self-inoculation. It may occur through poor hand hygiene or poorly following the disease-controlling etiquettes (Przekwas and Chen, 2020). Viral transmission has been increased due to frequently touching contaminated fomites.

Besides airborne transmission, the fecal route has also a discernible effect on the transmission of the virus (Heller et al., 2020). A study conducted in China showed that out of 1,070 specimens collected from 205 COVID patients from three different hospitals, the virus in 29% of the positive COVID cases was transmitted through fecal route after they observed live infectious agents in the patients’ stools (Wang W. et al., 2020). Xing *et al.*, examined three patients for the continually shredding of the virus through stools, even after the nasopharynx samples showed negative results (Xing et al., 2020). Consequently, there is a strong need for the inclusion of feces or anal swab tests before discharging patients after recovering from COVID-19.

### 3.3 Epidemiology

Earlier studies showed that about 66% of COVID cases in China were due to the seafood market in which various living wild animals, including bats, marmots, and poultry, were on sale (Chen N. et al., 2020; Huang et al., 2020). This has been linked to the sudden outbreak of COVID in Wuhan city. The WHO investigation reports showed that the Huanan seafood market samples were tested positive for COVID, but linking it to specific animals was not established.

Until October 11, 2021, a total of 238,664,271 positive cases and 4,867,551 deaths have been reported around the world according to Worldometer. info (Worldometer, 2020). 215,862,052 cases out of them have recovered, with an average recovery rate of 90.45%. About 100,751,486 positive cases (42.21%) of the total cases have been reported in the United States, India, and Brazil only. Apart from these three countries, the other top ten countries included UK, Russia, Turkey, France, Iran, Argentina, and Spain. All these countries contributed to more than 60% of the total reported cases. While Seychelles topped in total cases per million people, with 218,297counts, Peru topped in deaths per million people in the list of around 220 countries.

## 4 Intestinal Microbiota and Pro-inflammatory Markers in COVID-19: Prognosis, Diagnosis, and Treatment

### 4.1 Intestinal Microbiota and Pro-inflammatory Markers

The human gastrointestinal tract hosts around 1,014 resident microorganisms such as bacteria, archaea, viruses, and fungi (Gill et al., 2006). The prevailing gut bacteria in healthy individuals include the phyla of *Actinobacteria, Firmicutes, Proteobacteria, and Bacteroidetes*. The bacterial families *Bacteroidaceae, Prevotellaceae, Rikenellaceae, Lachnospiraceae*, and *Ruminococcaceae* reside in the colon in large numbers (van der Lelie et al., 2020). The gut microbiota populations consist of at least one trillion microorganisms and weigh up to 3 kg (Rooks and Garrett, 2016; Nagpal et al., 2018). The microbiota’s genetic material inherently regulates their population dynamics and the expression of a wide range of biomolecules.

During pathogen infection, the gut microbiota will act as competitors in the antivirus combat. Meanwhile, the myeloid cells will be activated and cytokines such as IL-6, IL-1, and TNF will be released. Then, it will be followed by an increased expression of cytokine-related receptors (e.g., IFN-α/β receptor). Cytokine activated genes (CAGs) will be transcribed and then proteins with antiviral functions will be coded. Combined with Th17 cells, released cytokines will induce inflammation through NF-κβ or JAK-STAT signaling pathway. The gut microbiota also play a role in reducing inflammation in case of hypersensitivity.

**FIGURE 2:**
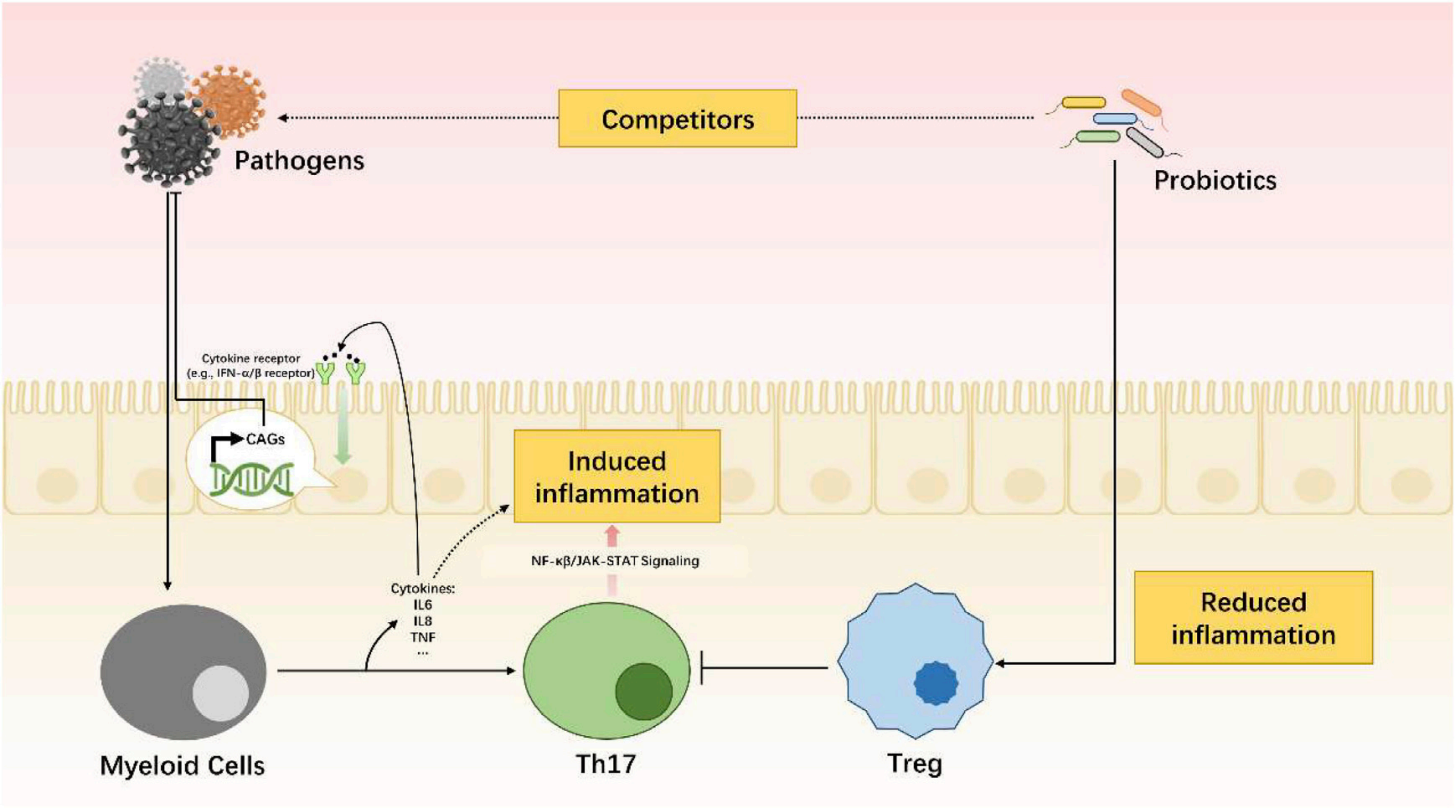
Dynamic balance of immune system mediated by gut microbiota.

Constant crosstalk between the microbiome and the human body provides them with habitat and nourishment. In return, the microbiome contributes to the regulation of the host’s physiological functions in terms of digestion and immunity (Figure 2). Digestion is co-facilitated by substances produced by microorganisms (Singh et al., 2017; Anand and Mande, 2018). At the same time, microorganisms serve as competitors against intruding pathogens. The gastrointestinal immune tissue maintains a balance between Th17 lymphocytes and T-regulatory cells (Tregs) to supervise the microorganisms’ population growth. This balanced coexistence is known as symbiosis (Li et al., 2020b; Lee and Shin, 2020). When internal or external factors induce alterations in the microbiome, a temporary status of dysbiosis occurs. Dysbiosis pertains to the depletion or excessive proliferation of intestinal microbial populations and/or the disruption of their physiological functions. A dysbiotic microbiome has been detected in several diseases from inflammatory bowel diseases (IBDs) to cardiovascular diseases and depression (Tang et al., 2017; Khan et al., 2019).

Various pro-inflammatory markers have been detected and investigated within the last years (Vandeputte et al., 2016). Although their association with diseases that are systematic or that affect different body systems remains obscure, the “leaky gut” theory provides a formidable explanation (Obrenovich, 2018). According to this theory, alterations in the gut microbiota composition can lead to a leakage of endotoxins into the circulation that promotes systemic inflammation in addition to the development of obesity, metabolic diseases, asthma, and multiple sclerosis among others (Singh et al., 2017; Tang et al., 2017).

Localized or circulated toxins are perceived as pathogen- and microorganism-associated molecular patterns (PAMPs, MAMPs) by cellular pattern recognition receptors (PRRs). These toxins induce the production of pro-inflammatory cytokines (Negi et al., 2019). Cytokines are signaling biomolecules secreted by immune cells to affect numerous endogenous processes, including immunomodulation (Schirmer et al., 2016). Detected pro-inflammatory markers are presented in Table 1.

**TABLE 1:** Pro-inflammatory markers associated with the intestinal microbiota (Schirmer et al., 2016; Chen et al., 2017; Gou et al., 2020).

Marker	Family	Main sources	Function
Interleukin 1b	IL-1	Macrophages	Pro-inflammation, pro-differentiation, apoptosis
Interleukin 8	CXC	Macrophages, epithelial cells, monocytes	Pro-inflammation, chemotaxis, angiogenesis
Interleukin 10	IL-10	Monocytes, T cells, B cells	Anti-inflammation, inhibition of pro-inflammatory cytokines
Interleukin 12	IL-12	Dendritic cells, epithelial cells, neutrophils	Pro-inflammation, cell differentiation, NK cells activation
Tumor Necrosis Factor (TNF)	TNF	Macrophages, NK cells, adipocytes, CD4 (+) T lymphocytes	Pro-inflammation, cytokine production, cell proliferation, anti-infection
Interferon Type 1	IFN-1	Dendritic cells	Pro-inflammation, innate immunity

### 4.2 Intestinal Microbiota and Markers in COVID19: Prognosis, Diagnosis, and Treatment

The role of the microbiome in infectious diseases has been extensively studied. Despite the advances in the field, many aspects of this topic remain unknown (Negi et al., 2019; Dhar and Mohanty, 2020). Briefly, the mainstay of treatment for infections, especially antibiotics, affects the gut microbiota by decreasing the population of microorganisms that are sensitive to the prescribed medicines. In most cases, this dysbiotic condition leads to temporary gastrointestinal distress (Bernstein, 2014; He et al., 2020). At the same time, the interaction between the microorganisms and the host immune system can affect the immune response against pathogens (Rooks and Garrett, 2016; Nagpal et al., 2018).

COVID-19 seems to affect the digestive system as well, taking into account that many patients have gastrointestinal symptoms, including but not limited to vomiting and diarrhea (Xiao et al., 2020). Moreover, enterocytes express ACE-2 inhibitors and can be infected by SARS-CoV-2 (Wang J. et al., 2020; He et al., 2020). Stool diagnosis has been one of the most sensitive and specific methods for detecting SARS-CoV-2 although it is not widely used for practical reasons (Xiao et al., 2020; Zuo et al., 2020). Accumulating evidence concerns the implications of the gut microbiota in the prognosis, diagnosis, and treatment of COVID-19.

#### 4.2.1 Prognosis

Predicting the course of the COVID-19 infection is quite complex. Available evidence involves numerous factors, including gender, age, comorbidities, and clinical and laboratory findings (He et al., 2020). However, a growing body of evidence investigates the prognosis of COVID-19 in correlation with the intestinal microbiota.

Evidence from Wuhan in China suggested that the increased levels of *Lactobacillus* species correlated with higher levels of anti-inflammatory IL-10 and improved the disease prognosis (Di Renzo et al., 2020; Lee and Shin, 2020). On the other hand, the elevated levels of pro-inflammatory bacterial species, such as *Klebsiella*, *Streptococcus*, and *Ruminococcus gnavus,* correlated with the elevated levels of pro-inflammatory cytokines and infection severity (Gou et al., 2020).

Moreover, the gut microbiota seems to be involved in this condition with the so-called lung–gut axis when it comes to ARDS. Zhang et al., have recently shown that microorganisms such as *Bacteroidetes*, *Firmicutes,* and *Proteobacteria* preponderate in the lung (Rooks and Garrett, 2016; Dhar and Mohanty, 2020).

Previous studies have shown that lung infections affect the gut microbiota (He et al., 2020; van der Lelie et al., 2020). This combined evidence indicates a bidirectional axis of communication between the gut and the lung microbiota that contain endotoxins and microbial metabolites capable of affecting the gut once the lungs are infected (Anand and Mande, 2018; Dhar and Mohanty, 2020). Out of the pro-inflammatory cytokines, the expression of IFN-1 seems to mediate the crosstalk between the infected lungs and the gut (Lee and Shin, 2020; Mantlo et al., 2020). Experimental and clinical observations have already demonstrated both the principal involvement of the gut microbiota in the pathogenesis of sepsis and ARDS (Dickson, 2018; He et al., 2020) and the contribution of type I interferon to the hyperinflammation in the progression of severe COVID-19 (Lee and Shin, 2020).

It seems that the depleted microbiome and the secretion of INF-1 are associated with a poor prognosis, taking into account that elderly people who have a less diverse intestinal microbiome lacking beneficial microorganisms such as bifidobacterium are more prone to adverse outcomes.

#### 4.2.2 Diagnosis

Stool analysis of patients with COVID-19 indicates a persisting pattern of microbial disruption, even in the absence of GI manifestations and after recovering from the respiratory infection (Han et al., 2020). Their microbiota are enriched with opportunistic pathogens and depleted salutary bacteria. They also manifest an increased capacity for nucleotide and amino acid biosynthesis and carbohydrate metabolism. These findings lead to the question of whether there is a diagnostic pattern of the COVID-19-associated alterations in the microbiome (Zuo et al., 2020).

A recent study by Gu et al. suggested that comparing the microbiome alterations in COVID-19 and H1N1 could assist in distinguishing these conditions, where their similarities in a clinical presentation can trouble clinicians during winter spikes of both infections. They identified seven taxa that indicate the COVID-19 infection (Li et al., 2020b). Their findings enhance the evidence regarding the involvement of the intestinal microbiome in COVID-19; however, their clinical utility has been criticized. Microbiome analysis takes time and is expensive compared with the established methods of laboratory diagnosis of both diseases (Klann et al., 2020).

Nonetheless, stool PCR is indicated to confirm the diagnosis when SARS-CoV-2 is undetectable in the upper respiratory tract. At the same time, recent clinical studies showed that IL-1β was also markedly elevated in patients with COVID-19, particularly those admitted to the ICU.

#### 4.2.3 Treatment

In the lack of COVID-19 specific treatment, many studies have focused on repurposing existing medicines toward the pathophysiological traits of the disease (Singhal, 2020). The secretion of IL-1 leads to the dysfunction of the innate immune system, impairing the COVID-19 response. Inhibiting IL-1b, one of the microbiota-associated pro-inflammatory cytokines can be achieved using Anakinra. Anakinra is recombinant and has a non-glycosylated form of human IL-1Ra that competitively inhibits the binding of IL-1 molecules to their (IL-1R) receptor (Gao et al., 2020). Similarly, JAK inhibitors that target IL-12 and TNF-a have been recognized as a potential treatment hindering the cytokine storm in COVID-19 (Gao et al., 2020).

A recent review study published in Science has shown ambivalent results for these regimens that would be used in moderate and severe disease (Mudd et al., 2020). Several studies have examined the use of probiotics in mild disease, especially in primary home-based care management. In addition, probiotics can be used as prophylaxis for physicians and healthcare workers with constant exposure to patients with COVID-19 (Gill et al., 2001; Dhar and Mohanty, 2020) or as immunonutrition for vulnerable groups such as obese individuals (Di Renzo et al., 2020). However, more evidence is required to validate these options.

## 5 Natural Products Against SARS-CoV-2: Computational to Preclinical Studies

Natural products were searched from January 2020 to November 2020. In case of clinical studies on natural products, the timeline limit has been removed. No timeline limit has been imposed on the search for the biological sources of those phytochemicals. Though there was no keyword used related to *in silico* or computational studies, but the literature search yielded *in silico* studies as a major outcome, which is quite obvious as laboratories were not prepared enough to experimentally deal with this deadly virus, SARS-CoV-2. Globally the researchers were on a mission to explore all the possible sources against this virus, and bioinformatics and cheminformatics have indeed played a significant role, whether it is for the drug discovery or vaccine design. In this COVID-19 pandemic, it has now been widely accepted that the truly impactful and significant computational tools are utmost required to generate an experimentally feasible hypotheses, so as to accelerate the drug discovery and vaccine design programs (Galindez et al., 2021; Mohamed et al., 2021; Muratov et al., 2021). Keeping this in mind, all the *in silico*-based studies were discussed without any unbiased mind.

### 5.1 Flavonoids

**FIGURE 3:**
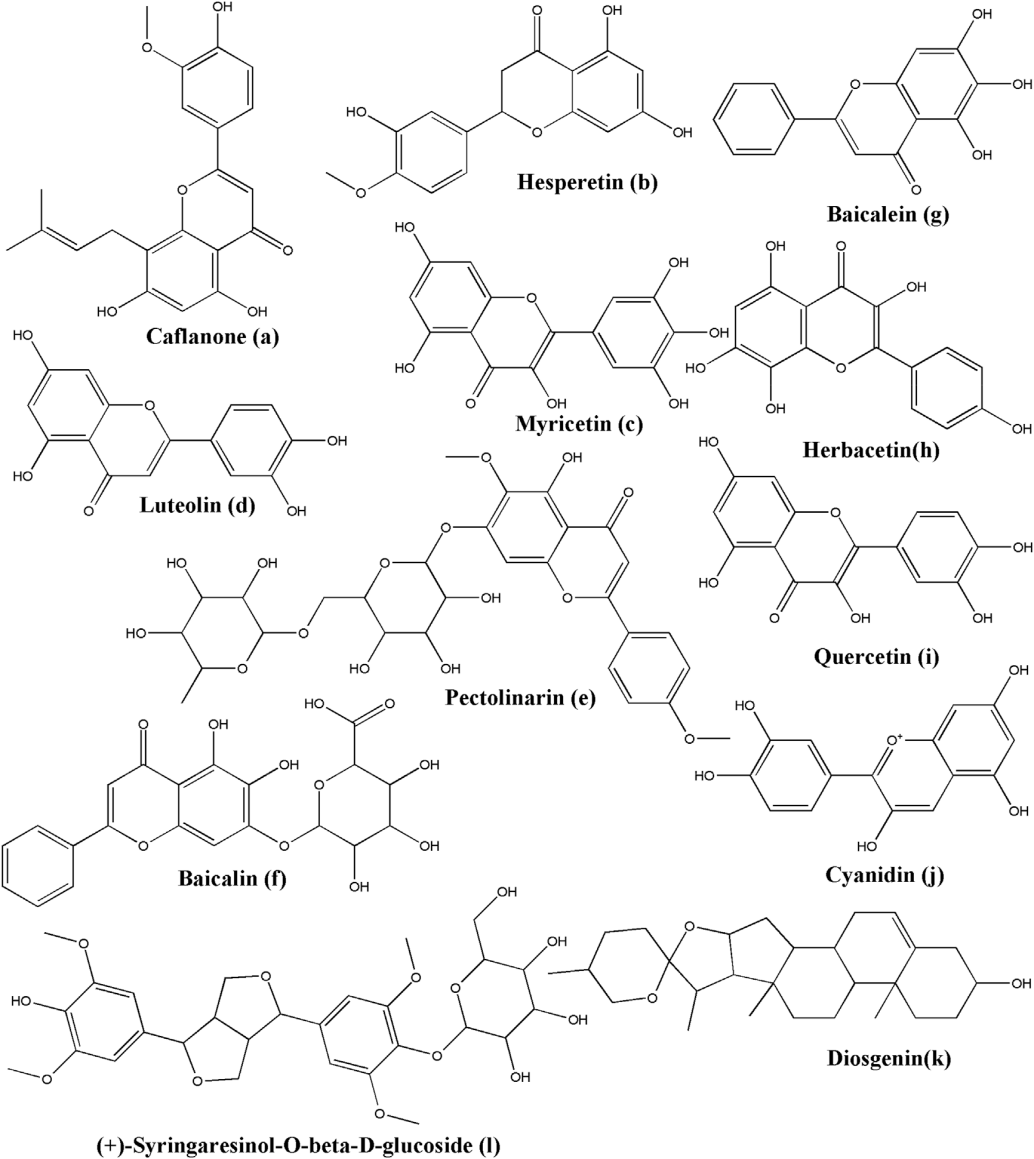
Structure of various phytochemicals with potential to tackle COVID-19.

The non-cannabinoid metabolites of *Cannabis sativa* L., caflanone (Figure 3A), were employed to establish the potential against COVID-19 and associated with the virus entry factors. Ngwa and colleagues investigated the *in silico* and *in vitro* effect of caflanone. Caflanone was docked with the ACE2 receptor (PDB ID: 1R4L) while *in vitro* antiviral activity was evaluated against the OC43 human coronavirus (hCoV-OC43). The results indicated that caflanone has a high affinity with the CoV-2 spike glycoprotein-binding sites towards the angiotensin-converting enzyme 2 (ACE2), which could inhibit the viral entry of SARS-CoV-2. Binding energy is much lower than chloroquine (CLQ) that was initially considered as prophylactics or a therapeutic anti-COVID-19 compound. Key amino acid residues in the ACE2 receptor interacting with caflanone were Arg273, Phe274, Glu375, and Zn coordinated to Glu402. *In vitro* results suggested that caflanone could inhibit hCoV-OC43 with an IC_50_ value of 0.42 µM. Moreover, they found that caflanone could decrease the expression of the viral entry-related factors, such as AXL-2, ABL-2, cathepsin L, PI4Kiiiβ, and various cytokines, viz. IL-1β, IL-6, IL-8, Mip-1α, and TNF-α (Ngwa et al., 2020).

Ngwa and colleagues investigated the *in silico* effect of hesperetin (Figure 3B) while it was docked with the ACE2 receptor (PDB ID: 1R4L) and compared with chloroquine. Hesperetin has a higher binding affinity than chloroquine towards the ACE2 receptor, which suggested its potential against COVID-19 (Ngwa et al., 2020). Hesperetin is a commonly available flavonoid found in citrus fruits, as reported by *Cordia sebestena* L. (Prakash et al., 2020) and *Origanum majorana* L. (Erenler et al., 2016).

Furthermore, Ngwa and colleagues investigated the *in silico* effect of myricetin while it was docked with the ACE2 receptor (PDB ID: 1R4L) compared with chloroquine. In a docking study, Myricetin (Figure 3C) showed better binding affinity than chloroquine (Ngwa et al., 2020). Myricetin can be isolated from many sources, including *Myrica rubra* (Lour.) Siebold and Zucc. (Wang et al., 2010), *Hypericum afrum* Lam. (Larit et al., 2021), *Abelmoschus moschatus* Medik. (Liu et al., 2005), *Tecomaria capensis* (Thunb.) Spach var. aurea (Elshamy et al., 2020), and *Moringa oleifera* Lam. (Shervington et al., 2018).

In addition, Ngwa and colleagues investigated the *in silico* effect of the linebacker while it was docked with the ACE2 receptor (PDB ID: 1R4L) and compared with chloroquine. Linebacker presented the potential of having a higher affinity with the infection-related proteins of SARS-CoV-2, which is regarded as novel prophylactics and a therapeutic natural product. It can be isolated from *Cannabis sativa* L. (Ngwa et al., 2020).

Chymotrypsin-like protease (3CLpro), papain-like protease (PLpro), RNA-dependent RNA polymerase (RdRp), and Spike (S) protein are the crucial proteins of SARS-CoV-2 that infect the host cell. Luteolin was reported to have anti-SARS-CoV activity before (Wu et al., 2004; Prasad et al., 2020). Yu *et al.*, performed the docking simulation to investigate the binding efficiency of luteolin (Figure 3D) on these proteins (PDB IDs: 6LU7 for 3CLpro; 4OVZ for PLpro; 6NUS for RdRp and 6VSB for S glycoprotein). Luteolin is the main flavonoid constituent of honeysuckle, which is the important antiviral ingredient used in traditional Chinese medicines (TCM), including Lianhuaqingwen (LH). Their results suggested that luteolin has lower binding energy and stronger interactions with the key amino acid residues than the co-crystallized ligand found in the crystal structure of these test proteins of SARS-CoV-2. Thus, it can be suggested that luteolin exhibits a potential antiviral activity (Yu et al., 2020). Luteolin can be isolated from many sources such as *Martynia annua* L. (Lodhi and Singhai, 2013), *Lonicera japonica* Thunb. (Kang et al., 2010), *Vitex negundo* L. (Rooban et al., 2012), *Colchicum ricthii* R. Br. (Abdalla et al., 1994), and *Elsholtzia rugulosa* Hemsl. (Liu R. et al., 2011).

Pectolinarin (Figure 3E) indicated its inhibitor activity with the reduction of the fluorescent intensity of 3CLpro. Its measured IC_50_ value was 51.64 µM from the curves of the concentration in the fluorescence experiment. In a docking study, Seri Jo et al. found that the L-mannopyranosyl β-D-glucopyranoside moiety and the chromen-4-one moiety of pectolinarin could capture the space of S1, S2, and S3’ sites (Aanouz et al., 2020). Pectolinarin can be isolated from *Cirsium subcoriaceum* (Less.) Sch. Bip. (Martínez-Vázquez et al., 2007), *C. chanroenicum* Nakai (Lim et al., 2008), and *C. setidens* (Dunn) Nakai (Yoo et al., 2008).

Baicalin (Figure 3F) could significantly reduce the fluorescent intensity of 3CLpro as the IC_50_ value was 34.71 µM. Baicalin binds *in silico* to Glu166, Gly143, and Asn142 by forming hydrogen bonds and His41 by pi-pi stacking (Jo et al., 2020; Mu et al., 2020). Quyuan Tao *et al.* screened all the compounds in the Huashi Baidu formula and studied the herb-compound-targets network. Consequently, they found that baicalin was the most stable active part in the docking study with 3CLpro (Tao Q. et al., 2020). Baicalin has been isolated from *Scutellaria baicalensis* Georgi (Ohkoshi et al., 2009; Peng-fei et al., 2012). Baicalein (Figure 3G), a phytoconstituent of *Polygonatum sibiricum* Redouté, could bind to the acid residues of 3CLpro, Glu166, Ser144, Gly143, Cys145, Leu141, and His163 by forming hydrogen bonds, and Gln189, Arg188, Met165, Phe140, and Asn142 by forming hydrophobic interactions (Mu et al., 2020). Baicalein can also be isolated from *Scutellariae baicalensis* Georgi Radix (Kimura et al., 2001), and *Scutellaria baicalensis* Georgi (Kimura et al., 1997). Zandi et al. had studied the anti-SARS-CoV-2 activity of baicalin and baicalein in Vero and Calu-3 cell lines and compared it with remdesivir. They found EC_50_ (µM) of baicalin, baicalein and remdesivir as 4.5, 9.0, and 1.0 respectively (in Vero cell line), and 1.2, 8.0, and 0.14 respectively (in Calu-3 cell line). Further, they had reported strong binding of baicalin and baicalein with SARS-CoV-2 RdRp, when checked by *in silico* tools. In the thermal shift assay, they found that baicalein caused a ΔTm of 3.9°C of nsp12, which suggested that baicalein is a strong and specific binder for nsp12 component of RdRp (Zandi et al., 2021).

In the fluorescence experiment, herbacetin (Figure 3H) could attenuate the intensity of the fluorescence of 3CLpro. In a docking study, the phenyl moiety of herbacetin could occupy the S1 site while the chromen-4-one moiety is located in the S2 site with hydrogen bonds (Jo et al., 2020). Herbacetin can be isolated from *Linum usitatissimum* L. (Veeramani et al., 2018), *Rhodiola rosea* L. (Péter Zomborszki et al., 2019), and *Ephedra sinica* Stapf (Hyuga et al., 2013).

In a previous study, quercetin (Figure 3I) and its 7-O-Arylmethylquercetin derivatives exerted their anti-SARS-CoV and anti-HCV *in vitro* effects (Park et al., 2012; Prasad et al., 2020). Now, a docking study indicated that quercetin could bind to ACE2 by forming hydrogen bonds with the amino acid residues Lys745, Tyr613, His493, and Asp609 (Tao Q. et al., 2020). It could also reveal a strong interaction between the main protease of SARS-CoV-2 and Glu290 and Asp289 (Vijayakumar et al., 2020). As part of the molecular mechanism exploration of Respiratory Detox Shot, Zhang and the team had performed molecular docking studies of quercetin with the 3CLpro of SARS-CoV-2 (PDB ID: 6LU7) and found that quercetin can form hydrogen bonds with His163A, Ser144A, and Cys145A (Zhang ZJ. et al., 2020). These results indicated that a novel natural product requires *in vitro* and *in vivo* further study since the molecule is effective against both the viral target and the host receptor target. Quercetin has been isolated from multiple sources, including *Euonymus alatus* (Thunb.) Siebold (Fang et al., 2008), *Rosa canina* L. (Fujii and Saito, 2014), *Diospyros kaki* L. f. (Cho et al., 2016), and *Toona sinensis* (Juss.) M. Roem. (Zhang et al., 2016). Quercetin is also readily available in various foods like onion (*Allium cepa* L.), apple (*Malus domestica* (Suckow) Borkh.), and Broccoli (*Brassica cretica* Lam.), etc (Boyer and Liu, 2004; Lombard et al., 2005; Wu et al., 2019).

The protein-ligand docking suggested that cyanidin (Figure 3J) could downregulate the RNA-dependent RNA polymerase and prevent the replication of SARS-CoV-2 by binding to the Asp761 catalytic residue (Vijayakumar et al., 2020). Cyanidin can be isolated from sources like *Prunus cerasus* L. (Wang et al., 1999) and *Oryza sativa* L. cv. Heugjinjubyeo (Hyun and Chung, 2004). There are plenty of sources where cyanidin has been isolated in its glycosidic form, though.

In a docking study, diosgenin (Figure 3K) is one of the most active components in *Polygonatum sibiricum* Redouté. A small molecule of diosgenin could form a hydrogen bond with Met276, form hydrophobic interactions between Arg131, Lys137, Asp289, Leu287, Leu286, Ala285, Gly275, or Tyr239, and 3CLpro, and form hydrophobic interactions between Phe40, Asp350, Asp382, Ala348, His378, His401, Asn394, Arg393, Tyr385, Phe390, or Trp69, and ACE2. In addition, it could form a hydrogen bond with Asn437, form hydrophobic interactions between Phe334, Lys333, Ile428, Thr431, Asn435, Tyr438, Ser336, or Ala339, and the S protein, form a hydrogen bond with Lys267, and form hydrophobic interactions between Pro461, Thr319, Val320, Phe321, Pro322, Trp268, Ile266, Tyr265, or Ser255, and the RdRp. This molecule possesses the potential against the infection of SARS-CoV-2 (Mu et al., 2020). Diosgenin has also been isolated from other sources like *Hellenia speciosa* (J.Koenig) S.R.Dutta (Selim and Al Jaouni, 2015), *Solanum virginianum* L. (Sato and Latham, 2002), *Dioscorea bulbifera* L. (Pietropaolo et al., 2014), and *Dioscorea nipponica* Makino (Kang et al., 2011).

(+)-Syringaresinol-O-beta-D-glucoside (SBG) exerts its antiviral effect through forming hydrogen bonding interactions with Glu564, Asn210, Lys94, Glu208, Asp206, Gly205, Trp203, Tyr202, and Gln102 and hydrophobic interactions with Leu91, Lys94, Ser563, Leu95, Lys562, Val212, Pro565, Val209, Trp566, and Gln98 of the ACE2 receptor (Mu et al., 2020). SBG (Figure 3L) can be isolated from *Viscum album* L. (Nazaruk and Orlikowski, 2015).

**FIGURE 4:**
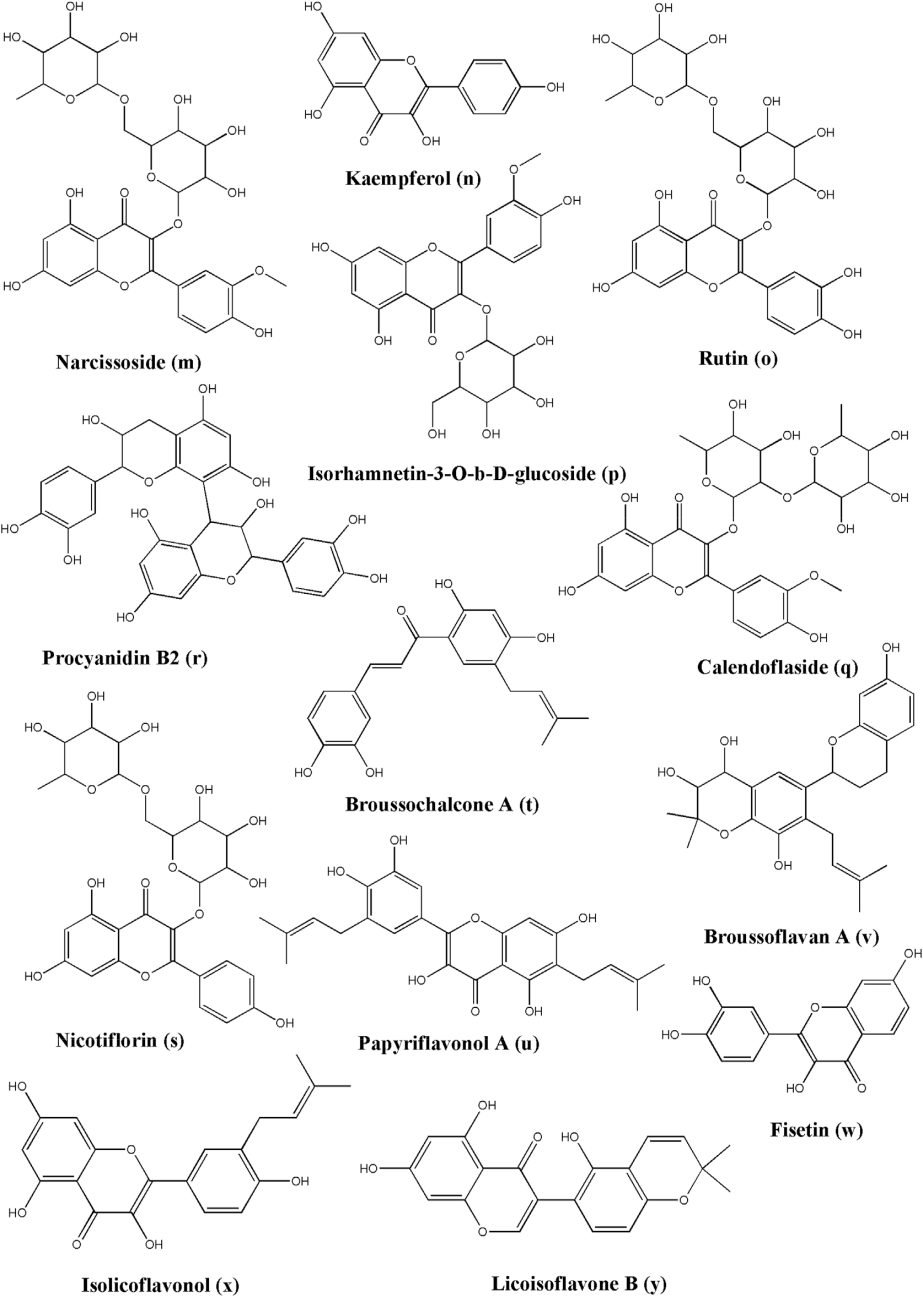
Structure of various phytochemicals with potential to tackle COVID-19.

Narcissoside (Figure 4M) has a higher affinity with the protein complex 6W63 of SARS-CoV-2 causing COVID-19 and the standard inhibitor X77. In a docking study, it could bind to Arg188, Glu166, His 164, Cys145, Asn14, Cys44, His 41, Gln192, and Thr190 by forming hydrogen bonds and exerting its potent to inhibit the activity of the COVID-19 proteins (Dubey and Dubey, 2020). Narcissoside has been reported to be found in *Azima tetracantha* Lam. (Duraipandiyan et al., 2016), *Morinda citrifolia* L. (Su et al., 2005), *Polygonatum odoratum* (Mill.) Druce (Ganbaatar et al., 2015), and *Lolium multiflorum* Lam. (Kuppusamy et al., 2018).

In docking the non-structural polypeptide, NSP25 (GVITHDVSSAINRPQIGVVREFLTR) study, kaempferol (Figure 4N) could distinctly perform interactions with Gly1 and Arg25 through forming hydrogen bonds, with Val18 through pi-sigma, and with Phe22 through the pi-pi stacked bonds (Hamza et al., 2020). TMPRSS2, a key receptor for the entry of SARS-CoV-2, is reportedly being downregulated after the treatment of the LNCaP cells with kaempferol using qPCR data as detected by Da and the team (Da et al., 2019). Kaempferol is observed in many plant sources and is even found in propolis, a resinous production by honeybees (Berretta et al., 2020). This suggested that kaempferol could serve as a potential candidate since it can act on the host receptor target as well as the viral target. Kaempferol has been isolated from multiple sources, including *Euonymus alatus* (Thunb.) Siebold (Fang et al., 2008), *Vachellia nilotica* (L.) P.J.H.Hurter and Mabb.(Singh et al., 2008), *Persicaria tinctoria* (Aiton) Spach. (Kataoka et al., 2001), *Eruca vesicaria* (L.) Cav. (Kishore et al., 2017), *Lagenaria siceraria* (Molina) Standl. (Rajput et al., 2011), and *Nelumbo nucifera* Gaertn. (Lee B. et al., 2015).

In a docking study, rutin (Figure 4O) showed the highest affinity with Mpro, which binds to Ser144, His163, Asn142, Cys145, Gly143, His41, Phe140, Thr25, Thr26, Thr190, Arg188, Met165, Glu166, His164, Leu141, and Gln189 residue sites. In addition, it possesses the potential to combat COVID-19 (Das et al., 2020). In a docking study of Felipe Moura A da Silva, rutin formed hydrogen bonds with His41, Thr25, Cys44, Met165, Gln189, and Thr190 (da Silva et al., 2020). Rutin is again a very common phytoconstituent which is widely available in a large number of resources, including but not limited to *Dendropanax morbifer* H. Lev. (Choi et al., 2015), *Schinus molle* L. (Machado et al., 2008), *Triticum aestivum* L. (Dixit, 2014), *Chrozophora tinctoria* (L.) A. Juss. (Abdel-Naim et al., 2018), *Spermacoce hispida* L. (Sundaram.R et al., 2018), *Calendula officinalis* L. (Das et al., 2020), *Edgeworthia chrysantha* Lindl. (Shengqiang et al., 2009), *Caragana spinosa* (L.) Hornem., and *Memecylon edule* Roxb. (Srinivasan et al., 2015).

Isorhamnetin-3-O-b-D-glucoside (IRG) (Figure 4P) showed high affinity, good stability, and flexibility with Mpro by binding to Cys145, Gly143, Asn142, Ser144, His163, Phe140, Gln189, Asp187, Arg188, Met165, His41, Thr26, and Met49 (Das et al., 2020). It has been reported that it is found in *Calendula officinalis* L. (Das et al., 2020), *Chrysanthemum morifolium* (Ramat.) Hemsl (Jun Hu et al., 2017), and *Salvadora persica* L.(Ali et al., 1997)*.*

Calendoflaside (Figure 4Q) showed its inhibiting function to Mpro by binding to major amino acid residues as Arg188, Asp187, Met165, His163, Ser144, Glu166, Phe140, Leu141, Cys145, Gly143, Asn142, Leu27, Met49, Gln189, and His41 (Das et al., 2020). It has been reported that it is found in *Calendula officinalis* L. (Das et al., 2020).

Procyanidin B2 revealed the lowest binding energy to 3CLpro, which has been isolated from *Uncaria tomentosa* (Willd. ex Schult.) DC. It also showed low barriers to bind in the ligand pathway simulations, that predicted inhibitory effect against SARS-CoV-2 (Yepes-Perez et al., 2020). Procyanidin B2 (Figure 4R) can also be obtained from *Malus domestica* (Suckow) Borkh. (Shoji et al., 2003), *Vitis* sp. (Yin et al., 2017), *Litchi chinensis* Sonn. (Li and Jiang, 2007), *Adansonia digitata* L. (Shahat, 2008), *Malus domestica* (Suckow) Borkh. (Hibasami et al., 2004), and *Hypericum perforatum* L. (Butterweck et al., 1998).

The special structure of procyanidin has strong interactions with the proteins of SARS-CoV-2 which could inhibit the functions and the process of infection. The binding results revealed that procyanidin in ACE2 could bind to Ser44, Ser47, Asp350, Asp382, Tyr385, Arg393, Asn394, and His401 by forming hydrogen bonds, to Phe40 and Phe390 through hydrophobic interactions, and to Asn394, Gly395, Ser43, Leu351, His378, Ala348, Trp69, Leu391, Met62, Ser47, and Asn51 through VDW interactions. In Mpro, procyanidin forms hydrogen bonds with Ser44, Ser47, Asp350, Asp382, Tyr385, Arg393, Asn394, and His401, hydrophobic interactions with Phe40 and Phe390, pi-sulfur bonds with Met49, and pi-alky interactions between the benzene ring and Cys145. In regard to the S protein, procyanidin shows that there are hydrogen bonds with Ser375, Thr376, Gly404, Asp405, Arg408, and Ile410 residues hydrophobic interactions with Thr376, Val407, and Arg408, and pi-cation and pi-anion interactions with Lys378 and Asp405, respectively. The blocking of procyanidin could effectively prevent the infection and replication of the virus (Maroli et al., 2020). Procyanidin can be isolated from *Sclerocarya birrea* (A.Rich.) Hochst. (Galvez et al., 1993), *Machaerium floribundum* Benth. (Waage et al., 1984), and *Phaseolus vulgaris* L. (Silverstein et al., 1996). Furthermore, there are numerous sources where procyanidin oligomers and their derivatives are abundantly available.

Nicotiflorin (kaempferol-3-O-rutinoside) could bind to the catalytic dyad of 3CL pro, His41, and Cys145. Furthermore, it could form hydrogen bonds with Met49, Glu166, and Thr190, form pi-pi and pi-sigma interactions with His41, and form pi-sulfur interactions with Cys145. It possesses an inhibitory effect on SARS-CoV-2 (da Silva et al., 2020). Nicotiflorin (Figure 4S) can be obtained from *Caragana spinosa* (L.) Hornem. (Olennikov and Partilkhaev, 2012), *Zeravschania aucheri* (Boiss.) Pimenov (Zahra Ahmadian et al., 2017), *Nymphaea candida* C. Presl (Zhao J. et al., 2017), *Edgeworthia chrysantha* Lindl. (Shengqiang et al., 2009), and *Brickellia cavanillesii* A. Gray (Avila-Villarreal et al., 2016).

Broussochalcone A (Figure 4T) is a kind of key polyphenol obtained from *Broussonetia papyrifera* (L.) L'Hér. ex Vent. It possesses higher affinity, higher stability, and less conformational fluctuations in the Mpro of SARS-CoV-2 than darunavir and lopinavir which are anti-HIV drugs. In a docking study, it bound to the key catalytic residues, His41 and Cys145. Furthermore, it formed hydrogen bonds with Thr26, Gly143, Ser144, Cys145, and Glu166, pi-sigma interactions with His41, pi-alkyl with Met165, and pi-sulfur interactions with Met49 to exert its potential to combat COVID-19 (Ghosh et al., 2020).

As the main content of *Broussonetia papyrifera* (L.) L'Hér. ex Vent., papyriflavonol A showed better binding energy and higher stability when it was docked with Mpro than darunavir and lopinavir as it formed hydrogen bonds with Leu141, Cys145, and Arg188, and formed pi-alkyl interactions with His41, Leu27, and Met165 (Ghosh et al., 2020). Papyriflavonol A (Figure 4U) can also be isolated from *Macaranga pruinosa* (Miq.) Müll.Arg. (Syah and Ghisalberti, 2010).

Broussoflavan A (Figure 4V) could be extracted from *Broussonetia papyrifera* (L.) L'Hér. ex Vent. The Broussoflavan A-Mpro complex showed better stability than darunavir and lopinavir due to the formation of hydrogen bonds with the residues Gly143, Glu166, and Asn143, the formation of pi-alkyl interactions with His41, Met165, and Cys145, and the formation of pi-sulfur interactions with Met49. The results predicted the promising potential of Broussoflavan A against COVID-19 (Ghosh et al., 2020).

Fisetin (Figure 4W) is a 7-hydroxyflavonol that can be obtained from various pigmented fruits and vegetables, like *Elaeagnus indica* Servett. (Srinivasan et al., 2016), *Hymenaea courbaril* L. (jatoba) (da Costa et al., 2014), and *Toxicodendron vernicifluum* (Stokes) F.A.Barkley (Lee JH. et al., 2015). In their respiratory detox shot, which is a Chinese Herbal Medicine analysis, Zhang and the team found that fisetin could make hydrogen bonds with the Cys145A amino acid residues of SARS-CoV-2 3CLpro (PDB ID: 6LU7). Therefore, fisetin can act as a potential inhibitor for this target enzyme. It is also one of the components in this Chinese Herbal Medicine (Zhang ZJ. et al., 2020).

Isolicoflavonol (Figure 4X), a flavonol analog, can be isolated from various sources, such as *Glycyrrhiza uralensis* Fisch. ex DC. (Han et al., 2012), *Broussonetia papyrifera* (L.) L'Hér. ex Vent.(Zheng et al., 2008), *Macaranga indica* Wight (Yang et al., 2015), and *Macaranga conifera* (Rchb.f. and Zoll.) Müll.Arg. (Jang et al., 2002). Besides kaempferol and fisetin, Zhang and the team have also performed a docking study on isolicoflavonol. They found that isolicoflavonol exerted a significant hydrogen bonding effect on the Ser144A, Cys145A, and His163A amino acid residues of SARS-CoV-2 3CLpro (PDB ID: 6LU7) (Zhang ZJ. et al., 2020). Therefore, isolicoflavonol can act as a potential inhibitor for this target enzyme.

Licoisoflavone B (Figure 4Y) can be traced in many plants, such as *Lupinus albus* L. (Tahara et al., 1984), *Lupinus angustifolius* L. (*Lane et al., 1987*), *Sophora moorcroftiana* (Benth.) Benth. ex Baker (Shirataki et al., 1988), and Sinkiang licorice root (Saitoh et al., 1978). Zhang and the team have performed a docking study on licoisoflavone B, along with the abovementioned natural products, viz. kaempferol, fisetin, and isolicoflavonol. They found that licoisoflavone B could make hydrogen bonds with Asn142A and Gln189A amino acid residues of SARS-CoV-2 3CLpro (PDB ID: 6LU7). This finding suggested that licoisoflavone B could serve as a potential candidate as this viral enzyme inhibitor (Zhang ZJ. et al., 2020).

### 5.2 Terpenoids

**FIGURE 5:**
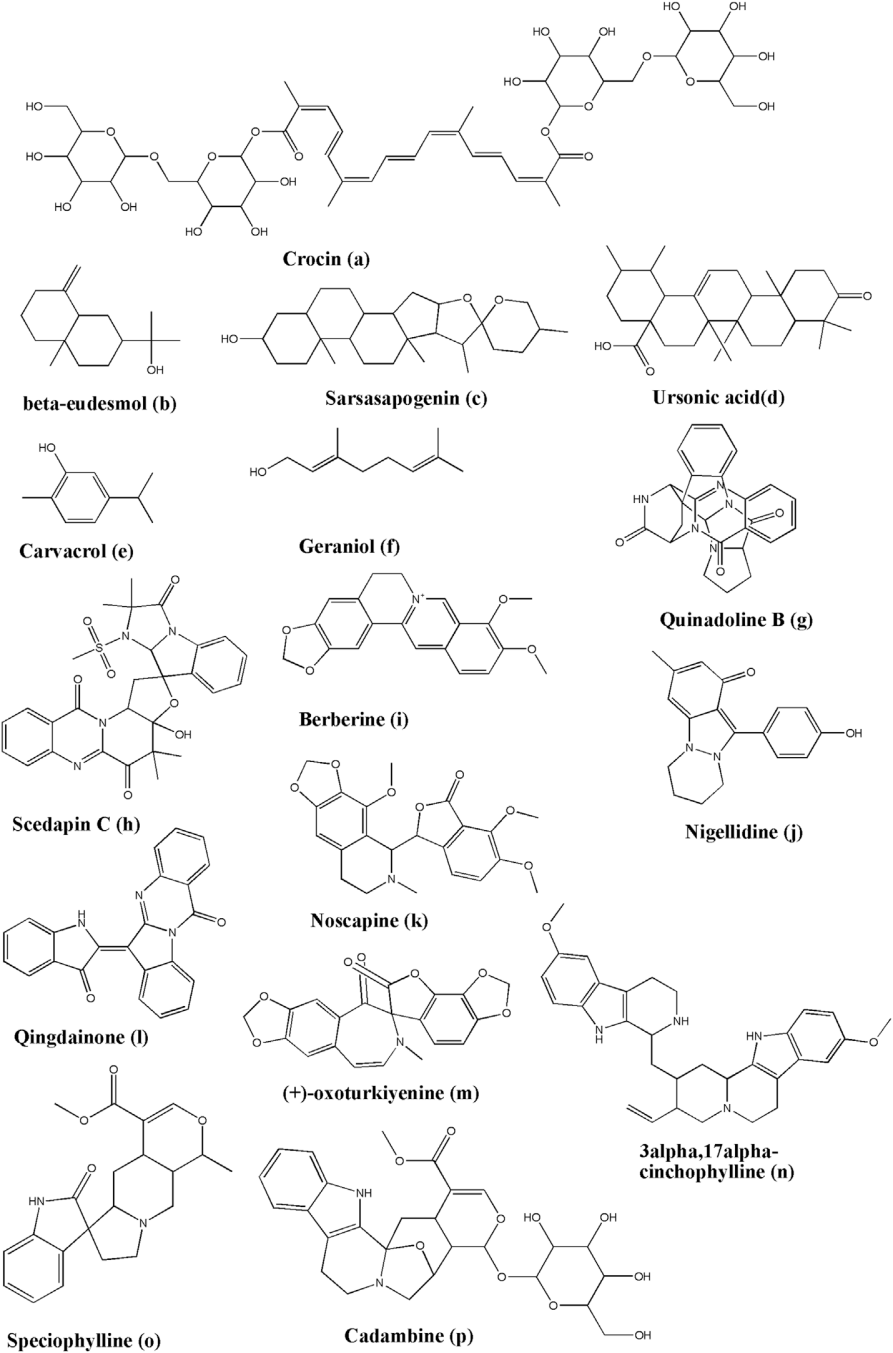
Structure of various phytochemicals with potential to tackle COVID-19.

Crocin (Figure 5A) could be extracted from *Crocus sativus* L. With its prominent effect on anti-HSV and anti-HIV drugs, crocin indicated a more promising binding energy value (−8.2 kcal/mol) with the main protease of SARS-CoV-2 than most natural products in the docking study (Aanouz et al., 2020). Another reported source for crocin is *Gardenia jasminoides* J. Ellis (Lee et al., 2005).

Even rarely isolated from *Laurus nobilis* L., β-eudesmol (Figure 5B) has antibacterial and antiviral functions. In a docking study, the β-Eudesmol binding energy value is −7.1 kcal/mol while the CLQ value is −6.0 kcal/mol against the main protease of SARS-CoV-2 (Aanouz et al., 2020). β-eudesmol can be isolated from *Zingiber zerumbet* (L.) Roscoe ex Sm. (Yu et al., 2008), *Magnolia obovata* Thunb. (Tachikawa et al., 2000), *Dioscorea japonica* Thunb. (Miyazawa et al., 1996), and *Teucrium ramosissimum* Desf. (Ben Sghaier et al., 2016).

Sarsasapogenin (Figure 5C) could be a potential inhibitor for the Nsp15 of SARS-CoV-2 by forming a strong hydrogen bond with Lys290. Its binding energy is much lower than hydroxychloroquine and chloroquine (Kumar S. et al., 2020). Sarsasapogenin can be found in *Anemarrhena asphodeloides* Bunge (Bao et al., 2007), *Asparagus officinalis* L. (Wang et al., 2011), and *Yucca glauca* Nutt. (El-Olemy et al., 1974) while glycosidic and other derivatives have been isolated from numerous other sources.

Ursonic acid (Figure 5D) also showed lower binding energy with Nsp15 than hydroxychloroquine and chloroquine. Besides, the ursonic acid and Nsp15 complex got a stable result after the MD, radius of gyration, RMSD, and RMSF studies (Kumar S. et al., 2020). Ursonic acid has been reportedly found in various sources, including *Piper betle* L. (Saeed et al., 1993), *Ziziphus jujuba* Mill. (Kawabata et al., 2017), *Ficus carica* L. (Chiang et al., 2005), *Lantana camara* L. (Begum et al., 2004), and *Catharanthus roseus* (L.) G. Don (Thanh Tam et al., 2016).

Carvacrol (Figure 5E) could form hydrogen bonds with Ser459, residue bind domain of S protein (Kulkarni et al., 2020). Carvacrol has been isolated from multiple sources, some of which are *Lippia multiflora* Moldenke (Kunle et al., 2003), *Origanum acutidens* (Hand.-Mazz.) Ietsw. (Kordali et al., 2008), *Origanum dictamnus* L. (Liolios et al., 2009), *Lippia origanoides* Kunth (Games et al., 2016), and *Thymus vulgaris* L. (Fachini-Queiroz et al., 2012).

The structure of hydroxyl with a phenyl ring indicated the activity and antiviral property of geraniol (Figure 5F). In a docking study, it could bind to Lys458 and Ser459 of the S protein by forming hydrogen bonds (Kulkarni et al., 2020). Even geraniol has been reported in numerous medicinal plants, for instance, *Pelargonium graveolens* L'Hér. (Gupta et al., 2001), *Camellia sinensis* (L.) Kuntze (Zhou et al., 2019), *Rosa × damascena* Herrm. (Sadraei et al., 2013), *Cymbopogon flexuosus* (Nees ex Steud.) W. Watson (Ganjewala and Luthra, 2009), and *Cymbopogon martini* (Roxb.) W. Watson (Kamble et al., 2020).

Glycyrrhizic acid is one of the important constituents of *Glycyrrhiza glabra* L. Previous studies on glycyrrhizic acid (glycyrrhizin) indicated that it has capability to induce interferon to prevent the replications of the MERS-CoV virus (Omrani et al., 2014; Luo et al., 2020). Maddah et al. had performed the high throughput virtual ligand screening using the dataset of 56 licorice compounds. Based on the docking studies, SAR between docking energy and ADMET properties, and MD simulations, glycyrrhizic acid was found to have highest affinity against various targets such as “spike receptor-binding domain, main protease, papain-like protease, RNA-dependent RNA polymerase, or endoribonuclease non-structural protein, as well as human angiotensin-converting enzyme 2”. This suggest that glycyrrhizic acid can be tested further to check its potential as anti-SARS-CoV-2 agent (Maddah et al., 2021).

### 5.3 Alkaloids

Quinadoline B (Figure 5G) could be extracted from the mangrove-derived fungus *Cladosporium sp*. PJX-41 that possesses anti-SARS-CoV-2 potency by binding to the Lys711 and Arg355 sites of PLpro through H-bonds and Leu557, Ala579, and Ile580 through pi-alkyl interactions. In regard to RdRp, quinadoline B showed the highest affinity with the binding sites, by binding to Gln73 through H-bonds, to Arg569 through pi-cation, to Ala686 through pi-alkyl interactions, and to Tyr689, Ala580, and Ala688 sites through pi-pi stacking and pi-alkyl interactions. Concerning nsp15, it could be bound to His235 and His250 through van der Waals (VDW) affinity, to Lys290 through the pi-cation intermolecular bonding, to Tyr343, Lys345, and Leu346 through pi-pi stacking/pi-alkyl interactions. In addition, nsp15 exerts an H-bonding effect on the Val292 site. Regarding the S protein, it interacted with binding sites through pi-sulfur bonding to Cys454, pi-anion to Asp441, pi-alkyl to Ala444, and pi-pi stacking to Phe430. With the ADMET results, quinadoline B indicated high gastrointestinal (GI) absorption, low blood-brain barrier penetrability, and high drug-likeness (Quimque et al., 2020). Quinadoline B has also been extracted from *Aspergillus giganteus* Wehmer, 1901 NTU967 which was isolated from the marine alga, *Ulva lactuca* (Chen JJ. et al., 2020), and *Aspergillus* sp. FKI-1746 (Koyama et al., 2008).

In the compounds of fungal secondary metabolites, scedapin C (Figure 5H) could be isolated from the marine-derived fungus *Scedosporium apiospermum* (Sacc.) Sacc. ex Castell. and Chalm., 1919 F41-1, exerting the highest affinity with PLpro through various interactions, viz. hydrogen bonding with Arg712, pi-cation interactions with Lys711, pi-pi stacking interactions with His342, and pi-alkyl interactions with Ala579. Concerning 3CLpro, scedapin C could bind to Cys145 through pi-sulfur interactions, Met165 through pi-pi stacking interactions, His41 through pi-pi stacking interactions, and Met49 through pi-alkyl interactions. Compared with favipiravir, RdRp has higher binding energy by binding to Lys593 and Cys813 through hydrogen bonds, Ile589 and Leu758 through pi-alkyl interactions, and Cys813 through pi-sulfur interactions. In regard to nsp15, scedapin C hinged itself on His235 through pi-pi stacking interactions, His250 and Lys290 through VDW affinity, Thr341 through H-bonds, and Tyr343 through pi-pi stacking interactions (Quimque et al., 2020).

Berberine (Figure 5I) could be extracted from the root, rhizomes, stems, and the bark of *Hydrastis canadensis* L. (Berberidaceae). After the viral screening and the docking study of the potential inhibition against 3CLpro, the main protease in SARS-CoV-2, it showed much lower binding energy to 3CLpro, compared with other compounds isolated from *Tinospora cordifolia* (Willd.) Hook. f. and Thomson. In addition, the berberine:3CLpro structure possesses higher stability than other inhibitors according to the MD simulation and exerts a potent effect against COVID-19 by preventing the activity of 3CLpro (Chowdhury, 2020). Other reported biological sources, where berberine is one of the important phytoconstituents, are *Berberis vulgaris* L. (Freile et al., 2003), *Berberis aquifolium* Pursh (Čerňáková and Košťálová, 2008), *Berberis vulgaris* L. (Imanshahidi and Hosseinzadeh, 2008), and *Corydalis chaerophylla* DC. (Basha et al., 2002).

Nigellidine is a bioactive component obtained from the seeds of *Nigella sativa L.*, which was reported before for its anti-oxidative, anti-inflammatory, anti-bacterial, anti-hypertensive, and immunomodulatory functions. In the docking study of Maiti and workers, nigellidine (Figure 5J) could interdict the function of the Nucleocapsid (N) protein of SARS-CoV-2 by binding to Ala55 (through hydrogen bonds), Gln306 (through N-O bonds), and ARG203, ARG209, Leu230, Gln241, Gln242, Ala308, Ala305, and Phe307 residue sites. In regard to the Nsp2 of SARS-CoV-2, which could concern the integrity of mitochondria and the resistance to the diverse stresses of the host cell, nigellidine could block it by binding to Cys240 through rigid bonds, and Leu169, Val126, Trp243, Ala127, Cys132, The256, Gly257, Tyr242, Val157, and other positions with Ala 241 through hydrogen bonds. Concerning Mpro, nigellidine could form a stable bond with Glu166 (Maiti et al., 2020).

Noscapine (Figure 5K) has a higher affinity and a much lower binding score to the pocket-3 of Mpro, compared with chloroquine, ribavirin, and favipiravir. It formed hydrogen bonds with Thr199 and Asn238, and hydrophobic interactions with Asp197, Thr198, Thr199, Leu237, Asn238, Tyr239, and Leu271 *in silico*. Furthermore, the results of the molecular dynamic simulation revealed that noscapine possessed good stability and conformational change. Additionally, it was a potential natural product against SARS-CoV-2 (Kumar N. et al., 2020). Apart from the natural source *Papaver somniferum* L. (Dang and Facchini, 2012) from which it is abundantly isolated, there is enough literature available on noscapine and the synthesis of its derivatives (Zhou et al., 2003; Ni et al., 2011; Devine et al., 2018).

Transmembrane protease Serine 2 (TMPRSS2) is the essential receptor of the host cell that could modulate the entry of SARS-CoV-2. Vivek-Ananth *et al.* studied the affinity of qingdainone (Figure 5L) to TMPRSS2. With the lowest binding energy, qingdainone could form hydrogen bonds with D440 and A399 as well as hydrophobic interactions with I381, S382, T387, E388, N398, A400, D440, C465, and A466 (Vivek-Ananth et al., 2020). Qingdainone is also well known as candidine. It can be isolated from sources such as *Yarrowia lipolytica* (Jahng, 2013), *Isatis tinctoria* L. (Zou and Huang, 1985; Wu et al., 2007), and *Strobilanthes cusia* (Nees) Kuntze (Zou and Huang, 1985).

(+)-Oxoturkiyenine has lower binding energy to cathepsin L which is an essential receptor of the host cell for the entry of SARS-CoV-2. The residues of cathepsin L, such as Q19 and W189, could form hydrogen bonds with (+)-oxoturkiyenine (Figure 5M), pi-pi interactions with W189, and hydrophobic interactions with G139, H140, H163, and W189 (Vivek-Ananth et al., 2020) (+)-Oxoturkiyenine can be isolated from *Hypecoum pendulum* L. (Kadan et al., 2004; Mete and Gözler, 2004).

3α,17α-Cinchophylline could be extracted from *Cinchona calisaya* Wedd., the herb that possesses antiviral and anti-inflammatory activities. In regard to cathepsin L, the receptor of the host cell which plays the key role in the process of SARS-CoV-2 entry, 3α,17α-cinchophylline (Figure 5N) formed hydrogen bonds with C25, H163, G23, and M70, and hydrophobic interactions with Q21, C22, L69, M70, A135 and W189 to reveal its potential function for COVID-19 (Vivek-Ananth et al., 2020).

Speciophylline could be extracted from *Uncaria tomentosa* (Willd. ex Schult.) DC. It exerts a higher affinity with 3CLpro compared with N3, the inhibitor of 3CLpro as it is known. To the S1 cleavage site, speciophylline (Figure 5O) performs its affinity without obviously energetic expend (Yepes-Perez et al., 2020). It has also been reported that it is isolated from *Mitragyna speciosa* Korth. (Beckett et al., 1965), *Uncaria lanosa* f. philippinensis (Elmer) Ridsdale (Olivar et al., 2018), *Uncaria bernaysii* F. Muell. (Phillipson and Hemingway, 1973), and *Uncaria attenuata* Korth. (David Phillipson and Hemingway, 1975).

Cadambine comes from *Uncaria tomentosa* (Willd. ex Schult.) DC. It possesses a significant affinity with 3CLpro. Furthermore, the ligand-pathway simulation study showed low barriers to bind in the case of this test molecule. Thus, cadambine (Figure 5P) could be a potent inhibitor of SARS-CoV-2 (Yepes-Perez et al., 2020). It can be isolated from *Neolamarckia cadamba* (Roxb.) Bosser (Kumar et al., 2015), *Neonauclea purpurea* (Roxb.) Merr. (Handa et al., 2004), and *Uncaria rhynchophylla* (Miq.) Miq. (Qi et al., 2014).

### 5.4 Glycosides

**FIGURE 6:**
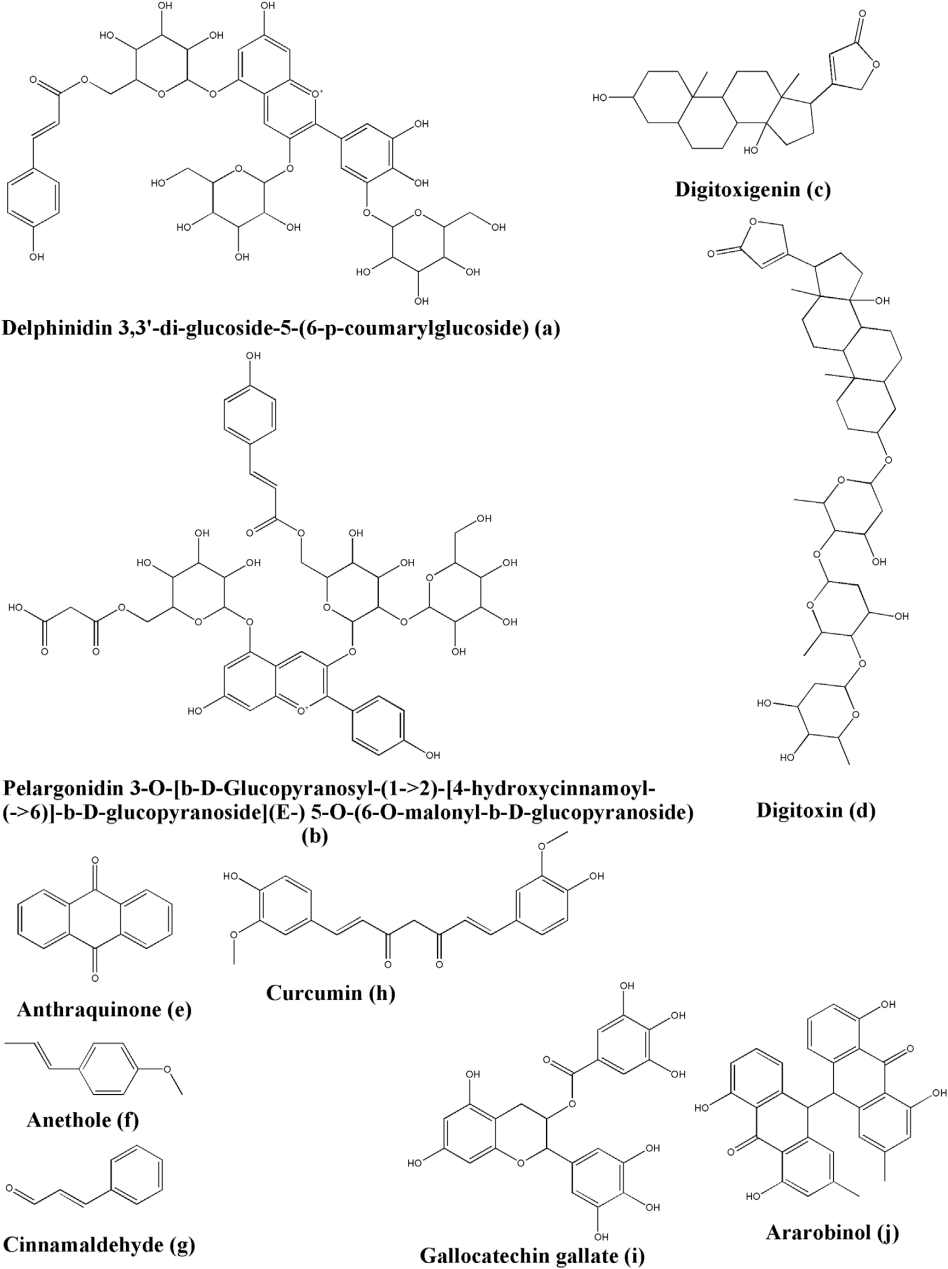
Structure of various phytochemicals with potential to tackle COVID-19.

As an anthocyanin derivative, delphinidin 3,3′-di-glucoside-5-(6-*p*-coumarylglucoside) (DGCG) (Figure 6A), displayed a potential function to interdict the main protease of SARS-CoV-2 according to the molecular dynamic simulation, the radius of gyration analysis, and the binding of free energy results (Fakhar et al., 2020). DGCG has been reportedly isolated from *Gentiana* cv. Albireo (Hosokawa et al., 1997).

Pelargonidin 3-O-[β-D-Glucopyranosyl-(1->2)-[4-hydroxycinnamoyl-(->6)]-β-D-glucopyranoside](E-) 5-O-(6-O-malonyl-β-D-glucopyranoside), PGHGM (Figure 6B) is another derivative of anthocyanin with activity against the main protease of SARS-CoV-2 as per the results obtained by the radius of gyration, the binding of free energy, the molecule stability, and the flexibility studies (Fakhar et al., 2020). PGHGM can be isolated from *Pomacea maculata* Perry, 1810 (KHALIL et al., 2020).

From the *Nerium oleander* L., digitoxigenin (Figure 6C) and its derivatives exert antiviral and anti-cancer properties. It has a binding energy value of −7.2 kcal/mol and is proposed to be an effective inhibitor to the coronavirus against the main protease of SARS-CoV-2 (Aanouz et al., 2020). Another important and main source where digitoxigenin can be isolated is *Digitalis lanata* Ehrh. (Caspi and Hornby, 1968).

In the screening study of the DrugBank dataset, digitoxin (Figure 6D) revealed the lowest binding energy with Site 2 of the S protein of SARS-CoV-2. It formed hydrogen bonds with Lys458, Ser459, Asp467, and Glu471, and carbon-hydrogen bonds with Lys458 and Glu471. Furthermore, it formed alkyl hydrophobic interactions with Lys458 and Pro491 (Wei TZ. et al., 2020). Clinically relevant, digitoxin can be isolated from *Digitalis purpurea* L. (Hagimori et al., 1984).

### 5.5 Quinones

The results of the docking study by Hamza *et al.*, suggested that anthraquinone (Figure 6E) may have an inhibitory effect against COVID-19 by being bound to non-structural polypeptides (GVITHDVSSAINRPQIGVVREFLTR) amino acid residues, such as Val2 (through hydrogen bonds), Ile3 (through hydrogen bonds), and Gly1 (through pi-cation interactions) (Hamza et al., 2020). Anthraquinone is such an important scaffold with many natural derivatives. Consequently, it becomes a separate class of compounds.

### 5.6 Monolignols

Anethole (Figure 6F) could bind to Ser459 of the S protein by forming hydrogen bonds, which are rich in some plant families such as Apiaceae, Myrtaceae, and Fabaceae (Kulkarni et al., 2020). Some of the biological sources of anethole are *Foeniculum vulgare* Mill. (Dongare et al., 2012), *Pimpinella anisum* L. (Kubo et al., 2008), *Illicium verum* Hook. f. (Liu, 1996), *Croton grewioides* Baill. (de Siqueira et al., 2006), and *Vepris madagascarica* (Baillon) H. Perier (Rabehaja et al., 2013).

Cinnamaldehyde has a high ability to fight against inflammation, viruses and cancer. In a docking study, cinnamaldehyde could form hydrogen bonds with Glu471 and Arg454 and the key residues of the S protein. It also displays the capacity for preventing the infection process of SARS-CoV-2 (Kulkarni et al., 2020). Cinnamaldehyde (Figure 6G) has been tracked in multiple sources, including but not limited to *Cinnamomum verum* J. Presl (Kakinuma et al., 1984; Al-Bayati and Mohammed, 2009; Liu et al., 2014).

### 5.7 Phenolic and Polyphenolic Compounds

Previous studies indicated that curcumin (Figure 6H) which is the most important phytoconstituent in turmeric (*Curcuma longa* L.) (Anderson et al., 2000) has a potential effect against AIDS inhibiting the HIV protease and integrase enzymes, along with having a synergistic action with antiretroviral drugs (Prasad and Tyagi, 2015; Gupta et al., 2020). In the case of the influenza A virus, curcumin reportedly reduces inflammatory cytokines (Ciavarella et al., 2020; Gupta et al., 2020). In the case of H1N1, it was found that it decreases the nucleoprotein expression, thereby preventing the infection of the influenza virus (Richart et al., 2018; Lai Y. et al., 2020; Gupta et al., 2020). All these findings strongly suggested the potent antiviral activity inherently possessed by curcumin. This has led Oso and the team to check the affinity of curcumin against COVID-19-associated proteases, such as cathepsin K, COVID-19 main protease, and SARS-CoV 3C-like protease, by performing *in silico* studies. Their results suggested that curcumin has strong binding affinities towards all the target proteins, with the best against the SARS-CoV 3C-like protease. Interaction analysis performed by Oso and the team further suggested that curcumin could form hydrogen bonding with the Trp188 of cathepsin K while it could form hydrogen bonding with Gly143 and Ser144 of the COVID-19 main protease. Furthermore, curcumin was found to form hydrogen bonding with Gly109, Gln110, Thr111, and Phe294 of the SARS-CoV 3C-like protease as per their analysis (Oso et al., 2020).

Syn-16 is the coumarin derivative that exhibited the potential for combating COVID-19. After the structure-based virtual screening, molecular dynamics simulation, and the binding of free energy calculation, Khan and workers found that Syn-16 could form three different hydroxyl groups of hydrogen bonds and have stable interactions with the S1, S2, and S5 pocket residues. Thus, Syn-16 displayed the promising potential that it could bind to 3CLpro and prevent the replication and maturation of SARS-CoV-2 (Khan et al., 2020).

Gallocatechin gallate (Figure 6I), a derivative obtained from *Saxifraga spinulosa* Adams, 1817, non Royle, 1835*,* was reported about its function in inactivating the influenza A virus and norovirus. Takeda and the team studied its capacity for fighting against SARS-CoV-2. The results suggested that a pyrogallol-enriched fraction (Fr 1C) inactivated 99.53% of SARS-CoV-2 with 10s of exposure, decreased the S2 subunit of the S protein, interdicted the cDNA reverse transcription more rapidly than any other fractions (Takeda et al., 2020). Gallocatechin gallate is available in *Camellia sinensis* (L.) Kuntze (Sugita-Konishi et al., 1999), and *Diospyros kaki* L. f. (Matsuo and Ito, 2014).

Ararobinol showed the highest affinity towards cathepsin L in the docking study. Earlier studies indicated that ararobinol (Figure 6J) has antiviral properties. Ararobinol can build hydrogen bonds with cathepsin L residues, such as Q19 and A138, pi-pi interactions with W189, and hydrophobic interactions with C25, G139, L144, H163, and W189 (Vivek-Ananth et al., 2020). Ararobinol could be found in *Senna occidentalis* (L.) Link. It can also be isolated from sources like *Frangula caroliniana* (Walter) A. Gray (Mekala et al., 2017) and *Senna siamea* (Lam.) H.S.Irwin and Barneby (Kumar et al., 2017).

**FIGURE 7:**
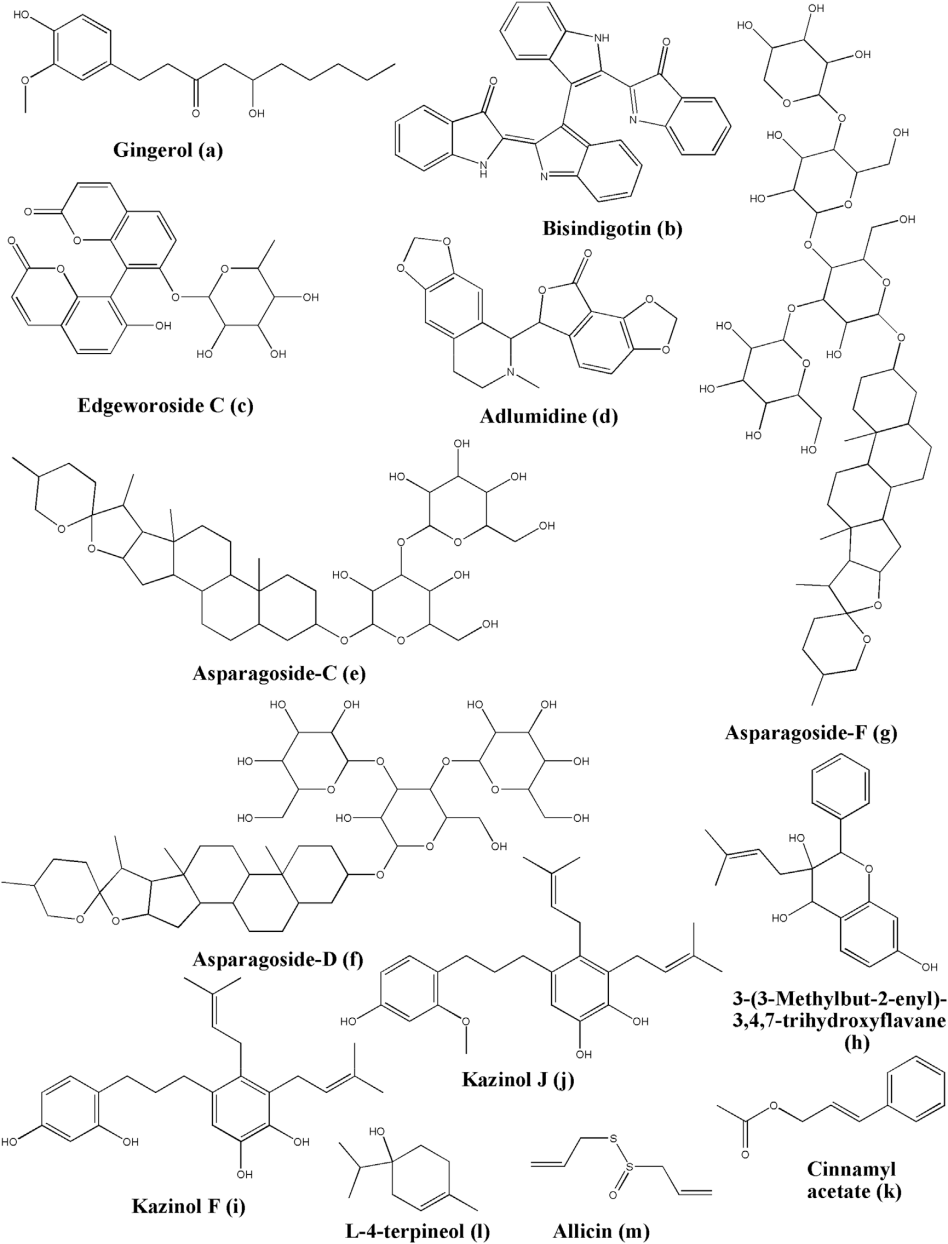
Structure of various phytochemicals with potential to tackle COVID-19.

Gingerol (Figure 7A), which is an important phytoconstituent of *Zingiber officinale* Roscoe (Guh et al., 1995), has also been investigated by means of cheminformatics by Oso and the team for its binding affinity and potential against COVID-19-associated proteases, like cathepsin K, COVID-19 main protease, and SARS-CoV 3C-like protease. Their results suggested that gingerol also had a good binding affinity with all these target enzymes, especially Cathepsin K. Their further performed studies indicated that gingerol could form hydrogen bonding with Asn18, Gln19, His162, Trp184, and Trp188 amino acid residues of Cathepsin K. It also has the potential to form hydrogen bonding with Thr199, Leu272, and Leu287 amino acid residues of the COVID-19 main protease. Additionally, they found it has the potential to form hydrogen bonding with Thr111 and Thr292 of the SARS-CoV 3C-like protease (Oso et al., 2020). Gingerol has found in *Aframomum melegueta* K. Schum. (Mohammed et al., 2017).

In the simulation, Nat-1 (coumarin analog) had a pi-alkyl interaction with Gln189, which is in the S5 pocket residues with different hydroxyl groups. The binding model indicated that there are interactions between Nat-1 and 3CLpro, which could contribute to the new treatments of the SARS-COV-2 infection (Khan et al., 2020).

### 5.8 Miscellaneous Compounds

Isochaetochromin D1 is a kind of *Fusarium sp*. metabolites that has an interfering function in viral enzymes. In regard to the non-structural protein 15 (nsp15) of SARS-CoV-2, it could bind to Val292 and His250 through H-bonding, His235, and Lys290 through VDW interactions, and other sites through pi interactions to interdict the activity of nsp15 (Quimque et al., 2020).

Bisindigotin (Figure 7B) can be extracted from *Isatis tinctoria* L. (Mohn et al., 2009) and *Persicaria tinctoria* (Aiton) Spach. In the screening study of the Traditional Chinese Medicine Systems Pharmacology (TCMSP), bisindigotin exerted the lowest binding energy with the S protein that binds to Arg457, Ser469, and Glu471 through hydrogen bonds, Lys458 through carbon-hydrogen bonds, Asp467 and Glu471 through pi-anion interactions, and Arg457 through pi-alkyl interactions, which increased the stability of the binding (Wei TZ. et al., 2020).

Edgeworoside C could be isolated from *Edgeworthia gardneri* (Wall.) Meisn. and widely used for the treatment of metabolic diseases. In a docking study, edgeworoside C (Figure 7C) could form hydrogen bonds with A386, N398, A399, V434, D435, D440, D435, V434, A386, N398, and D440 of TMPRSS2, and bind to E260, I381, A400, N433, and A466 through hydrophobic interactions to exhibit its antiviral properties (Vivek-Ananth et al., 2020). Edgeworoside C has been isolated from *Edgeworthia chrysantha* Lindl. (Yan et al., 2004).

Adlumidine (Figure 7D) is the main constituent of *Fumaria indica* (Hausskn.) Pugsley (Blaskó et al., 2004) which could treat cough, fever, and skin and urinary-related diseases. The study suggested that adlumidine has a high affinity with the TMPRSS2 which is the key target for the entry of SARS-CoV-2. The complex has hydrogen bonds between adlumidine and E388, E389, S436, C465, C437, and A466 while it has hydrophobic interactions with E260, I381, S382, T387, N398, A399, and A400 (Vivek-Ananth et al., 2020). Previous literature suggested that adlumidine can be obtained from *Pseudofumaria lutea* (L.) Borkh. (Yang et al., 1993), and *Dactylicapnos torulosa* (Hook.f. and Thomson) Hutch. (Rücker et al., 1994).

Asparagoside-C (Figure 7E) has a higher affinity with the S protein of SARS-CoV-2. It could be extracted from *Asparagus racemosus* Willd. The molecular dynamic simulation results suggested that asparagoside-C and S protein possess a stable conformation, caused by hydrogen bonds with Gly496, Gln414, Ser494, Thr415, and Tyr453. Concerning the nucleocapsid protein (N protein), it is also observed that it forms hydrogen bonds with Glu234, Gly230, Val292, His235, and Asp240 (Chikhale et al., 2020).

Asparagoside-D (Figure 7F) is also an important phytoconstituent obtained from *Asparagus racemosus* Willd. It has a better binding energy result than the standard drug Remdesivir and this is indicated in the treatment regimen for COVID-19 right now. Asparagoside-D could form hydrogen bonds with Gly502, Ser494, Lys417, Asp420 Tyr449, and Gln498 of the S protein and with Glu340, His243, Gln245, Asp240, Asn278, and Leu346 of the N protein in SARS-CoV-2. Thus, it has a major potential for acting against COVID-19 (Chikhale et al., 2020).

Asparagoside-F (Figure 7G) is another important phytoconstituent obtained from *Asparagus racemosus* Willd. It has better affinity and stability because of hydrogen bonds formed between the N and Glu234, Gly230, Ala232, Hip235, Asp240, Glu340, and Val339. This displays the capacity for blocking the key protein of SARS-CoV-2 (Chikhale et al., 2020).

3-(3-Methylbut-2-enyl)-3,4,7-trihydroxyflavane (MTHF) (Figure 7H), could be isolated from *Broussonetia papyrifera* (L.) L'Hér. ex Vent.*.* It possesses a better blocking capacity for the Mpro of SARS-CoV-2 than darunavir and lopinavir. The docking study indicated that it could form a highly stable and less fluctuated complex with Mpro, by binding to Leu141, Asn142, Gly143, Cys145, and Glu166 through forming hydrogen bonds, Met49 through pi-sulfur and pi-alkyl interactions, and His41 through pi-sigma and pi-alkyl interactions (Ghosh et al., 2020).

Kazinol F (Figure 7I) revealed that it has the lowest binding energy value among all the constituents of *Broussonetia papyrifera* (L.) L'Hér. ex Vent. by forming hydrogen bonds with Leu141, Gly143, and Met165 amino acid residues in Mpro, pi-alkyl interactions with Cys145 and Met49, pi-pi T-shaped interactions with His41, and the key catalytic residue of Mpro (Ghosh et al., 2020). Another source for isolating Kazinol F is *Broussonetia × kazinoki* Siebold (Baek et al., 2009).

Kazinol J (Figure 7J) has been isolated from *Broussonetia papyrifera* (L.) L'Hér. ex Vent. It showed a lower binding energy value, higher affinity, higher stability, and less fluctuation when it bound with Mpro compared with darunavir and lopinavir. kazinol J occupied the *in silico* residues, such as Ser144, His163, and Thr190 through forming hydrogen bonds, Met49, Met165, Pro168, and Cys145 through pi-alkyl interactions, and His41 through pi-sigma interactions (Ghosh et al., 2020).

Cinnamyl acetate (Figure 7K) showed its anti-SARS-CoV-2 potential by binding with Glu471, Arg454, and Ser459 of the S protein through H-bond interactions (Kulkarni et al., 2020). Cinnamyl acetate is mainly obtained from *Cinnamomum verum* J. Presl (Choi et al., 2001; Kaul et al., 2003), and *Cinnamomum osmophloeum* Kaneh. (Cheng SS. et al., 2006).

L-4-terpineol (Figure 7L) could be extracted from the essential oil of tea tree and lavender. It can bind to the S protein by forming hydrogen bonds with Leu492 and Tyr505 (Kulkarni et al., 2020). Some of the other reported biological sources are *Artemisia herba-alba* Asso (Nezhadali et al., 2008), *Pistacia chinensis subsp. integerrima* (J.L.Stewart) Rech. f. (Shirole et al., 2015), *Artemisia nanschanica* Krasch. (Shang et al., 2012), and *Nigella sativa* L. (Liu et al., 2013).

Allicin (Figure 7M) is a sulfoxide derivative that is categorized under sulfinic acids. It is one of the very important phytoconstituent found in *Allium sativum* L. (garlic). Oso and the team performed simulation studies to assess the binding potential of allicin to various targets of SARS-COV-2, viz. cathepsin K, COVID-19 main protease, and SARS-CoV 3C-like protease. Their results suggested that allicin elicited a similar sort of binding affinity towards all these tested proteins. Allicin could form hydrogen bonding with Gly66 of cathepsin K or Gly143 and Ser144 of the COVID-19 main protease, and Thr190 of the SARS-CoV 3C-like protease (Oso et al., 2020).

## 6 Translational Potential of Natural Products Against SARS-CoV-2: Bench to Bedside

### 6.1 Lianhua Qingwen

Lianhua Qingwen (LHQW) capsule contains so many different kinds of natural product extracts, such as “*Forsythia suspensa* (Thunb.) Vahl. (Lianqiao), *Lonicera japonica* Thunb. (Jinyinhua), *Ephedra sinica* Stapf (Mahuang), *Prunus armeniaca* L (Kuxingren), *Gypsum fibrosuum* (Shigao), *Isatis tinctoria* L. (Banlangen), *Dryopteris crassirhizoma* Nakai (Mianmaguanzhong), *Houttuynia cordata* Thunb (Yuxingcao), *Pogostemon cablin* (Blanco) Benth. (Guanghuoxiang), *Rheum palmatum* L. (Dahuang), *Rhodiola rosea* Linn. (Hongjingtian), *Mentha canadensis* L. (Bohe), *Glycyrrhiza uralensis* Fisch. ex DC. (Gancao)”, which reportedly affect COVID-19 (Li L.-C. et al., 2020). Zheng *et al.*, studied the mechanism of action of LHQW in COVID-19. Their analysis indicated that most of the constituents are modulating the expression of the lung proteins and having a relationship with more than 2,000 targets, 160,000 protein-protein interactions, and 30 functional modules. LHQW is modulating 189 proteins that are related to the co-expression of ACE2, thus concerning its ability to repair lung damage, attenuate the cytokine storm, and alleviate the symptoms caused by the ACE2-expression disease (Zheng et al., 2020). In a clinical study of efficacy and safety from Hu and the workers, they found that the treatment group has a higher recovery rate, improvement in chest, computed tomography manifestations rate, and clinical cure rate, but it has a shorter recovery time from symptoms like fever, cough, and fatigue. In this study, the results suggested a natural-product-combination-based capsule contributes to attenuating the symptoms of COVID-19 in clinical environments (Hu et al., 2020).

### 6.2 Pudilan

The formula of pudilan (PLD) contains dandelion, Isatis root, *Scutellaria baicalensis* Georgi, and *Corydalis bungeana* Turcz. herb. This polyherbal formulation is used in clinical settings as anti-SARS CoV-2 in China. Kong and the workers studied its efficacy against COVID-19. The ingredients’ data analysis results indicated that PLD could prevent the entry of SARS-CoV-2 by blocking ACE2, modulating the immune-related factors and proteins to relieve the cytokine storm, and attenuating the inflammation. Thus, PLD can alleviate the symptoms and exert its potency for the treatment of COVID-19 (Kong et al., 2020).

### 6.3 Chinese Herbs Mixture

In one patient infected with COVID-19, Lan-ting Tao and his co-workers performed a form of Traditional Chinese Therapy including a combination of acupuncture and a preparation consisting of Chinese herbs were used for the treatment. Regarding the introduction, the formula contains *Aconitum carmichaeli* Debeaux lateralis praeparata, Radix et *Glycyrrhiza glabra* L. praeparata cum Melle, *Lonicera japonica* Thunb., *Gleditsia sinensis* Lam., *Ipomoea cairica* (L.) Sweet, *Citrus × aurantium* L., and *Agastache rugosa* (Fisch. and C.A.Mey.) Kuntze that could enhance immune mechanism as anti-pathogenic qi and rejuvenate the functionality of the lung. The results of the treatment indicated that the therapy attenuated symptoms to less cough and sputum, relieved shortness of breath on exertion, and decreased shadows of CT images. Furthermore, the patient felt much better and returned to their previous condition. According to their analysis, the formula alleviated the lung by modulating the kidney qi and the toned spleen and stomach, promoting immunity, preventing transmission of the pathogen, and recovering the host system and turning it back to the normal level (Tao LT. et al., 2020).

### 6.4 Chinese Traditional Medicine Prescription

One 23-year-old infected male was studied by Qian Liu and the team. Before the intervention, the patient presented with diarrhoea (2-days history), pneumonia, and liver damage, but there were no fever and cough. The prescription contained almond, *Lophatherum gracile* Brongn., tuckahoe (*Wolfiporia aff. extensa*), forsythia (*Forsythia suspensa* (Thunb.) Vahl.), *Wurfbainia villosa* (Lour.) Skornick. and A.D.Poulsen, hawthorn (*Crataegus sp.*), medicated leaven (Massa Fermentata Medicinalis), malt (*Hordeum vulgare* L.), and *Pueraria montana* var. lobata (Willd.) Maesen and S.M.Almeida ex Sanjappa & Predeep. Following treatment, CT imaging was cleared of the typical signs of pneumonia. Recovery was also documented by means of a negative nucleic acid test, the positive IgG, and the IgM results (Liu Q. et al., 2020).

### 6.5 Qing-Fei-Da-Yuan

QFDY is the granular formulation under traditional Chinese medicines. It is used by the clinical experts of Hubei Province for COVID-19 patients under the emergency response mechanism. Hong and the team performed the network pharmacology and molecular docking studies with the key components of this formulation and the COVID-19 targets. They hypothesized that QFDY acts multimodally by regulating ACE2’s co-expressing genes, inflammation, and affecting immune-associated signalling pathways associated with 3CL hydrolase and ACE2 (Hong et al., 2020).

### 6.6 Coronil

Coronil is an ayurvedic triherbal formulation, that is clinically used as an immunomodulator in patients with COVID-19. Coronil contains extracts from *Withania somnifera* (L.) Dunal, *Tinospora cordifolia* (Willd.) Hook. f. and Thomson, and *Ocimum tenuiflorum* L (Balkrishna et al., 2021a). Balkrishna *et al.*, reported the anti-SARS-CoV-2 activity of coronil using the zebrafish model. They found that coronil potentially inhibited SARS-CoV-2 spike protein, and reducing the behavioural fever. Coronil also attenuates and modulates the cytokines production viz. IL-6 and TNF-alpha when tested in A549 cell lines (Balkrishna et al., 2020). Balkrishna *et al.*, also reported the ACE-2 inhibitory potential of coronil (Balkrishna et al., 2021a). In a cross-sectional satisfaction covid survey, which Balkrishna et al., had conducted on 367 patients participants, they found treatment satisfaction in patients when using Divya-Swasari-Coronil-Kit (Balkrishna et al., 2021b).

### 6.7 Kabasura Kudineer

KSK is a polyherbal formulation of India’s Siddha System of Medicine, well known to be traditionally used in diseases similar to that of COVID-19. Natarajan *et al.*, had conducted a single centre, randomized controlled trial in Chennai, India on RT-PCR confirmed COVID-19 cases. Their trial results suggested that KSK could significantly reduce the viral load of SARS-CoV-2 in patients, and did not report any clinically diagnosed, serious adverse effect (Natarajan et al., 2021).

### 6.8 W*ithania somnifera* (L.) Dunal

*Withania somnifera* (L.) Dunal, commonly known as ashwagandha, is a well-known medicinal plant having multiple therapeutic effects. Chopra *et al.*, had conducted a randomized, multicentre study on 400 participants to assess the efficacy and safety when using ashwagandha in place of hydroxychloroquine. Their efficacy and safety assessment suggested that ashwagandha has similar effects to hydroxychloroquine, although the therapeutic efficacy of the latter has been heavily criticized until then (Chopra et al., 2021).

### 6.9 Indian Ayurvedic Prescription Medicine Including Coronil (Patanjali Divya Coronil Kit)

Devpura et al., had conducted a placebo controlled randomized double blind trial on 100 COVID-19 patients. The ayurvedic treatment covered different natural products like 1 gm of *Tinospora cordifolia* (Willd.) Hook. f. and Thomson, 2 gm of Swasari Ras which is a traditional herbo-mineral formulation, 0.5 gm of *Withania somnifera* (L.) Dunal, and 0.5 g of *Ocimum tenuiflorum* L., along with a traditional nasal drop, Anu Taila. *Tinospora cordifolia* (Willd.) Hook. f. and Thomson, *Withania somnifera* (L.), and *Ocimum tenuiflorum* L. were combined in the form of a 500 mg tablet, Coronil. With 71% recovery on Day 3 and 100% recovery on day 7 when treated with this Patanjali Divya Coronil Kit, in comparison to 60% recovery in placebo group. On day 7, significant fold change reduction was also marked when checked for serum levels of hs-CRP, IL-6 and TNF-alpha in comparison to placebo group, with no clinically observed adverse effects (Devpura et al., 2021).

### 6.10 Persian Medicine Herbal Formulations

Karimi and the team had performed multicenter, randomized and controlled clinical trial on 358 COVID-19 patients in Iran, to assess the potential of three herbal formulations based on Persian Medicine System. The treatment consists of two herbal capsules and one herbal decoction, where capsule 1 contains extracts prepared from the root of *Rheum palmatum* L., rhizome of *Glycyrrhiza glabra* L., and fruit peel of *Punica granatum* L.; capsule 2 contains seeds of *Nigella sativa* L. in powdered form; while herbal decoction contains powdered herbs of “*Matricaria chamomilla* L., *Zataria multiflora* Boiss., *G. glabra* L., *Ziziphus jujuba* Mill., *Ficus carica* L., *Urtica dioica* L., *Althaea officinalis* L., and *Nepeta bracteata* Benth.”. 174 patients received standard treatment as per the government protocols, while 184 received these herbal remedies along with standard treatment for a period of 7 days. The results clearly suggested that the combination of herbal remedies along with standard treatment has not accelerated the clinical improvement and decrease in symptoms, but it has also significantly reduced the hospital stay duration. Further, patients have well accepted the herbal treatment (Karimi et al., 2021).

## 7 Non-Validated Candidates Based on Hypothesis or Earlier Antiviral Knowledge

Going through the literature, it has been witnessed that there are many molecules and formulations which were hypothesized for their potential to combat COVID-19 based on their antiviral activities reported earlier against SARS-CoV or MERS-CoV or any other virus. We have covered that information in Table 2.

**TABLE 2:** Non-validated candidates based on hypothesis or earlier antiviral knowledge.

Classification	Natural product	Function	Virus	Refs
Polyphenols	Resveratrol (Figure 8A)	Inhibit the replication *in vitro*	MERS-CoV	Lin et al. (2017), Marinella (2020)
		Inhibit intracellular viral multiplication *in vitro,* decrease the death rate in piglets	Pseudorabies virus	Zhao et al. (2017b), Marinella (2020)
		Downregulate TNF-alpha levels and diminish diarrhea in piglets	Rotavirus	Cui et al. (2018), Marinella (2020)
	Tetrahydrocurcumin (Figure 8B)	Decrease the nucleoprotein expression, prevent the influenza virus infection	H1N1	Richart et al. (2018), Lai et al. (2020b), Gupta et al. (2020)
	Monoacetylcurcumin (Figure 8C)	Prevent the influenza virus infection	Influenza virus	Richart et al. (2018), Gupta et al. (2020)
Alkaloids	Homoharringtonine (Figure 8D)	Powerful antiviral activity	Herpes virus	Dong et al. (2018), Kim and Song (2019), Hassan (2020)
	Emetine (Figure 8E)	Anti-herpes	Herpes virus	Mukhopadhyay et al. (2016), Khandelwal et al. (2017), Andersen et al. (2019), Hassan (2020)
	Lycorine (Figure 8F)	Prevent the transport of nucleoprotein	Influenza virus	He et al. (2013), Zhang et al. (2020a)
		Prevent the autophagy or RNA translation	EV71	Liu et al. (2011a), Wang et al. (2019), Zhang et al. (2020a)
	Reserpine (Figure 8G)	Anti-SARS activities	SARS-Cov	Prasad et al. (2020)
	Tetrandrine (Figure 8H)	Protect the host infected through the viral transmission by inhibiting endo-lysosomal Two-Pore Channels (TPCs)	Ebola virus	Sakurai et al. (2015), Filippini et al. (2020)
Terpenoid	Artemisinin (Figure 8I)	Prevent the bioactive chymotrypsin-like protease and replication of the virus	SARS-Cov	Li et al. (2005), Law et al. (2020)
Flavonoids	Epigallocatechin-3-Gallate (Figure 8J)	Upregulate the Nrf2 expression which could relieve oxidative stress and inflammation, reduce the ACE2 and increase the expression of antiviral genes (RIG-I, IFN-β, and MxA)	Influenza A virus	Kesic et al. (2011), Mendonca and Soliman (2020)
	Naringenin (Figure 8K)	Decrease secretion of the virus	Hepatitis C virus	Nahmias et al. (2008), Filippini et al. (2020)
		Inhibit replication and infection	influenza A virus	Dong et al. (2014), Filippini et al. (2020)
			dengue virus	Frabasile et al. (2017), Filippini et al. (2020)
			Zika virus	Cataneo et al. (2019), Filippini et al. (2020)
Polyketides	Emodin (Figure 8L)	Interdict the binding of the S protein to ACE2, prevent the infection	SARS-Cov	Ho et al. (2007), Prasad et al. (2020)
Glycosides	Saikosaponins	Prevent the penetration and adsorption of the virus	HCoV-229E	Cheng et al. (2006a), Prasad et al. (2020)
	Aescin (Figure 8M)	Anti-SARS activities	SARS-Cov	Prasad et al. (2020)
Carotenoids	Astaxanthin (Figure 8N)	Janus kinase/signal transducer and activator of transcription; antiapoptotic agent	Not checked	Fakhri et al. (2020)
Mixture/Crude	Turmeric	Increase the expression of TNF-α and the IFN-β mRNA	H5N1	Richart et al. (2018), Gupta et al. (2020)
	Sumac extract	Inhibit reverse transcriptase and protease	HIV-1	Kadokura et al. (2015), Korkmaz (2020)
		Prevent the process of attachment and penetration	HSV	Reichling et al. (2009), Korkmaz (2020)
	*Toona sinensis* (Juss.) M.Roem. tender leaf extract	Prevent the replication of the virus	SARS-Cov	Chen et al. (2008), Prasad et al. (2020)
	Tylophorine compounds	Prevent the replication of TGEV which induce apoptosis and cytopathic effect	TGEV	Yang et al. (2010), Prasad et al. (2020)
		Relieve cytopathic effect	SARS-Cov	Yang et al. (2010), Prasad et al. (2020)
	*Euphorbia neriifolia* L. leaves ethanolic extracts	Increase the survival of infected cells	HCoV-229E	Chang et al. (2012), Prasad et al. (2020)
Proteins/Amino acids/Peptides	Mannose-binding lectins	Prevent the replication of the virus	SARS-Cov	Keyaerts et al. (2007), Prasad et al. (2020)
	Tetra-O-galloyl-β-D-glucose	Defense of the virus entry	SARS-Cov	Yi et al. (2004), Prasad et al. (2020)
	Cinanserin	Inhibit the activity of the main protease	SARS-Cov	Gao et al. (2003), Di Micco et al. (2020)
		Prevent the replication of the virus	HCoV-229E	

**FIGURE 8:**
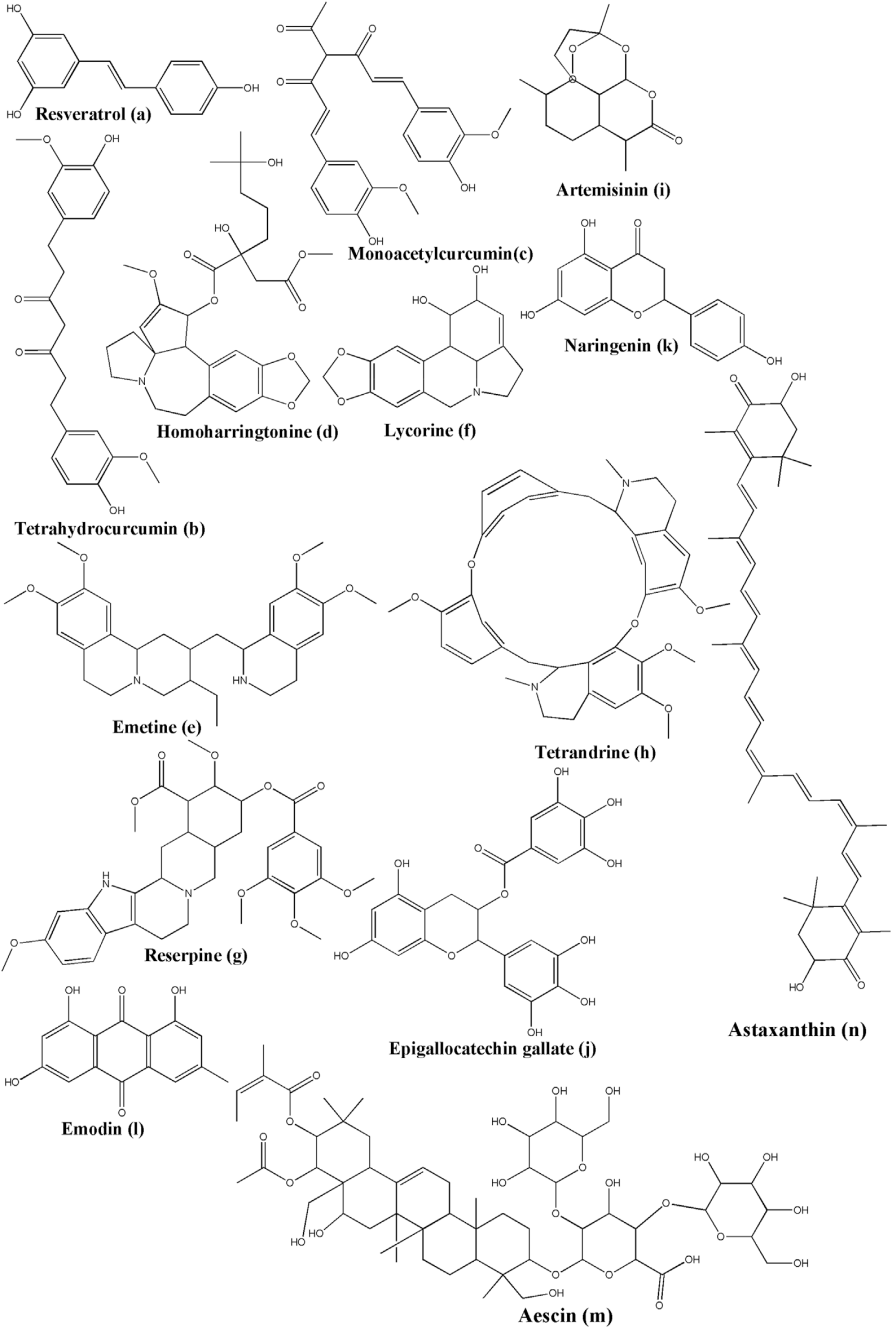
Non-validated candidates based on hypothesis or earlier antiviral knowledge.

## 8.Conclusion, Limitations, and Future Perspectives

SARS-CoV, SARS-CoV-2, and MERS-CoV have been associated with betaCoVs. SARS-CoV, and MERS-CoV were controlled due to lesser geographical spreading, however SARS-CoV-2 has spread throughout the world. The transmission of SARS-CoV-2 as shown in Figure 1 clearly reflects the importance of hygiene and sanitation, utilization of mask, physical distancing and limitation of large-scale gatherings. The authors have further elaborated on the role of intestinal microbiota and pro-inflammatory biomarkers in the prognosis, diagnosis and treatment of COVID-19 disease. Gut microbiota is a multimodal entity with an established involvement in inflammation, immunity and drug metabolism. Pro-inflammatory markers associated with intestinal microbiota are interleukin 1b, interleukin 8, interleukin 10, interleukin 12, TNF, and interferon type 1.

Vaccines have greatly contributed to the prevention of COVID-19 since December 2020. Nevertheless, a number of vaccinated individuals, predominantly those with severe comorbidities or immune compromise remain vulnerable to severe infection, hospitalization and death. Moreover, the duration of immunity remains debatable and can be undermined by novel SARS-CoV-2 strains (Dolgin, 2021). Thus, exploring additional therapeutic solutions, including those derived from medicinal plants remains relevant.

**FIGURE 9:**
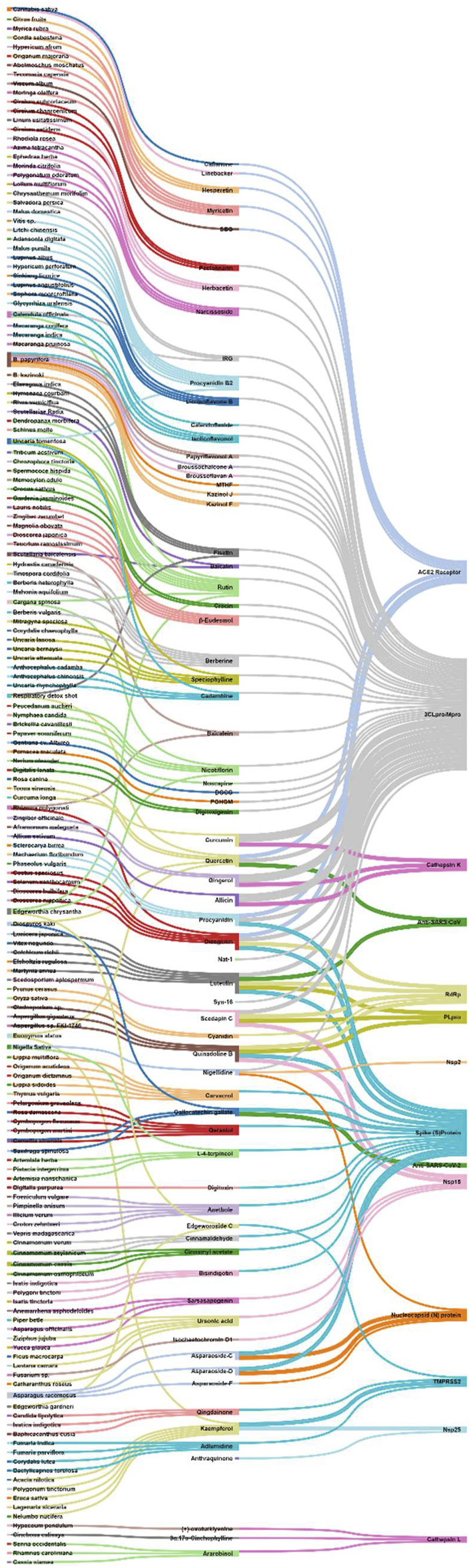
Interactive analysis map between biological sources, natural secondary metabolites, and targets to combat COVID-19.

**FIGURE 10:**
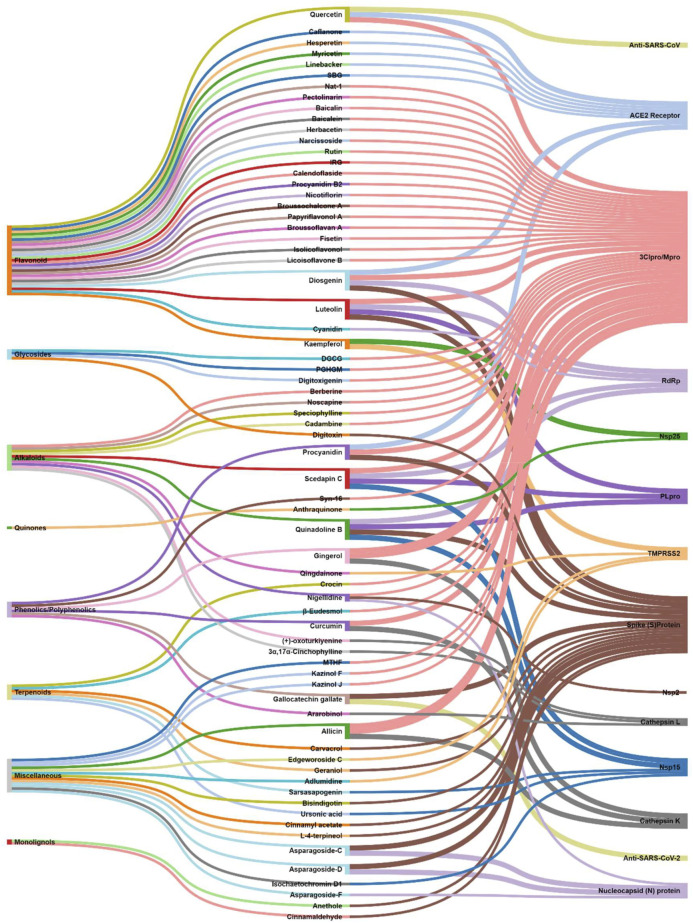
Interactive analysis map between phytochemical classes, natural secondary metabolites, and targets to combat COVID-19.

It is pertinent to note that so far, the efficacy of numerous natural products against the principal COVID-19 therapeutic targets, namely NSP25, ACE2 receptor, 3CL pro/Mpro, RdRp, PL Pro, TMPRSS2, Cathepsin L, Nsp2, Spike (s) protein, Nsp15, and nucleocapsid (N) protein, has been investigated. The authors have covered 70 natural products which were broadly distributed in 165 biological sources. They were active against various targets for combating COVID-19 (Refer to Figure 9). In regard to the covered literature, we found it very interesting that few compounds have the potential to act multi-dimensionally against COVID-19, such as quercetin, diosgenin, scedapin C, luteolin, gallocatechin gallate, quinadoline B, procyanidin, curcumin, gingerol, allicin, kaempferol, nigellidine, asparagoside-C, and asparagoside-D. An interactive analysis map of different phytochemical classes is linked to those natural products which have a documented potential against SARS-CoV-2 (Figure 10). It has been observed that the majority of the studied molecules belongs to the flavonoid, alkaloid and terpenoids category. ACE-2 inhibitory potential was most recorded in compounds bearing flavonoid moiety, which probably suggests the involvement of flavonoid scaffold in interacting with ACE-2 amino acid residues. Multitarget molecules are mostly the ones having phenolic moiety. As per the covered literature, all the terpenoids and monolignols were reported with a single target potential.

The multitarget potential (also known as polypharmacology) of these natural products can become the basis of regimens covering different strains of the virus. This can be further illustrated with a number of examples:

• As mentioned before, the entry of SARS-CoV-2 in the host cell was regulated by the spike protein (S-glycoprotein) of the virus and ACE-2 receptor of the host cell (Yang J. et al., 2020). For instance, diosgenin, a dual acting compound, has the tendency to bind with both, ACE-2 receptor as well as Spike(S) protein.
• 3CLpro (also known as Mpro) and PLpro are the key protease enzymes which are responsible for the replication of SARS-CoV-2 and for virus spread (Shin et al., 2020; Tahir ul Qamar et al., 2020; Mody et al., 2021). Quercetin has shown potential to bind ACE2 receptor as well as 3CLpro. Quercetin can thus inhibit the replication of SARS-CoV-2, as well as stop the entry of this virus in the host cell. On top of this, procyanidin, a flavonoid, is capable of binding with the spike(S)-protein, ACE-2 receptor, and 3Clpro.
• RdRp is an important RNA polymerase involved in viral replication. As a matter of fact, it is a target of remdesivir (Jiang et al., 2021). Luteolin, a compound found in edible plants, has a multitarget potential to bind with 3CLpro, PLpro, RdRp, and Spike(S) protein.
• TMPRSS2 is an additional important target from the host cell side, as it is responsible for spike(S) protein priming and activation, thus responsible for SARS-CoV-2 pathogenicity (Hoffmann et al., 2020; Mollica et al., 2020). This makes compounds like kaempferol and adlumidine as important because of their binding potential to TMPRSS2.
• Similarly to TMPRSS2, cathepsin K/L also plays important role in the activation of spike(S) protein. Hence, compounds targeting cathepsin L like allicin, gingerol, curcumin are having promising potential to aid in circumventing the pathogenicity of SARS-CoV-2.
• Nucelocapsid(N) protein in SARS-CoV-2 is a key structural RNA-binding protein, which plays pivotal role in virus transcription and assembly (McBride et al., 2014; Cubuk et al., 2021). This indicates the importance of compounds like nigellidine, Asparagoside-C, Asparagoside-D, and Asparagoside-F, who can bind to this protein.

The majority of the results discussed in this article derived from *in silico* studies. The role of computational tools in drug discovery, especially against viral infections has been frequently highlighted during the pandemic (Matter and Sotriffer, 2011; Phillips et al., 2018). The *in silico* research of Tao and colleagues (2020), serves as an example indicating the potential of baicalin against SARS-CoV-2 (Tao Q. et al., 2020). On these grounds, Zandi and colleagues (2021) have experimentally yielded that baicalin can have comparable results with remdesivir against COVID-19 (Zandi et al., 2021). Keeping the potential of computational studies in mind, we strongly recommend to researchers to experimentally assess the drug potential of thes listed natural products, either alone or in combination with other natural compounds or in combination with other standard antiviral drugs (Refer to the section: *Natural Products Against SARS-CoV-2: Computational to Preclinical Studies*). The validation of these theoretical studies, may lead to a potent anti-SARS-CoV-2 agent.

As mentioned before, the retrospective search for the sources of the reported natural products, indicated that some plants possess multiple bioactive components which could act simultaneously against various COVID-19 therapeutic targets. Some of those sources are *Cannabis sativa* L., respiratory detox shot, *Scutellaria baicalensis* Georgi, *Uncaria tomentosa* (Willd. ex Schult.) DC., *Polygonatum sibiricum* Redouté, *Diospyros kaki* L. f., *Euonymus alatus* (Thunb.) Siebold, *Camellia sinensis* (L.) Kuntze, *Cinnamomum verum* J. Presl, *Caragana spinosa* (L.) Hornem., *Edgeworthia chrysantha* Lindl., *Nigella sativa* L., *Broussonetia papyrifera* (L.) L'Hér. ex Vent., *Calendula officinalis* L., and *Asparagus racemosus* Willd. Some bioactive compounds with anti-SARS-CoV-2 potential are very common and reportedly being found in multiple sources, namely hesperetin, myricetin, pectolinarin, herbacetin, narcissoside, baicalin, procyanidin B2, quercetin, and licoisoflavone B. Given the significance of computational data in this COVID-19 pandemic time, to accelerate drug discovery, the authors have discussed these studies in an unbiased manner, acknowledging the need for validation in clinical settings. Perhaps, a polyherbal formulation combining these biological sources could lead to a potent pharmaceutical agent, with relatively low cost of production and presumably high acceptance among populations who are acquainted with these compounds through their traditions. In this context, *Natural Products Against SARS-CoV-2: Computational to Preclinical Studies* has listed 10 polyherbal formulations based on Traditional Chinese Medicine (TCM) Indian Ayurvedic and Siddha Medicine and Persian Medicine. The reported studies included limited number of patients and further clinical investigation is necessary. However, considering that a considerable number of individuals in the aforementioned countries may seek such treatments, being aware of the relevant evidence is important.

**FIGURE 11:**
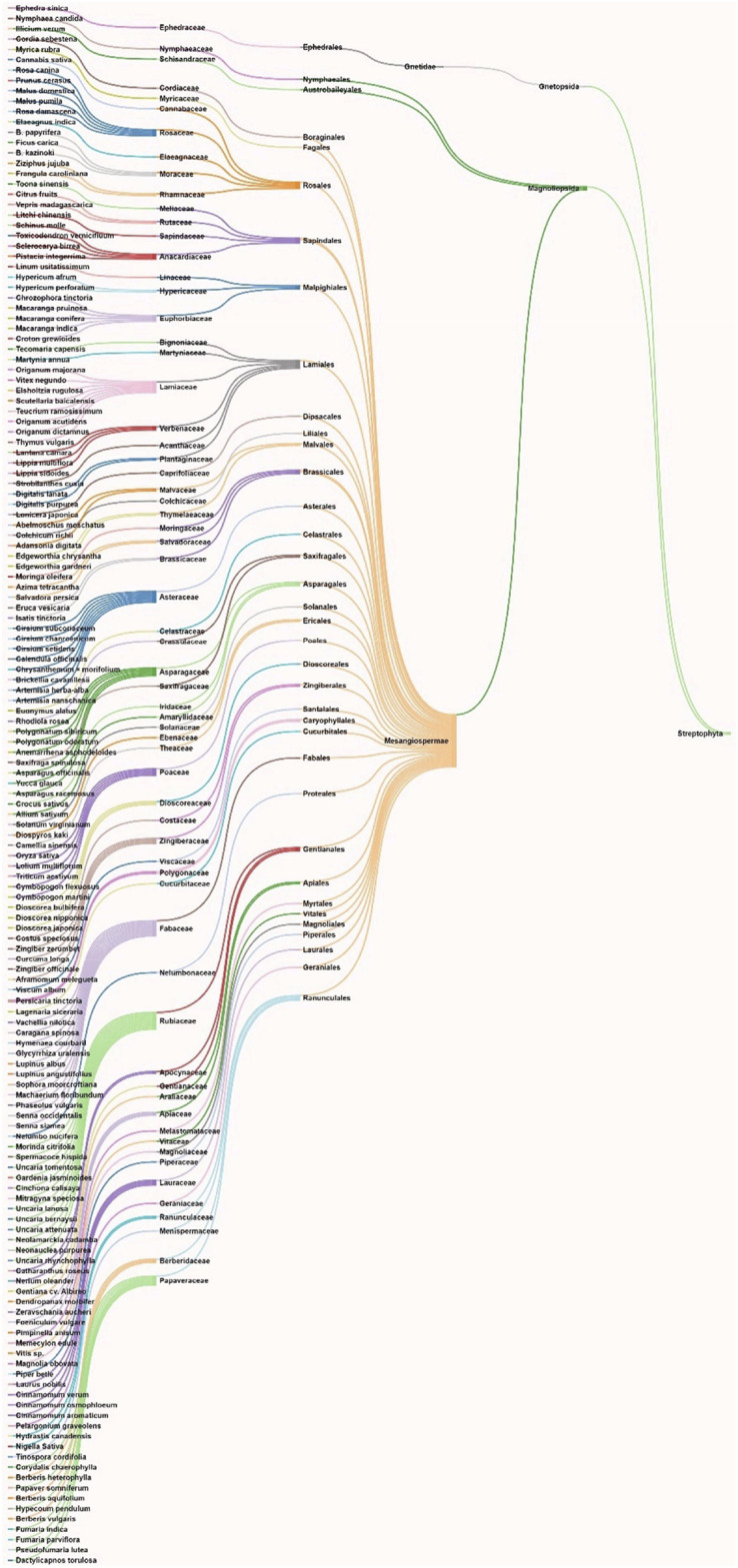
Interactive analysis map biological source-family-order-clade-class-clade.

Interactive analysis matching the covered biological sources with their taxonomical tree was performed in an effort to reveal significant relationships and leverage the insights provided by the present study (Refer Figure 11 as Interactive analysis map biological source-family-order-clade-class-clade). Out of approximately 64 covered families, the families were medicinal plants possessing bioactive compounds to combat COVID-19 were abundant included Rutaceae, Anacardiaceae, Rosaceae, Moraceae, Rhamnaceae, Hypericaceae, Euphorbiaceae, Lamiaceae, Verbenaceae, Plantiginaceae, Salvadoraceae, Brassicaceae, Asteraceae, Poaceae, Asparagaceae, Dioscoreaceae, Fabaceae, Rubiaceae, Apocynaceae, Lauraceae, Berberidaceae, and Papaveraceae. Papaveraceae, Rubiaceae, Fabaceae, Asparagaceae, Poaceae, Asteraceae, Lamiaceae, Euphorbiaceae, and Rosaceae. Similarly, out of approximately 36 covered orders, the most significant ones were Ranunculales, Apiales, Gentianales, Caryophyllales, Zingiberales, Asparagales, Saxifragales, Brassicales, Lamiales, Malphighiales, Rosales, and Sapindales. Ranunculales, Lamiales, Rosales, Sapindales were further the most significant out of all the listed orders. Almost all the covered medicinal plants belong to the clade: Mesangiospermae; class: Magnoliopsida and clade: Streptophyta. Taxonomical classifications were based on similarities and commonalities. Keeping the similarities and commonalities of the taxonomy in mind, we authors recommend to investigate the potential of these families against COVID-19 and its sequelae.

The focus of the present study has been limited to phytochemicals reported between January 2020 and November 2020. This limitation was deemed necessary in order to analyse evidence connecting natural compounds with COVID-19 in a comprehensive manner, as presented in Figure 9–Figure 11. Moreover, the authors limited their search of the biological source of these phytochemicals to the 5–6 more abundant and investigated sources of it. Hence the listing of biological sources is not exhaustive but serves as a guide for future original research.

It is 21 months since the onset of the COVID-19 pandemic, a historical crisis with multilevel implications to health, economy, politics and society. Research related to the management of the disease has culminated in a record number of publications, including a significant amount of work investigating natural compounds in the context of COVID-19. To date, relevant secondary (review) studies have focused either on natural products with known antiviral properties associated with other viral infections (in the sense that some of these properties may render them effective against COVID-19) (Dejani et al., 2021; Ebob et al., 2021) or were not focused on single classes of compounds (da Silva, 2021; Ghidoli et al., 2021; Khazeei Tabari et al., 2021; Kowalczyk et al., 2021; Montenegro-Landivar et al., 2021) or elaborated on synthetic antiviral regimens (Shah et al., 2021). To the authors best knowledge, a review summarizing the evidence about natural products of different classes on COVID-19 has not been published so far. The present study puts this evidence in the context of the etiology and epidemiology of COVID-19, intestinal microbiota and pro-inflammatory markers related to disease, in an effort to outline a comprehensive background for future original research. Overall, this review serves as a guide for research related to the use of natural compounds against SARS-CoV-2. It can be particularly useful to pharmacology researchers in academia and in the industry and may also provide clinical investigators with insights about relevant clinical research. The authors acknowledge the need to enhance this evidence by means of large-scale clinical studies and recognize that currently phytochemicals may be considered as a complement to established treatments and not as a monotherapy. Although studies reporting the use of alternative medicine and ethnomedicine approaches on single or limited numbers of patients have been presented, the authors urge the readers to abide by the best scientific evidence at the time, as described in the guidelines of designated health bodies, and conduct relevant research only after receiving the authorization of ethical committees and the informed consent of the subjects involved.

## 9 Softwares Used

Chemical structures were drawn using ChemDraw Ultra software, while the powerpoint was used for Figures 1, 2. Sankey graph methodology was adopted for making interactive figures like Figures 9‒Figure 11.

## Glossary

alphaCoV: alphacoronavirus
betaCoV: betacoronavirus
deltaCoV: deltacoronavirus
gammaCoV: gammacoronavirus
UVC: Ultraviolet-C
RNA: Ribonucleic Acid
RdRp: RNA-dependent RNA polymerase
3CLpro: 3-chymotrypsin-like protease
PLpro: Papain-like protease
ACE2: Angiotensin-converting enzyme 2
ICUs: Intensive care units
IL6: Interleukin-6
IL1: Interleukin-1
TNF: Tissue Necrosis Factor
CAGs: Cytokine activated genes
Tregs: T regulatory cells
IBDs: Inflammatory bowel diseases
PAMPs: Pathogen associated molecular patterns
MAMPs: Microorganisms associated molecular patterns
PRRs: Pattern recognition receptors
INF-1: Initiation factor-1
PCR: Polymerase chain reaction
S protein: Spike protein
TCM: Traditional Chinese Medicines
LH: Lianhuaqingwen
**IC**_**50**_: 50% Inhibitory concentration
anti- HCV: Anti-Hepatitis C Virus
SBG: (+)-Syringaresinol-O-beta-D-glucoside
NSP: Non-structural polypeptide
TMPRSS2: Transmembrane protease, serine 2
qPCR: Quantitative PCR
Da: Dalton
M pro: Main protease
IRG: Isorhamnetin-3-O-b-D-glucoside
anti-HSV: Anti-Herpex simplex virus
Anti-HIV: Anti-Human immunodeficiency virus
MD: Molecular dynamics
RMSD: Root mean square deviation
RMSF: Root mean square fluctuation
VDW: van der Waals
ADMET: Absorption, distribution, metabolism, excretion, and toxicity
GI: Gastrointestinal
H-bond: Hydrogen bond
N protein: Nucleocapsid protein
DGCG: Delphinidin 3,3′-di-glucoside-5-(6-*p*-coumarylglucoside)
PGHGM: Pelargonidin 3-O-[β-D-Glucopyranosyl-(1->2)-[4-hydroxycinnamoyl-(->6)]-β-D-glucopyranoside](E-) 5-O-(6-O-malonyl-β-D-glucopyranoside)
AIDS: Acquired immune deficiency syndrome
HIV: Human immunodeficiency virus
TCMSP: Traditional Chinese Medicine Systems Pharmacology
MTHF: 3-(3-Methylbut-2-enyl)-3,4,7-trihydroxyflavane
LHQW: Lianhuaqingwen
PLD: Pudilan
CT: Computed tomography
IgG: Immunoglobulin G
IgM: Immunoglobulin M
QFDY: Qing-Fei-Da-Yuan
TPCs: Two-Pore Channels
TGEV: Transmissible gastroenteritis virus.

## References

[B1] Aanouz I. Belhassan A. El-Khatabi K. Lakhlifi T. El-Ldrissi M. Bouachrine M. (2020). Moroccan Medicinal Plants as Inhibitors against SARS-CoV-2 Main Protease: Computational Investigations. J. Biomol. Struct. Dyn. 39, 2971–2979. 10.1080/07391102.2020.1758790 32306860 PMC7212546

[B2] Abdalla S. Zarga M. A. Sabri S. (1994). Effects of the Flavone Luteolin, Isolated fromColchicum Richii, on guinea-pig Isolated Smooth Muscle and Heart and on Blood Pressure and Blood Flow. Phytother. Res. 8 (5), 265–270. 10.1002/ptr.2650080503

[B3] Abdel-Naim A. B. Alghamdi A. A. Algandaby M. M. Al-Abbasi F. A. Al-Abd A. M. Eid B. G. (2018). Rutin Isolated from *Chrozophora tinctoria* Enhances Bone Cell Proliferation and Ossification Markers. Oxid Med. Cel Longev 2018, 5106469. 10.1155/2018/5106469 PMC5831974 29636845

[B4] Abraham J. P. Plourde B. D. Cheng L. (2020). Using Heat to Kill SARS-CoV-2. Rev. Med. Virol. 30 (5), e2115. 10.1002/rmv.2115 32614102 PMC7361064

[B5] Al-Bayati F. A. Mohammed M. J. (2009). Isolation, Identification, and Purification of Cinnamaldehyde fromCinnamomum Zeylanicumbark Oil. An Antibacterial Study. Pharm. Biol. 47 (1), 61–66. 10.1080/13880200802430607

[B6] Ali A. Assaf M. El-Shanawany M. Kamel M. (1997). Flavonoid Glycosides from the Leaves of Salvadora Persica L. Bull. Pharm. Sci. Assiut 20 (2), 181–186. 10.21608/bfsa.1997.68759

[B7] Anand S. Mande S. S. (2018). Diet, Microbiota and Gut-Lung Connection. Front. Microbiol. 9, 2147. 10.3389/fmicb.2018.02147 30283410 PMC6156521

[B8] Andersen P. I. Krpina K. Ianevski A. Shtaida N. Jo E. Yang J. (2019). Novel Antiviral Activities of Obatoclax, Emetine, Niclosamide, Brequinar, and Homoharringtonine. Viruses 11 (10), 964. 10.3390/v11100964 PMC6832696 31635418

[B9] Anderson A. M. Mitchell M. S. Mohan R. S. (2000). Isolation of Curcumin from Turmeric. J. Chem. Educ. 77 (3), 359. 10.1021/ed077p359

[B10] Atalan A. (2020). Erratum to "Is the Lockdown Important to Prevent the COVID-19 Pandemic? Effects on Psychology, Environment and Economy-Perspective" [Ann. Med. Surg. 56 (2020) 38-42]. Ann. Med. Surg. (Lond) 56, 217–242. 10.1016/j.amsu.2020.07.001 32562476 PMC7293850

[B11] Avila-Villarreal G. Aguilar-Guadarrama B. Castillo-España P. González-Trujano M. Villalobos Azucena Ibeth C. Estrada-Soto S. (2016). Anxiolytic-like Effects of Brickellia Cavanillesii and Their Bioactive Compounds Nicotiflorin and Acacetin in Experimental Models in Mice. Planta Med. 81 (S 01), S1–S381. 10.1055/s-0036-1596396

[B12] Baek Y. S. Ryu Y. B. Curtis-Long M. J. Ha T. J. Rengasamy R. Yang M. S. (2009). Tyrosinase Inhibitory Effects of 1,3-diphenylpropanes from Broussonetia Kazinoki. Bioorg. Med. Chem. 17 (1), 35–41. 10.1016/j.bmc.2008.11.022 19046886

[B13] Balkrishna A. Haldar S. Singh H. Roy P. Varshney A. (2021a). Coronil, a Tri-herbal Formulation, Attenuates Spike-Protein-Mediated SARS-CoV-2 Viral Entry into Human Alveolar Epithelial Cells and Pro-inflammatory Cytokines Production by Inhibiting Spike Protein-ACE-2 Interaction. J. Inflamm. Res. 14, 869–884. 10.2147/JIR.S298242 33758527 PMC7981146

[B14] Balkrishna A. Raj P. Singh P. Varshney A. (2021b). Influence of Patient-Reported Treatment Satisfaction on Psychological Health and Quality of Life Among Patients Receiving Divya-Swasari-Coronil-Kit against COVID-19: Findings from a Cross-Sectional "SATISFACTION COVID" Survey. Patient Prefer Adherence 15, 899–909. 10.2147/PPA.S302957 33958858 PMC8096451

[B15] Balkrishna A. Solleti S. K. Verma S. Varshney A. (2020). Application of Humanized Zebrafish Model in the Suppression of SARS-CoV-2 Spike Protein Induced Pathology by Tri-herbal Medicine Coronil via Cytokine Modulation. Molecules 25 (21), 5091. 10.3390/molecules25215091 PMC7662214 33147850

[B16] Bao W. Pan H. Lu M. Ni Y. Zhang R. Gong X. (2007). The Apoptotic Effect of Sarsasapogenin from Anemarrhena Asphodeloides on HepG2 Human Hepatoma Cells. Cell Biol Int 31 (9), 887–892. 10.1016/j.cellbi.2007.02.001 17400003

[B17] Basha S. A. Mishra R. K. Jha R. N. Pandey V. B. Singh U. P. (2002). Effect of Berberine and (+/-)-bicuculline Isolated from Corydalis Chaerophylla on Spore Germination of Some Fungi. Folia Microbiol. (Praha) 47 (2), 161–165. 10.1007/bf02817675 12058395

[B18] Beckett A. H. Shellard E. J. Phillipson J. D. Lee C. M. (1965). Alkaloids from Mitragyna Speciosa (Korth.). J. Pharm. Pharmacol. 17 (11), 753–755. 10.1111/j.2042-7158.1965.tb07599.x 4379809

[B19] Begum S. Raza S. M. Siddiqui B. S. Siddiqui S. (2004). Triterpenoids from the Aerial Parts of Lantana Camara. J. Nat. Prod. 58 (10), 1570–1574. 10.1021/np50124a014

[B20] Ben Sghaier M. Mousslim M. Pagano A. Ammari Y. Luis J. Kovacic H. (2016). β-Eudesmol, a Sesquiterpene from Teucrium Ramosissimum, Inhibits Superoxide Production, Proliferation, Adhesion and Migration of Human Tumor Cell. Environ. Toxicol. Pharmacol. 46, 227–233. 10.1016/j.etap.2016.07.019 27497729

[B21] Bernstein C. N. (2014). “Antibiotics, Probiotics and Prebiotics in IBD,” in Nutrition, Gut Microbiota and Immunity (New York: Therapeutic Targets for IBD.), 83–100. 10.1159/000360713 25227297

[B22] Berretta A. A. Silveira M. A. D. Cóndor Capcha J. M. De Jong D. (2020). Propolis and its Potential against SARS-CoV-2 Infection Mechanisms and COVID-19 Disease: Running Title: Propolis against SARS-CoV-2 Infection and COVID-19. Biomed. Pharmacother. 131, 110622. 10.1016/j.biopha.2020.110622 32890967 PMC7430291

[B23] Blaskó G. Hussain S. F. Shamma M. (2004). (-)-Corlumine, A New Phthalideisoquinoline Alkaloid from Fumaria Parviflora. J. Nat. Prod. 44 (4), 475–477. 10.1021/np50016a014

[B24] Boyer J. Liu R. H. (2004). Apple Phytochemicals and Their Health Benefits. Nutr. J. 3 (1), 5. 10.1186/1475-2891-3-5 15140261 PMC442131

[B25] Buitrago-Garcia D. Egli-Gany D. Counotte M. J. Hossmann S. Imeri H. Ipekci A. M. (2020). Occurrence and Transmission Potential of Asymptomatic and Presymptomatic SARS-CoV-2 Infections: A Living Systematic Review and Meta-Analysis. Plos Med. 17 (9), e1003346. 10.1371/journal.pmed.1003346 32960881 PMC7508369

[B26] Butterweck V. Petereit F. Winterhoff H. Nahrstedt A. (1998). Solubilized Hypericin and Pseudohypericin from *Hypericum perforatum* Exert Antidepressant Activity in the Forced Swimming Test. Planta Med. 64 (4), 291–294. 10.1055/s-2006-957437 9619107

[B27] Caspi E. Hornby G. M. (1968). Biosynthesis of Plant Sterols-III. Mechanism of Saturation of Ring B in Pregnenolone during its Conversion to Digitoxigenin in Digitalis Lanata. Phytochemistry 7 (3), 423–427. 10.1016/s0031-9422(00)90882-3

[B28] Cataneo A. H. D. Kuczera D. Koishi A. C. Zanluca C. Silveira G. F. Arruda T. B. (2019). The Citrus Flavonoid Naringenin Impairs the *In Vitro* Infection of Human Cells by Zika Virus. Sci. Rep. 9 (1), 16348. 10.1038/s41598-019-52626-3 31705028 PMC6841724

[B29] Čerňáková M. Košťálová D. (2008). Antimicrobial Activity of Berberine-A Constituent ofMahonia Aquifolium. Folia Microbiol. 47 (4), 375–378. 10.1007/bf02818693 12422513

[B30] Chan J. F. To K. K. Tse H. Jin D. Y. Yuen K. Y. (2013). Interspecies Transmission and Emergence of Novel Viruses: Lessons from Bats and Birds. Trends Microbiol. 21 (10), 544–555. 10.1016/j.tim.2013.05.005 23770275 PMC7126491

[B31] Chang F. R. Yen C. T. Ei-Shazly M. Lin W. H. Yen M. H. Lin K. H. (2012). Anti-human Coronavirus (Anti-HCoV) Triterpenoids from the Leaves of Euphorbia Neriifolia. Nat. Prod. Commun. 7 (11), 1415–1417. 10.1177/1934578x1200701103 23285797

[B32] Chen C. J. Michaelis M. Hsu H. K. Tsai C. C. Yang K. D. Wu Y. C. (2008). Toona Sinensis Roem Tender Leaf Extract Inhibits SARS Coronavirus Replication. J. Ethnopharmacol 120 (1), 108–111. 10.1016/j.jep.2008.07.048 18762235 PMC7127248

[B33] Chen J. J. Wang S. W. Chiang Y. R. Pang K. L. Kuo Y. H. Shih T. Y. (2020a). Highly Oxygenated Constituents from a Marine Alga-Derived Fungus Aspergillus giganteus NTU967. Mar. Drugs 18 (6), 303. 10.3390/md18060303 PMC7374281 32517237

[B34] Chen L. Deng H. Cui H. Fang J. Zuo Z. Deng J. (2017). Inflammatory Responses and Inflammation-Associated Diseases in Organs. Oncotarget 9 (6), 7204–7218. 10.18632/oncotarget.23208 29467962 PMC5805548

[B35] Chen L. Liu W. Zhang Q. Xu K. Ye G. Wu W. (2020b). RNA Based mNGS Approach Identifies a Novel Human Coronavirus from Two Individual Pneumonia Cases in 2019 Wuhan Outbreak. Emerg. Microbes Infect. 9 (1), 313–319. 10.1080/22221751.2020.1725399 32020836 PMC7033720

[B36] Chen N. Zhou M. Dong X. Qu J. Gong F. Han Y. (2020c). Epidemiological and Clinical Characteristics of 99 Cases of 2019 Novel Coronavirus Pneumonia in Wuhan, China: a Descriptive Study. Lancet 395 (10223), 507–513. 10.1016/s0140-6736(20)30211-7 32007143 PMC7135076

[B37] Cheng P. W. Ng L. T. Chiang L. C. Lin C. C. (2006a). Antiviral Effects of Saikosaponins on Human Coronavirus 229E *In Vitro* . Clin. Exp. Pharmacol. Physiol. 33 (7), 612–616. 10.1111/j.1440-1681.2006.04415.x 16789928 PMC7162031

[B38] Cheng S. S. Liu J. Y. Hsui Y. R. Chang S. T. (2006b). Chemical Polymorphism and Antifungal Activity of Essential Oils from Leaves of Different Provenances of Indigenous Cinnamon (Cinnamomum Osmophloeum). Bioresour. Technol. 97 (2), 306–312. 10.1016/j.biortech.2005.02.030 16171686

[B39] Chiang Y. M. Chang J. Y. Kuo C. C. Chang C. Y. Kuo Y. H. (2005). Cytotoxic Triterpenes from the Aerial Roots of Ficus Microcarpa. Phytochemistry 66 (4), 495–501. 10.1016/j.phytochem.2004.12.026 15694457

[B40] ChikhaleR. V.SinhaS. K.PatilR. B.PrasadS. K.ShakyaA.GuravN. (2020). In-silico Investigation of Phytochemicals from *Asparagus racemosus* as Plausible Antiviral Agent in COVID-19. J. Biomol. Struct. Dyn. 39, 5033–5047. 10.1080/07391102.2020.1784289 32579064

[B41] Cho Y.-H. Kim N.-H. Khan I. Yu J. M. Jung H. G. Kim H. H. (2016). Anti-inflammatory Potential of Quercetin-3-O-β-D-(“2”-Galloyl)-Glucopyranoside and Quercetin Isolated from Diospyros Kaki Calyx via Suppression of MAP Signaling Molecules in LPS-Induced RAW 264.7 Macrophages. J. Food Sci. 81 (10), C2447–C2456. 10.1111/1750-3841.13497 27648736

[B42] Choi J. Lee K. T. Ka H. Jung W. T. Jung H. J. Park H. J. (2001). Constituents of the Essential Oil of the Cinnamomum cassia Stem Bark and the Biological Properties. Arch. Pharm. Res. 24 (5), 418–423. 10.1007/bf02975187 11693543

[B43] Choi J. H. Kim D. W. Park S. E. Lee H. J. Kim K. M. Kim K. J. (2015). Anti-thrombotic Effect of Rutin Isolated from Dendropanax Morbifera Leveille. J. Biosci. Bioeng. 120 (2), 181–186. 10.1016/j.jbiosc.2014.12.012 25777266

[B44] Chopra A. Srikanth N. Patwardhan B. Group A. C. R. (2021). Withania Somnifera as a Safer Option to Hydroxychloroquine in the Chemoprophylaxis of COVID-19: Results of Interim Analysis. Complement. Ther. Med. 62, 102768. 10.1016/j.ctim.2021.102768 34418550 PMC8372474

[B45] Chowdhury P. (2020). In Silico investigation of Phytoconstituents from Indian Medicinal Herb ‘Tinospora Cordifolia (Giloy)' against SARS-CoV-2 (COVID-19) by Molecular Dynamics Approach. J. Biomol. Struct. Dyn. 39, 1–18. 10.1080/07391102.2020.1803968 PMC7484574 32762511

[B46] Ciavarella C. Motta I. Valente S. Pasquinelli G. (2020). Pharmacological (Or Synthetic) and Nutritional Agonists of PPAR-γ as Candidates for Cytokine Storm Modulation in COVID-19 Disease. Molecules 25 (9), 2076. 10.3390/molecules25092076 PMC7248959 32365556

[B47] Cubuk J. Alston J. J. Incicco J. J. Singh S. Stuchell-Brereton M. D. Ward M. D. (2021). The SARS-CoV-2 Nucleocapsid Protein Is Dynamic, Disordered, and Phase Separates with RNA. Nat. Commun. 12 (1), 1936. 10.1038/s41467-021-21953-3 33782395 PMC8007728

[B48] Cui Q. Fu Q. Zhao X. Song X. Yu J. Yang Y. (2018). Protective Effects and Immunomodulation on Piglets Infected with Rotavirus Following Resveratrol Supplementation. PLoS One 13 (2), e0192692. 10.1371/journal.pone.0192692 29466421 PMC5821335

[B49] da Costa M. P. Bozinis M. C. Andrade W. M. Costa C. R. da Silva A. L. Alves de Oliveira C. M. (2014). Antifungal and Cytotoxicity Activities of the Fresh Xylem Sap of Hymenaea Courbaril L. And its Major Constituent Fisetin. BMC Complement. Altern. Med. 14 (1), 245. 10.1186/1472-6882-14-245 25027026 PMC4223399

[B50] Da J. Xu M. Wang Y. Li W. Lu M. Wang Z. (2019). Kaempferol Promotes Apoptosis while Inhibiting Cell Proliferation via Androgen-dependent Pathway and Suppressing Vasculogenic Mimicry and Invasion in Prostate Cancer. Anal. Cel Pathol (Amst) 2019, 1907698. 10.1155/2019/1907698 PMC6913338 31871879

[B51] da Silva A. P. G. (2021). Fighting Coronaviruses with Natural Polyphenols. Biocatal. Agric. Biotechnol. 37, 102179. 10.1016/j.bcab.2021.102179 34630764 PMC8491928

[B52] da Silva F. M. A. da Silva K. P. A. de Oliveira L. P. M. Costa E. V. Koolen H. H. Pinheiro M. L. B. (2020). Flavonoid Glycosides and Their Putative Human Metabolites as Potential Inhibitors of the SARS-CoV-2 Main Protease (Mpro) and RNA-dependent RNA Polymerase (RdRp). Mem. Inst. Oswaldo Cruz 115, e200207. 10.1590/0074-02760200207 33027419 PMC7534957

[B53] Dang T. -T. T. Facchini P. J. (2012). Characterization of Three O-Methyltransferases Involved in Noscapine Biosynthesis in Opium Poppy. Plant Physiol. 159 (2), 618–631. 10.1104/pp.112.194886 22535422 PMC3375929

[B54] Das P. Majumder R. Mandal M. Basak P. (2020). In-Silico Approach for Identification of Effective and Stable Inhibitors for COVID-19 Main Protease (Mpro) from Flavonoid Based Phytochemical Constituents of Calendula officinalis. J. Biomol. Struct. Dyn. 39, 6265. 10.1080/07391102.2020.1796799 32705952 PMC7441784

[B55] David Phillipson J. Hemingway S. R. (1975). Alkaloids of Uncaria Attenuata, U. Orientalis and U. Canescens. Phytochemistry 14 (8), 1855–1863. 10.1016/0031-9422(75)85310-6

[B56] de Siqueira R. J. Magalhães P. J. Leal-Cardoso J. H. Duarte G. P. Lahlou S. (2006). Cardiovascular Effects of the Essential Oil of Croton Zehntneri Leaves and its Main Constituents, Anethole and Estragole, in Normotensive Conscious Rats. Life Sci. 78 (20), 2365–2372. 10.1016/j.lfs.2005.09.042 16325210

[B57] Dejani N. N. Elshabrawy H. A. Bezerra Filho C. D. S. M. de Sousa D. P. (2021). Anticoronavirus and Immunomodulatory Phenolic Compounds: Opportunities and Pharmacotherapeutic Perspectives. Biomolecules 11 (8), 1254. 10.3390/biom11081254 34439920 PMC8394099

[B58] Devine S. M. Yong C. Amenuvegbe D. Aurelio L. Muthiah D. Pouton C. (2018). Synthesis and Pharmacological Evaluation of Noscapine-Inspired 5-Substituted Tetrahydroisoquinolines as Cytotoxic Agents. J. Med. Chem. 61 (18), 8444–8456. 10.1021/acs.jmedchem.8b00986 30156410

[B59] Devpura G. Tomar B. S. Nathiya D. Sharma A. Bhandari D. Haldar S. (2021). Randomized Placebo-Controlled Pilot Clinical Trial on the Efficacy of Ayurvedic Treatment Regime on COVID-19 Positive Patients. Phytomedicine 84, 153494. 10.1016/j.phymed.2021.153494 33596494 PMC7857981

[B60] Dhand R. Li J. (2020). Coughs and Sneezes: Their Role in Transmission of Respiratory Viral Infections, Including SARS-CoV-2. Am. J. Respir. Crit. Care Med. 202 (5), 651–659. 10.1164/rccm.202004-1263PP 32543913 PMC7462404

[B61] Dhar D. Mohanty A. (2020). Gut Microbiota and Covid-19- Possible Link and Implications. Virus. Res. 285, 198018. 10.1016/j.virusres.2020.198018 32430279 PMC7217790

[B62] Di Micco P. Di Micco G. Russo V. Poggiano M. R. Salzano C. Bosevski M. (2020). Blood Targets of Adjuvant Drugs against COVID19. J. Blood Med. 11, 237–241. 10.2147/JBM.S256121 32694923 PMC7338832

[B63] Di Renzo L. Gualtieri P. Pivari F. Soldati L. Attinà A. Leggeri C. (2020). COVID-19: Is There a Role for Immunonutrition in Obese Patient. J. Transl Med. 18 (1), 415. 10.1186/s12967-020-02594-4 33160363 PMC7647877

[B64] Dickson R. P. (2018). The Lung Microbiome and ARDS. It Is Time to Broaden the Model. Am. J. Respir. Crit. Care Med. 197 (5), 549–551. 10.1164/rccm.201710-2096ED 29091746 PMC6005245

[B65] Dixit S. (2014). Anticancer Effect of Rutin Isolated from the Methanolic Extract of *Triticum aestivum* Straw in Mice. Med. Sci. 2 (4), 153–160. 10.3390/medsci2040153

[B66] Dolgin E. (2021). COVID Vaccine Immunity Is Waning - How Much Does that Matter. Nature 597 (7878), 606–607. 10.1038/d41586-021-02532-4 34548661

[B67] Dong H. J. Wang Z. H. Meng W. Li C. C. Hu Y. X. Zhou L. (2018). The Natural Compound Homoharringtonine Presents Broad Antiviral Activity *In Vitro* and *In Vivo* . Viruses 10 (11), 601. 10.3390/v10110601 PMC6266276 30388805

[B68] Dong W. Wei X. Zhang F. Hao J. Huang F. Zhang C. (2014). A Dual Character of Flavonoids in Influenza A Virus Replication and Spread through Modulating Cell-Autonomous Immunity by MAPK Signaling Pathways. Sci. Rep. 4, 7237. 10.1038/srep07237 25429875 PMC4246350

[B69] Dongare V. Kulkarni C. Kondawar M. Magdum C. Haldavnekar V. Arvindekar A. (2012). Inhibition of Aldose Reductase and Anti-cataract Action of Trans-anethole Isolated from Foeniculum Vulgare Mill. Fruits. Food Chem. 132 (1), 385–390. 10.1016/j.foodchem.2011.11.005 26434305

[B70] Dubey K. Dubey R. (2020). Computation Screening of Narcissoside a Glycosyloxyflavone for Potential Novel Coronavirus 2019 (COVID-19) Inhibitor. Biomed. J. 43 (4), 363–367. 10.1016/j.bj.2020.05.002 32426388 PMC7233213

[B71] Duraipandiyan V. Al-Dhabi N. A. Stephen Irudayaraj S. Sunil C. (2016). Hypolipidemic Activity of Friedelin Isolated from Azima Tetracantha in Hyperlipidemic Rats. Revista Brasileira de Farmacognosia 26 (1), 89–93. 10.1016/j.bjp.2015.07.025

[B72] Ebob O. T. Babiaka S. B. Ntie-Kang F. (2021). Natural Products as Potential Lead Compounds for Drug Discovery against SARS-CoV-2. Nat. Prod. Bioprospect. 1–18. 10.1007/s13659-021-00317-w PMC8435765 34515981

[B73] Eccles R. (2005). Understanding the Symptoms of the Common Cold and Influenza. Lancet Infect. Dis. 5 (11), 718–725. 10.1016/s1473-3099(05)70270-x 16253889 PMC7185637

[B74] Elshamy A. I. Ammar N. M. Hassan H. A. El-Kashak W. A. Al-Rejaie S. S. Abd-ElGawad A. M. (2020). Topical Wound Healing Activity of Myricetin Isolated from Tecomaria Capensis V. Aurea. Molecules 25 (21). 10.3390/molecules25214870 PMC7659475 33105570

[B75] Erenler R. Sen O. Aksit H. Demirtas I. Yaglioglu A. S. Elmastas M. (2016). Isolation and Identification of Chemical Constituents from Origanum Majoranainvestigation of Antiproliferative and Antioxidant Activities. J. Sci. Food Agric. 96 (3), 822–836. 10.1002/jsfa.7155and 25721137

[B76] Fachini-Queiroz F. C. Kummer R. Estevão-Silva C. F. Carvalho M. D. Cunha J. M. Grespan R. (2012). Effects of Thymol and Carvacrol, Constituents of Thymus Vulgaris L. Essential Oil, on the Inflammatory Response. Evid. Based Complement. Alternat Med. 2012, 657026. 10.1155/2012/657026 22919415 PMC3418667

[B77] Fakhar Z. Faramarzi B. Pacifico S. Faramarzi S. (2020). Anthocyanin Derivatives as Potent Inhibitors of SARS-CoV-2 Main Protease: An In-Silico Perspective of Therapeutic Targets against COVID-19 Pandemic. J. Biomol. Struct. Dyn., 1–13. 10.1080/07391102.2020.1801510 32741312

[B78] Fakhri S. Nouri Z. Moradi S. Z. Farzaei M. H. (2020). Astaxanthin, COVID-19 and Immune Response: Focus on Oxidative Stress, Apoptosis and Autophagy. Phytother Res. 34 (11), 2790–2792. 10.1002/ptr.6797 32754955 PMC7436866

[B79] Fang X. K. Gao J. Zhu D. N. (2008). Kaempferol and Quercetin Isolated from Euonymus Alatus Improve Glucose Uptake of 3T3-L1 Cells without Adipogenesis Activity. Life Sci. 82 (11-12), 615–622. 10.1016/j.lfs.2007.12.021 18262572

[B80] Fermin G. (2018). Host Range, Host-Virus Interactions, and Virus Transmission. Viruses 2018, 101–134. 10.1016/b978-0-12-811257-1.00005-x

[B81] Filippini A. D'Amore A. Palombi F. Carpaneto A. (2020). Could the Inhibition of Endo-Lysosomal Two-Pore Channels (TPCs) by the Natural Flavonoid Naringenin Represent an Option to Fight SARS-CoV-2 Infection. Front. Microbiol. 11, 970. 10.3389/fmicb.2020.00970 32425923 PMC7204543

[B82] Frabasile S. Koishi A. C. Kuczera D. Silveira G. F. Verri W. A. Jr. Duarte Dos Santos C. N. (2017). The Citrus Flavanone Naringenin Impairs Dengue Virus Replication in Human Cells. Sci. Rep. 7, 41864. 10.1038/srep41864 28157234 PMC5291091

[B83] Freile M. L. Giannini F. Pucci G. Sturniolo A. Rodero L. Pucci O. (2003). Antimicrobial Activity of Aqueous Extracts and of Berberine Isolated from Berberis Heterophylla. Fitoterapia 74 (7-8), 702–705. 10.1016/s0367-326x(03)00156-4 14630179

[B84] Fujii T. Saito M. (2014). Inhibitory Effect of Quercetin Isolated from Rose Hip (Rosa Canina L.) against Melanogenesis by Mouse Melanoma Cells. Biosci. Biotechnol. Biochem. 73 (9), 1989–1993. 10.1271/bbb.90181 19734679

[B85] Galindez G. Matschinske J. Rose T. D. Sadegh S. Salgado-Albarrán M. Späth J. (2021). Lessons from the COVID-19 Pandemic for Advancing Computational Drug Repurposing Strategies. Nat. Comput. Sci. 1 (1), 33–41. 10.1038/s43588-020-00007-6 38217166

[B86] Galvez J. Crespo M. E. Zarzuelo A. De Witte P. Spiessens C. (1993). Pharmacological Activity of a Procyanidin Isolated fromSclerocarya Birrea Bark: Antidiarrhoeal Activity and Effects on Isolated guinea-pig Ileum. Phytother. Res. 7 (1), 25–28. 10.1002/ptr.2650070108

[B87] Games E. Guerreiro M. Santana F. R. Pinheiro N. M. de Oliveira E. A. Lopes F. D. (2016). Structurally Related Monoterpenes P-Cymene, Carvacrol and Thymol Isolated from Essential Oil from Leaves of Lippia Sidoides Cham. (Verbenaceae) Protect Mice against Elastase-Induced Emphysema. Molecules 21 (10). 10.3390/molecules21101390 PMC6273112 27775634

[B88] Ganbaatar C. Gruner M. Mishig D. Duger R. Schmidt A. W. Knölker H.-J. (2015). Flavonoid Glycosides from the Aerial Parts of Polygonatum Odoratum (Mill.) Druce Growing in Mongolia. Tonpj 8 (1), 1–7. 10.2174/1874848101508010001

[B89] Ganjewala D. Luthra R. (2009). Geranyl Acetate Esterase Controls and Regulates the Level of Geraniol in Lemongrass (Cymbopogon Flexuosus Nees Ex Steud.) Mutant Cv. GRL-1 Leaves. Z. Naturforsch C J. Biosci. 64 (3-4), 251–259. 10.1515/znc-2009-3-417 19526721

[B90] Gao F. Ou H. Y. Chen L. L. Zheng W. X. Zhang C. T. (2003). Prediction of Proteinase Cleavage Sites in Polyproteins of Coronaviruses and its Applications in Analyzing SARS-CoV Genomes. FEBS Lett. 553 (3), 451–456. 10.1016/s0014-5793(03)01091-3 14572668 PMC7232748

[B91] Gao Y. M. Xu G. Wang B. Liu B. C. (2020). Cytokine Storm Syndrome in Coronavirus Disease 2019: A Narrative Review. J. Intern. Med. 289, 147–161. 10.1111/joim.13144 32696489 PMC7404514

[B92] Ghidoli M. Colombo F. Sangiorgio S. Landoni M. Giupponi L. Nielsen E. (2021). Food Containing Bioactive Flavonoids and Other Phenolic or Sulfur Phytochemicals with Antiviral Effect: Can We Design a Promising Diet against COVID-19. Front. Nutr. 8, 661331. 10.3389/fnut.2021.661331 34222300 PMC8247467

[B93] Ghosh R. Chakraborty A. Biswas A. Chowdhuri S. (2020). Identification of Polyphenols from Broussonetia Papyrifera as SARS CoV-2 Main Protease Inhibitors Using In Silico Docking and Molecular Dynamics Simulation Approaches. J. Biomol. Struct. Dyn. 39, 1–14. 10.1080/07391102.2020.1802347 PMC7484588 32762411

[B94] Ghosh S. More P. Derle A. Patil A. B. Markad P. Asok A. (2014). Diosgenin from Dioscorea Bulbifera: Novel Hit for Treatment of Type II Diabetes Mellitus with Inhibitory Activity against α-amylase and α-glucosidase. PLoS ONE 9 (9), e106039. 10.1371/journal.pone.0106039 25216353 PMC4162539

[B95] Gill H. S. Rutherfurd K. J. Cross M. L. Gopal P. K. (2001). Enhancement of Immunity in the Elderly by Dietary Supplementation with the Probiotic Bifidobacterium Lactis HN019. Am. J. Clin. Nutr. 74 (6), 833–839. 10.1093/ajcn/74.6.833 11722966

[B96] Gill S. R. Pop M. DeBoy R. T. Eckburg P. B. Turnbaugh P. J. Samuel B. S. (2006). Metagenomic Analysis of the Human Distal Gut Microbiome. Science 312 (5778), 1355–1359. 10.1126/science.1124234 16741115 PMC3027896

[B97] Gou W. Fu Y. Yue L. Chen G.-d. Cai X. Shuai M. (2020). Gut Microbiota May Underlie the Predisposition of Healthy Individuals to COVID-19-Sensitive Proteomic Biomarkers. 10.1101/2020.04.22.20076091

[B98] Gu S. Chen Y. Wu Z. Chen Y. Gao H. Lv L. (2020b). Alterations of the Gut Microbiota in Patients with Coronavirus Disease 2019 or H1N1 Influenza. Clin. Infect. Dis. 71, 2669–2678. 10.1093/cid/ciaa709 32497191 PMC7314193

[B99] Guh J. H. Ko F. N. Jong T. T. Teng C. M. (1995). Antiplatelet Effect of Gingerol Isolated from Zingiber Officinale. J. Pharm. Pharmacol. 47 (4), 329–332. 10.1111/j.2042-7158.1995.tb05804.x 7791032

[B100] Guo Z. D. Wang Z. Y. Zhang S. F. Li X. Li L. Li C. (2020). Aerosol and Surface Distribution of Severe Acute Respiratory Syndrome Coronavirus 2 in Hospital Wards, Wuhan, China, 2020. Emerg. Infect. Dis. 26 (7), 1583–1591. 10.3201/eid2607.200885 32275497 PMC7323510

[B101] Gupta H. Gupta M. Bhargava S. (2020). Potential Use of Turmeric in COVID‐19. Clin. Exp. Dermatol. 45, 902–903. 10.1111/ced.14357 32608046 PMC7361299

[B102] Gupta R. Mallavarapu G. R. Banerjee S. Kumar S. (2001). Characteristics of an Isomenthone-Rich Somaclonal Mutant Isolated in a Geraniol-Rich Rose-Scented geranium Accession ofPelargonium Graveolens. Flavour Fragr. J. 16 (5), 319–324. 10.1002/ffj.1002

[B103] Hagimori M. Matsumoto T. Mikami Y. (1984). Digitoxin Biosynthesis in Isolated Mesophyll Cells and Cultured Cells of Digitalis. Plant Cel Physiol. 25 (6), 947–953. 10.1093/oxfordjournals.pcp.a076810

[B104] Hamza M. Ali A. Khan S. Ahmed S. Attique Z. Ur Rehman S. (2020). nCOV-19 Peptides Mass Fingerprinting Identification, Binding, and Blocking of Inhibitors Flavonoids and Anthraquinone of Moringa Oleifera and Hydroxychloroquine. J. Biomol. Struct. Dyn., 1–11. 10.1080/07391102.2020.1778534 PMC7332867 32567487

[B105] Han C. Duan C. Zhang S. Spiegel B. Shi H. Wang W. (2020). Digestive Symptoms in COVID-19 Patients with Mild Disease Severity: Clinical Presentation, Stool Viral RNA Testing, and Outcomes. Am. J. Gastroenterol. 115 (6), 916–923. 10.14309/ajg.0000000000000664 32301761 PMC7172493

[B106] Han L. Yuan Y. Zhao L. He Q. Li Y. Chen X. (2012). Tracking Antiangiogenic Components from Glycyrrhiza Uralensis Fisch. Based on Zebrafish Assays Using High-Speed Countercurrent Chromatography. J. Sep. Sci. 35 (9), 1167–1172. 10.1002/jssc.201101031 22555842

[B107] Handa S. S. Borris R. P. Cordell G. A. Phillipson J. D. (2004). NMR Spectral Analysis of Cadambine from Anthocephalus Chinensis. J. Nat. Prod. 46 (3), 325–330. 10.1021/np50027a005

[B108] Hassan S. T. S. (2020). Shedding Light on the Effect of Natural Anti-herpesvirus Alkaloids on SARS-CoV-2: A Treatment Option for COVID-19. Viruses 12 (4), 476. 10.3390/v12040476 PMC7232216 32340120

[B109] He J. Qi W. B. Wang L. Tian J. Jiao P. R. Liu G. Q. (2013). Amaryllidaceae Alkaloids Inhibit Nuclear-To-Cytoplasmic export of Ribonucleoprotein (RNP) Complex of Highly Pathogenic Avian Influenza Virus H5N1. Influenza Other Respir. Viruses 7 (6), 922–931. 10.1111/irv.12035 23136954 PMC4634243

[B110] He Y. Wang J. Li F. Shi Y. (2020). Main Clinical Features of COVID-19 and Potential Prognostic and Therapeutic Value of the Microbiota in SARS-CoV-2 Infections. Front. Microbiol. 11, 1302. 10.3389/fmicb.2020.01302 32582134 PMC7291771

[B111] Heller L. Mota C. R. Greco D. B. (2020). COVID-19 Faecal-Oral Transmission: Are We Asking the Right Questions. Sci. Total Environ. 729, 138919. 10.1016/j.scitotenv.2020.138919 32353720 PMC7182518

[B112] Hibasami H. Shohji T. Shibuya I. Higo K. Kanda T. (2004). Induction of Apoptosis by Three Types of Procyanidin Isolated from Apple (Rosaceae Malus Pumila) in Human Stomach Cancer KATO III Cells. Int. J. Mol. Med. 13, 795. 10.3892/ijmm.13.6.795 15138614

[B113] Ho T. Y. Wu S. L. Chen J. C. Li C. C. Hsiang C. Y. (2007). Emodin Blocks the SARS Coronavirus Spike Protein and Angiotensin-Converting Enzyme 2 Interaction. Antivir. Res 74 (2), 92–101. 10.1016/j.antiviral.2006.04.014 16730806 PMC7114332

[B114] Hoffmann M. Kleine-Weber H. Schroeder S. Krüger N. Herrler T. Erichsen S. (2020). SARS-CoV-2 Cell Entry Depends on ACE2 and TMPRSS2 and Is Blocked by a Clinically Proven Protease Inhibitor. Cell 181 (2), 271. 10.1016/j.cell.2020.02.052 32142651 PMC7102627

[B115] Hong Z. Duan X. Wu S. Yanfang Y. Wu H. (2020). Network Pharmacology Integrated Molecular Docking Reveals the Anti-COVID-19 Mechanism of Qing-Fei-Da-Yuan Granules. Nat. Product. Commun. 15 (6), 1934578X2093421. 10.1177/1934578x20934219

[B116] Hosokawa K. Fukushi E. Kawabata J. Fujii C. Ito T. Yamamura S. (1997). Seven Acylated Anthocyanins in Blue Flowers of Gentiana. Phytochemistry 45 (1), 167–171. 10.1016/s0031-9422(96)00775-3 7766400

[B117] Hu Jun. Ma Wei. Ning Li. Wang K.-J. (2017). Antioxidant and Anti-inflammatory Flavonoids from the Flowers of Chuju, a Medical Cultivar of Chrysanthemum Morifolim Ramat. J. Mex. Chem. Soc. 61 (4), 282–289.

[B118] Hu K. Guan W. J. Bi Y. Zhang W. Li L. Zhang B. (2020). Efficacy and Safety of Lianhuaqingwen Capsules, a Repurposed Chinese Herb, in Patients with Coronavirus Disease 2019: A Multicenter, Prospective, Randomized Controlled Trial. Phytomedicine, 153242. 10.1016/j.phymed.2020.153242 33867046 PMC7229744

[B119] Huang C. Wang Y. Li X. Ren L. Zhao J. Hu Y. (2020). Clinical Features of Patients Infected with 2019 Novel Coronavirus in Wuhan, China. Lancet 395 (10223), 497–506. 10.1016/s0140-6736(20)30183-5 31986264 PMC7159299

[B120] Hyuga S. Hyuga M. Yoshimura M. Amakura Y. Goda Y. Hanawa T. (2013). Herbacetin, A Constituent of Ephedrae Herba, Suppresses the HGF-Induced Motility of Human Breast Cancer MDA-MB-231 Cells by Inhibiting C-Met and Akt Phosphorylation. Planta Med. 79 (16), 1525–1530. 10.1055/s-0033-1350899 24081687

[B121] Hyun J. W. Chung H. S. (2004). Cyanidin and Malvidin from Oryza Sativa Cv. Heugjinjubyeo Mediate Cytotoxicity against Human Monocytic Leukemia Cells by Arrest of G(2)/M Phase and Induction of Apoptosis. J. Agric. Food Chem. 52 (8), 2213–2217. 10.1021/jf030370h 15080622

[B122] Imanshahidi M. Hosseinzadeh H. (2008). Pharmacological and Therapeutic Effects of Berberis Vulgaris and its Active Constituent, Berberine. Phytother Res. 22 (8), 999–1012. 10.1002/ptr.2399 18618524

[B123] Jahng Y. (2013). Progress in the Studies on Tryptanthrin, an Alkaloid of History. Arch. Pharm. Res. 36 (5), 517–535. 10.1007/s12272-013-0091-9 23543631

[B124] Jang D. S. Cuendet M. Hawthorne M. E. Kardono L. B. Kawanishi K. Fong H. H. (2002). Prenylated Flavonoids of the Leaves of Macaranga Conifera with Inhibitory Activity against Cyclooxygenase-2. Phytochemistry 61 (7), 867–872. 10.1016/s0031-9422(02)00378-3 12453581

[B125] Jayaweera M. Perera H. Gunawardana B. Manatunge J. (2020). Transmission of COVID-19 Virus by Droplets and Aerosols: A Critical Review on the Unresolved Dichotomy. Environ. Res. 188, 109819. 10.1016/j.envres.2020.109819 32569870 PMC7293495

[B126] Jiang Y. Yin W. Xu H. E. (2021). RNA-dependent RNA Polymerase: Structure, Mechanism, and Drug Discovery for COVID-19. Biochem. Biophys. Res. Commun. 538, 47–53. 10.1016/j.bbrc.2020.08.116 32943188 PMC7473028

[B127] Jing J. L. J. Pei Yi T. Bose R. J. C. McCarthy J. R. Tharmalingam N. Madheswaran T. (2020). Hand Sanitizers: A Review on Formulation Aspects, Adverse Effects, and Regulations. Int. J. Environ. Res. Public Health 17 (9), 3326. 10.3390/ijerph17093326 PMC7246736 32403261

[B128] Jo S. Kim S. Kim D. Y. Kim M. S. Shin D. H. (2020). Flavonoids with Inhibitory Activity against SARS-CoV-2 3CLpro. J. Enzyme Inhib. Med. Chem. 35 (1), 1539–1544. 10.1080/14756366.2020.1801672 32746637 PMC7470085

[B129] Kadan G. Gözler T. Shamma M. (2004). (-)-Turkiyenine, a New Alkaloid from Chelidonium Majus. J. Nat. Prod. 53 (2), 531–532. 10.1021/np50068a046

[B130] Kadokura K. Suruga K. Tomita T. Hiruma W. Yamada M. Kobayashi A. (2015). Novel Urushiols with Human Immunodeficiency Virus Type 1 Reverse Transcriptase Inhibitory Activity from the Leaves of Rhus Verniciflua. J. Nat. Med. 69 (1), 148–153. 10.1007/s11418-014-0871-7 25349048

[B131] Kahn J. S. McIntosh K. (2005). History and Recent Advances in Coronavirus Discovery. Pediatr. Infect. Dis. J. 24, S223–S227. 10.1097/01.inf.0000188166.17324.60 16378050

[B132] Kakinuma K. Koike J. Kotani K. Ikekawa N. Kada T. Nomoto M. (1984). Cinnamaldehyde: Identification of an Antimutagen from a Crude Drug, Cinnamoni Cortex. Agric. Biol. Chem. 48 (7), 1905–1906. 10.1080/00021369.1984.10866422

[B133] Kamble S. P. Ghadyale V. A. Patil R. S. Haldavnekar V. S. Arvindekar A. U. (2020). Inhibition of GLUT2 Transporter by Geraniol from Cymbopogon Martinii: a Novel Treatment for Diabetes Mellitus in Streptozotocin-Induced Diabetic Rats. J. Pharm. Pharmacol. 72 (2), 294–304. 10.1111/jphp.13194 31737917

[B134] Kang O. H. Choi J. G. Lee J. H. Kwon D. Y. (2010). Luteolin Isolated from the Flowers of *Lonicera japonica* Suppresses Inflammatory Mediator Release by Blocking NF-kappaB and MAPKs Activation Pathways in HMC-1 Cells. Molecules 15 (1), 385–398. 10.3390/molecules15010385 20110898 PMC6257122

[B135] Kang T. H. Moon E. Hong B. N. Choi S. Z. Son M. Park J. H. (2011). Diosgenin from Dioscorea Nipponica Ameliorates Diabetic Neuropathy by Inducing Nerve Growth Factor. Biol. Pharm. Bull. 34 (9), 1493–1498. 10.1248/bpb.34.1493 21881239

[B136] Karimi M. Zarei A. Soleymani S. Jamalimoghadamsiahkali S. Asadi A. Shati M. (2021). Efficacy of Persian Medicine Herbal Formulations (Capsules and Decoction) Compared to Standard Care in Patients with COVID ‐19, a Multicenter Open‐labeled, Randomized, Controlled Clinical Trial. Phytotherapy Res. 10.1002/ptr.7277 PMC8661819 34606123

[B137] Kataoka M. Hirata K. Kunikata T. Ushio S. Iwaki K. Ohashi K. (2001). Antibacterial Action of Tryptanthrin and Kaempferol, Isolated from the Indigo Plant (Polygonum Tinctorium Lour.), against Helicobacter Pylori-Infected Mongolian Gerbils. J. Gastroenterol. 36 (1), 5–9. 10.1007/s005350170147 11211212

[B138] Kaul P. N. Bhattacharya A. K. Rajeswara Rao B. R. Syamasundar K. V. Ramesh S. (2003). Volatile Constituents of Essential Oils Isolated from Different Parts of Cinnamon (Cinnamomum Zeylanicum Blume). J. Sci. Food Agric. 83 (1), 53–55. 10.1002/jsfa.1277

[B139] Kawabata K. Kitamura K. Irie K. Naruse S. Matsuura T. Uemae T. (2017). Triterpenoids Isolated from *Ziziphus Jujuba* Enhance Glucose Uptake Activity in Skeletal Muscle Cells. J. Nutr. Sci. Vitaminol (Tokyo) 63 (3), 193–199. 10.3177/jnsv.63.193 28757534

[B140] Kesic M. J. Simmons S. O. Bauer R. Jaspers I. (2011). Nrf2 Expression Modifies Influenza A Entry and Replication in Nasal Epithelial Cells. Free Radic. Biol. Med. 51 (2), 444–453. 10.1016/j.freeradbiomed.2011.04.027 21549835 PMC3135631

[B141] Keyaerts E. Vijgen L. Pannecouque C. Van Damme E. Peumans W. Egberink H. (2007). Plant Lectins Are Potent Inhibitors of Coronaviruses by Interfering with Two Targets in the Viral Replication Cycle. Antivir. Res 75 (3), 179–187. 10.1016/j.antiviral.2007.03.003 17428553 PMC7114093

[B142] Khalil K. Baharum S. N. Fazry S. Sidik N. M. Sairi F. (2020). Non-Enzymatic Antioxidant from Apple Snail (Pomacea Maculata) Extract. Malays. Appl. Biol. 49 (5), 115–124.

[B143] Khan I. Ullah N. Zha L. Bai Y. Khan A. Zhao T. (2019). Alteration of Gut Microbiota in Inflammatory Bowel Disease (IBD): Cause or Consequence? IBD Treatment Targeting the Gut Microbiome. Pathogens 8 (3), 126. 10.3390/pathogens8030126 PMC6789542 31412603

[B144] Khan S. A. Zia K. Ashraf S. Uddin R. Ul-Haq Z. (2020). Identification of Chymotrypsin-like Protease Inhibitors of SARS-CoV-2 via Integrated Computational Approach. J. Biomol. Struct. Dyn., 1–10. 10.1080/07391102.2020.1751298 32238094

[B145] Khandelwal N. Chander Y. Rawat K. D. Riyesh T. Nishanth C. Sharma S. (2017). Emetine Inhibits Replication of RNA and DNA Viruses without Generating Drug-Resistant Virus Variants. Antivir. Res 144, 196–204. 10.1016/j.antiviral.2017.06.006 28624461

[B146] Khazeei Tabari M. A. Iranpanah A. Bahramsoltani R. Rahimi R. (2021). Flavonoids as Promising Antiviral Agents against SARS-CoV-2 Infection: A Mechanistic Review. Molecules 26 (13), 3900. 10.3390/molecules26133900 34202374 PMC8271800

[B147] Kim J. E. Song Y. J. (2019). Anti-varicella-zoster Virus Activity of Cephalotaxine Esters *In Vitro* . J. Microbiol. 57 (1), 74–79. 10.1007/s12275-019-8514-z 30456755 PMC7090801

[B148] Kimura Y. Matsushita N. Yokoi-Hayashi K. Okuda H. (2001). Effects of Baicalein Isolated from Scutellaria Baicalensis Radix on Adhesion Molecule Expression Induced by Thrombin and Thrombin Receptor Agonist Peptide in Cultured Human Umbilical Vein Endothelial Cells. Planta Med. 67 (4), 331–334. 10.1055/s-2001-14328 11458449

[B149] Kimura Y. Okuda H. Yokoi K. Matsushita N. (1997). Effects of Baicalein Isolated from Roots ofScutellaria Baicalensis Georgi on Interleukin 1β- and Tumour Necrosis Factor α-induced Tissue-type Plasminogen Activator and Plasminogen Activator Inhibitor-1 Production in Cultured Human Umbilical Vein Endothelial Cells. Phytother. Res. 11 (5), 363–367. 10.1002/(sici)1099-1573(199708)11:5<363:Aid-ptr106>3.0.Co;2-u

[B150] Kishore L. Kaur N. Singh R. (2017). Effect of Kaempferol Isolated from Seeds of Eruca Sativa on Changes of Pain Sensitivity in Streptozotocin-Induced Diabetic Neuropathy. Inflammopharmacology 26 (4), 993–1003. 10.1007/s10787-017-0416-2 29159712

[B151] Klann E. Rich S. Mai V. (2020). Gut Microbiota and Coronavirus Disease 2019 (COVID-19): A Superfluous Diagnostic Biomarker or Therapeutic Target. Clin. Infect. Dis. 72, 2247–2248. 10.1093/cid/ciaa1191 PMC7454358 32780788

[B152] Kong Q. Wu Y. Gu Y. Lv Q. Qi F. Gong S. (2020). Analysis of the Molecular Mechanism of Pudilan (PDL) Treatment for COVID-19 by Network Pharmacology Tools. Biomed. Pharmacother. 128, 110316. 10.1016/j.biopha.2020.110316 32505821 PMC7260557

[B153] Kordali S. Cakir A. Ozer H. Cakmakci R. Kesdek M. Mete E. (2008). Antifungal, Phytotoxic and Insecticidal Properties of Essential Oil Isolated from Turkish Origanum Acutidens and its Three Components, Carvacrol, Thymol and P-Cymene. Bioresour. Technol. 99 (18), 8788–8795. 10.1016/j.biortech.2008.04.048 18513954

[B154] Korkmaz H. (2021). Could Sumac Be Effective on COVID-19 Treatment. J. Med. Food 24, 563–568. 10.1089/jmf.2020.0104 32816615

[B155] Kowalczyk M. Golonko A. Świsłocka R. Kalinowska M. Parcheta M. Swiergiel A. (2021). Drug Design Strategies for the Treatment of Viral Disease. Plant Phenolic Compounds and Their Derivatives. Front. Pharmacol. 12, 709104. 10.3389/fphar.2021.709104 34393787 PMC8363300

[B156] Koyama N. Inoue Y. Sekine M. Hayakawa Y. Homma H. Omura S. (2008). Relative and Absolute Stereochemistry of Quinadoline B, an Inhibitor of Lipid Droplet Synthesis in Macrophages. Org. Lett. 10 (22), 5273–5276. 10.1021/ol802089p 18922003

[B157] Kubo I. Fujita K.-i. Nihei K.-i. (2008). Antimicrobial Activity of Anethole and Related Compounds from Aniseed. J. Sci. Food Agric. 88 (2), 242–247. 10.1002/jsfa.3079

[B158] Kulkarni S. A. Nagarajan S. K. Ramesh V. Palaniyandi V. Selvam S. P. Madhavan T. (2020). Computational Evaluation of Major Components from Plant Essential Oils as Potent Inhibitors of SARS-CoV-2 Spike Protein. J. Mol. Struct. 1221, 128823. 10.1016/j.molstruc.2020.128823 32834111 PMC7334662

[B159] Kumar A. Chowdhury S. R. Jatte K. K. Chakrabarti T. Majumder H. K. Jha T. (2015). Anthocephaline, a New Indole Alkaloid and Cadambine, a Potent Inhibitor of DNA Topoisomerase IB of Leishmania Donovani (LdTOP1LS), Isolated from Anthocephalus Cadamba. Nat. Prod. Commun. 10 (2), 297–299. 10.1177/1934578x1501000221 25920266

[B160] Kumar D. Jain A. Verma A. (2017). Phytochemical and Pharmacological Investigation of Cassia Siamea Lamk: An Insight. Npj 7 (4). 10.2174/2210315507666170509125800

[B161] Kumar N. Sood D. van der Spek P. J. Sharma H. S. Chandra R. (2020a). Molecular Binding Mechanism and Pharmacology Comparative Analysis of Noscapine for Repurposing against SARS-CoV-2 Protease. J. Proteome Res. 19 (11), 4678–4689. 10.1021/acs.jproteome.0c00367 32786685

[B162] Kumar S. Kashyap P. Chowdhury S. Kumar S. Panwar A. Kumar A. (2020b). Identification of Phytochemicals as Potential Therapeutic Agents that Binds to Nsp15 Protein Target of Coronavirus (SARS-CoV-2) that Are Capable of Inhibiting Virus Replication. Phytomedicine 153317, 153317. 10.1016/j.phymed.2020.153317 PMC7470885 32943302

[B163] Kunle O. Okogun J. Egamana E. Emojevwe E. Shok M. (2003). Antimicrobial Activity of Various Extracts and Carvacrol from Lippia Multiflora Leaf Extract. Phytomedicine 10 (1), 59–61. 10.1078/094471103321648674 12622465

[B164] Kuppusamy P. Lee K. D. Song C. E. Ilavenil S. Srigopalram S. Arasu M. V. (2018). Quantification of Major Phenolic and Flavonoid Markers in Forage Crop Lolium Multiflorum Using HPLC-DAD. Revista Brasileira de Farmacognosia 28 (3), 282–288. 10.1016/j.bjp.2018.03.006

[B165] Lai X. Wang M. Qin C. Tan L. Ran L. Chen D. (2020a). Coronavirus Disease 2019 (COVID-2019) Infection Among Health Care Workers and Implications for Prevention Measures in a Tertiary Hospital in Wuhan, China. JAMA Netw. Open 3 (5), e209666. 10.1001/jamanetworkopen.2020.9666 32437575 PMC7243089

[B166] Lai Y. Yan Y. Liao S. Li Y. Ye Y. Liu N. (2020b). 3D-quantitative Structure-Activity Relationship and Antiviral Effects of Curcumin Derivatives as Potent Inhibitors of Influenza H1N1 Neuraminidase. Arch. Pharm. Res. 43 (5), 489–502. 10.1007/s12272-020-01230-5 32248350 PMC7125423

[B167] Lane G. A. Sutherland O. R. Skipp R. A. (1987). Isoflavonoids as Insect Feeding Deterrents and Antifungal Components from Root ofLupinus Angustifolius. J. Chem. Ecol. 13 (4), 771–783. 10.1007/bf01020159 24302045

[B168] Larit F. Elokely K. M. Nael M. A. Benyahia S. León F. Cutler S. J. (2021). Proposed Mechanism for the Antitrypanosomal Activity of Quercetin and Myricetin Isolated from Hypericum Afrum Lam.: Phytochemistry, *In Vitro* Testing and Modeling Studies. Molecules 26 (4), 1009. 10.3390/molecules26041009 33672916 PMC7918497

[B169] Law S. Leung A. W. Xu C. (2020). Is the Traditional Chinese Herb "Artemisia Annua" Possible to Fight against COVID-19. Integr. Med. Res. 9 (3), 100474. 10.1016/j.imr.2020.100474 32742919 PMC7362865

[B170] Lee B. Kwon M. Choi J. S. Jeong H. O. Chung H. Y. Kim H. R. (2015a). Kaempferol Isolated from *Nelumbo nucifera* Inhibits Lipid Accumulation and Increases Fatty Acid Oxidation Signaling in Adipocytes. J. Med. Food 18 (12), 1363–1370. 10.1089/jmf.2015.3457 26280739

[B171] Lee I. A. Lee J. H. Baek N. I. Kim D. H. (2005). Antihyperlipidemic Effect of Crocin Isolated from the Fructus of Gardenia Jasminoides and its Metabolite Crocetin. Biol. Pharm. Bull. 28 (11), 2106–2110. 10.1248/bpb.28.2106 16272698

[B172] Lee J. H. Kim M. Chang K. H. Hong C. Y. Na C. S. Dong M. S. (2015b). Antiplatelet Effects of Rhus Verniciflua Stokes Heartwood and its Active Constituents-Ffisetin, Butein, and Sulfuretin-Iin Rats. J. Med. Food 18 (1), 21–30. 10.1089/jmf.2013.3116 25372471

[B173] Lee J. S. Shin E. C. (2020). The Type I Interferon Response in COVID-19: Implications for Treatment. Nat. Rev. Immunol. 20 (10), 585–586. 10.1038/s41577-020-00429-3 32788708 PMC8824445

[B174] Li J. Jiang Y. (2007). Litchi Flavonoids: Isolation, Identification and Biological Activity. Molecules 12 (4), 745–758. 10.3390/12040745 17851427 PMC6149383

[B175] Li L.-C. Zhang Z.-H. Zhou W.-C. Chen J. Jin H.-Q. Fang H.-M. (2020a). Lianhua Qingwen Prescription for Coronavirus Disease 2019 (COVID-19) Treatment: Advances and Prospects. Biomed. Pharmacother. 130, 110641. 10.1016/j.biopha.2020.110641 34321172 PMC7437484

[B176] Li Q. Guan X. Wu P. Wang X. Zhou L. Tong Y. (2020c). Early Transmission Dynamics in Wuhan, China, of Novel Coronavirus-Infected Pneumonia. N. Engl. J. Med. 382 (13), 1199–1207. 10.1056/NEJMoa2001316 31995857 PMC7121484

[B177] Li S. Y. Chen C. Zhang H. Q. Guo H. Y. Wang H. Wang L. (2005). Identification of Natural Compounds with Antiviral Activities against SARS-Associated Coronavirus. Antivir. Res 67 (1), 18–23. 10.1016/j.antiviral.2005.02.007 15885816 PMC7114104

[B178] Lim H. Son K. H. Chang H. W. Bae K. Kang S. S. Kim H. P. (2008). Anti-inflammatory Activity of Pectolinarigenin and Pectolinarin Isolated from Cirsium Chanroenicum. Biol. Pharm. Bull. 31 (11), 2063–2067. 10.1248/bpb.31.2063 18981574

[B179] Lin S. C. Ho C. T. Chuo W. H. Li S. Wang T. T. Lin C. C. (2017). Effective Inhibition of MERS-CoV Infection by Resveratrol. BMC Infect. Dis. 17 (1), 144. 10.1186/s12879-017-2253-8 28193191 PMC5307780

[B180] Liolios C. C. Gortzi O. Lalas S. Tsaknis J. Chinou I. (2009). Liposomal Incorporation of Carvacrol and Thymol Isolated from the Essential Oil of Origanum Dictamnus L. And *In Vitro* Antimicrobial Activity. Food Chem. 112 (1), 77–83. 10.1016/j.foodchem.2008.05.060

[B181] Liu I. M. Liou S. S. Lan T. W. Hsu F. L. Cheng J. T. (2005). Myricetin as the Active Principle of Abelmoschus Moschatus to Lower Plasma Glucose in Streptozotocin-Induced Diabetic Rats. Planta Med. 71 (7), 617–621. 10.1055/s-2005-871266 16041646

[B182] Liu J. Yang Y. Xu Y. Ma C. Qin C. Zhang L. (2011a). Lycorine Reduces Mortality of Human Enterovirus 71-infected Mice by Inhibiting Virus Replication. Virol. J. 8, 483. 10.1186/1743-422X-8-483 22029605 PMC3212826

[B183] Liu L. K. (1996). Selective Isolation of Anethole from Fructus Anisi Stellati(star Anise) by Supercritical Fluid Extraction. Anal. Commun. 33 (5), 175. 10.1039/ac9963300175

[B184] Liu Q. Zhang Y. Long Y. (2020a). A Child Infected with Severe Acute Respiratory Syndrome Coronavirus 2 Presenting with Diarrhea without Fever and Cough: A Case Report. Medicine (Baltimore) 99 (33), e21427. 10.1097/MD.0000000000021427 32871990 PMC7437845

[B185] Liu R. Meng F. Zhang L. Liu A. Qin H. Lan X. (2011b). Luteolin Isolated from the Medicinal Plant Elsholtzia Rugulosa (Labiatae) Prevents Copper-Mediated Toxicity in β-amyloid Precursor Protein Swedish Mutation Overexpressing SH-Sy5y Cells. Molecules 16 (3), 2084–2096. 10.3390/molecules16032084 21368720 PMC6259644

[B186] Liu X. Park J. H. Abd El-Aty A. M. Assayed M. E. Shimoda M. Shim J. H. (2013). Isolation of Volatiles from Nigella Sativa Seeds Using Microwave-Assisted Extraction: Effect of Whole Extracts on Canine and Murine CYP1A. Biomed. Chromatogr. 27 (7), 938–945. 10.1002/bmc.2887 23629843

[B187] Liu X. Cheng J. Zhao N. Liu Z. (2014). Insecticidal Activity of Essential Oil of Cinnamomum cassia and its Main Constituent, Trans-cinnamaldehyde, against the Booklice, Liposcelis Bostrychophila. Trop. J. Pharm. Res. 13 (10), 1697. 10.4314/tjpr.v13i10.18

[B188] Liu Y. C. Kuo R. L. Shih S. R. (2020b). COVID-19: The First Documented Coronavirus Pandemic in History. Biomed. J. 43 (4), 328–333. 10.1016/j.bj.2020.04.007 32387617 PMC7199674

[B189] Lodhi S. Singhai A. K. (2013). Wound Healing Effect of Flavonoid Rich Fraction and Luteolin Isolated from Martynia Annua Linn. On Streptozotocin Induced Diabetic Rats. Asian Pac. J. Trop. Med. 6 (4), 253–259. 10.1016/s1995-7645(13)60053-x 23608325

[B190] Lodish H. Berk A. Zipursky S. L. Matsudaira P. Baltimore D. Darnell J. (2000). Viruses: Structure, Function, and Uses. Available at: https://www.ncbi.nlm.nih.gov/books/NBK21523/ .

[B191] Lombard K. Peffley E. Geoffriau E. Thompson L. Herring A. (2005). Quercetin in Onion (Allium cepa L.) after Heat-Treatment Simulating home Preparation. J. Food Compost. Anal. 18 (6), 571–581. 10.1016/j.jfca.2004.03.027

[B192] Luo P. Liu D. Li J. (2020). Pharmacological Perspective: Glycyrrhizin May Be an Efficacious Therapeutic Agent for COVID-19. Int. J. Antimicrob. Agents 55 (6), 105995. 10.1016/j.ijantimicag.2020.105995 32335281 PMC7180159

[B193] Machado D. G. Bettio L. E. Cunha M. P. Santos A. R. Pizzolatti M. G. Brighente I. M. (2008). Antidepressant-like Effect of Rutin Isolated from the Ethanolic Extract from Schinus Molle L. In Mice: Evidence for the Involvement of the Serotonergic and Noradrenergic Systems. Eur. J. Pharmacol. 587 (1-3), 163–168. 10.1016/j.ejphar.2008.03.021 18457827

[B194] Maddah M. Bahramsoltani R. Yekta N. H. Rahimi R. Aliabadi R. Pourfath M. (2021). Proposing High-Affinity Inhibitors from Glycyrrhiza Glabra L. Against SARS-CoV-2 Infection: Virtual Screening and Computational Analysis. New J. Chem. 45 (35), 15977–15995. 10.1039/d1nj02031e

[B195] Maiti S. Banerjee A. Nazmeen A. Kanwar M. Das S. (2020). Active-site Molecular Docking of Nigellidine with Nucleocapsid- NSP2-MPro of COVID-19 and to Human IL1R-IL6R and strong Antioxidant Role of Nigella-Sativa in Experimental Rats. J. Drug Target., 1–23. 10.1080/1061186X.2020.1817040 32875925

[B196] Mantlo E. Bukreyeva N. Maruyama J. Paessler S. Huang C. (2020). Antiviral Activities of Type I Interferons to SARS-CoV-2 Infection. Antivir. Res 179, 104811. 10.1016/j.antiviral.2020.104811 32360182 PMC7188648

[B197] Marinella M. A. (2020). Indomethacin and Resveratrol as Potential Treatment Adjuncts for SARS-CoV-2/covid-19. Int. J. Clin. Pract. 74 (9), e13535. 10.1111/ijcp.13535 32412158 PMC7261995

[B198] Maroli N. Bhasuran B. Natarajan J. Kolandaivel P. (2020). The Potential Role of Procyanidin as a Therapeutic Agent against SARS-CoV-2: a Text Mining, Molecular Docking and Molecular Dynamics Simulation Approach. J. Biomol. Struct. Dyn., 1–16. 10.1080/07391102.2020.1823887 PMC7544928 32960159

[B199] Martínez-Vázquez M. Apan T. Lastra A. Bye R. (2007). A Comparative Study of the Analgesic and Anti-inflammatory Activities of Pectolinarin Isolated fromCirsium Subcoriaceumand Linarin Isolated fromBuddleia Cordata. Planta Med. 64 (02), 134–137. 10.1055/s-2006-957390 9525105

[B200] Matsuo T. Ito S. (2014). The Chemical Structure of Kaki-Tannin from Immature Fruit of the Persimmon (Diospyros Kaki L.). Agric. Biol. Chem. 42 (9), 1637–1643. 10.1080/00021369.1978.10863225

[B201] Matter H. Sotriffer C. (2011). “Applications and Success Stories in Virtual Screening,” in Virtual Screening., 319–358. 10.1002/9783527633326.ch12

[B202] McBride R. van Zyl M. Fielding B. C. (2014). The Coronavirus Nucleocapsid Is a Multifunctional Protein. Viruses 6 (8), 2991–3018. 10.3390/v6082991 25105276 PMC4147684

[B203] Mekala A. B. Satyal P. Setzer W. N. (2017). Phytochemicals from the Bark of Rhamnus Caroliniana. Nat. Prod. Commun. 12 (3), 403–406. 10.1177/1934578x1701200324 30549896

[B204] Mendonca P. Soliman K. F. A. (2020). Flavonoids Activation of the Transcription Factor Nrf2 as a Hypothesis Approach for the Prevention and Modulation of SARS-CoV-2 Infection Severity. Antioxidants (Basel) 9 (8), 659. 10.3390/antiox9080659 PMC7463602 32722164

[B205] Mete I. E. Gözler T. (2004). (+)-Oxoturkiyenine: an Isoquinoline-Derived Alkaloid from Hypecoum Pendulum. J. Nat. Prod. 51 (2), 272–274. 10.1021/np50056a013

[B206] Miyazawa M. Shimamura H. Nakamura S.-i. Kameoka H. (1996). Antimutagenic Activity of (+)-β-Eudesmol and Paeonol from Dioscorea Japonica. J. Agric. Food Chem. 44 (7), 1647–1650. 10.1021/jf950792u

[B207] Mody V. Ho J. Wills S. Mawri A. Lawson L. Ebert M. C. C. J. C. (2021). Identification of 3-chymotrypsin like Protease (3CLPro) Inhibitors as Potential Anti-SARS-CoV-2 Agents. Commun. Biol. 4 (1), 93. 10.1038/s42003-020-01577-x 33473151 PMC7817688

[B208] Mohamed K. Yazdanpanah N. Saghazadeh A. Rezaei N. (2021). Computational Drug Discovery and Repurposing for the Treatment of COVID-19: A Systematic Review. Bioorg. Chem. 106, 104490. 10.1016/j.bioorg.2020.104490 33261845 PMC7676368

[B209] Mohammed A. Gbonjubola V. A. Koorbanally N. A. Islam M. S. (2017). Inhibition of Key Enzymes Linked to Type 2 Diabetes by Compounds Isolated from Aframomum Melegueta Fruit. Pharm. Biol. 55 (1), 1010–1016. 10.1080/13880209.2017.1286358 28176546 PMC6130490

[B210] Mohn T. Plitzko I. Hamburger M. (2009). A Comprehensive Metabolite Profiling of Isatis Tinctoria Leaf Extracts. Phytochemistry 70 (7), 924–934. 10.1016/j.phytochem.2009.04.019 19467550

[B211] Mollica V. Rizzo A. Massari F. (2020). The Pivotal Role of TMPRSS2 in Coronavirus Disease 2019 and Prostate Cancer. Future Oncol. 16 (27), 2029–2033. 10.2217/fon-2020-0571 32658591 PMC7359420

[B212] Montenegro-Landívar M. F. Tapia-Quirós P. Vecino X. Reig M. Valderrama C. Granados M. (2021). Polyphenols and Their Potential Role to Fight Viral Diseases: An Overview. Sci. Total Environ. 801, 149719. 10.1016/j.scitotenv.2021.149719 34438146 PMC8373592

[B213] Mousavizadeh L. Ghasemi S. (2021). Genotype and Phenotype of COVID-19: Their Roles in Pathogenesis. J. Microbiol. Immunol. Infect. 54, 159–163. 10.1016/j.jmii.2020.03.022 32265180 PMC7138183

[B214] Mu C. Sheng Y. Wang Q. Amin A. Li X. Xie Y. (202110414). Potential Compound from Herbal Food of Rhizoma Polygonati for Treatment of COVID-19 Analyzed by Network Pharmacology: Viral and Cancer Signaling Mechanisms. J. Funct. Foods 77, 104149. 10.1016/j.jff.2020.104149 PMC7427583 32837538

[B215] Mudd P. A. Crawford J. C. Turner J. S. Souquette A. Reynolds D. Bender D. (2020). Distinct Inflammatory Profiles Distinguish COVID-19 from Influenza with Limited Contributions from Cytokine Storm. Sci. Adv. 6 (50), eabe3024. 10.1126/sciadv.abe3024 33187979 PMC7725462

[B216] Mukhopadhyay R. Roy S. Venkatadri R. Su Y. P. Ye W. Barnaeva E. (2016). Efficacy and Mechanism of Action of Low Dose Emetine against Human Cytomegalovirus. Plos Pathog. 12 (6), e1005717. 10.1371/journal.ppat.1005717 27336364 PMC4919066

[B217] Muratov E. N. Amaro R. Andrade C. H. Brown N. Ekins S. Fourches D. (2021). A Critical Overview of Computational Approaches Employed for COVID-19 Drug Discovery. Chem. Soc. Rev. 50 (16), 9121–9151. 10.1039/d0cs01065k 34212944 PMC8371861

[B218] Nagpal R. Mainali R. Ahmadi S. Wang S. Singh R. Kavanagh K. (2018). Gut Microbiome and Aging: Physiological and Mechanistic Insights. Nutr. Healthy Aging 4 (4), 267–285. 10.3233/nha-170030 29951588 PMC6004897

[B219] Nahmias Y. Goldwasser J. Casali M. van Poll D. Wakita T. Chung R. T. (2008). Apolipoprotein B-dependent Hepatitis C Virus Secretion Is Inhibited by the Grapefruit Flavonoid Naringenin. Hepatology 47 (5), 1437–1445. 10.1002/hep.22197 18393287 PMC4500072

[B220] Natarajan S. Anbarasi C. Sathiyarajeswaran P. Manickam P. Geetha S. Kathiravan R. (2021). Kabasura Kudineer (KSK), a Poly-Herbal Siddha Medicine, Reduced SARS-CoV-2 Viral Load in Asymptomatic COVID-19 Individuals as Compared to Vitamin C and Zinc Supplementation: Findings from a Prospective, Exploratory, Open-Labeled, Comparative, Randomized Controlled Trial, Tamil Nadu, India. Trials 22 (1), 623. 10.1186/s13063-021-05583-0 34526104 PMC8441246

[B221] Nazaruk J. Orlikowski P. (2015). Phytochemical Profile and Therapeutic Potential of Viscum Album L. Nat. Prod. Res. 30 (4), 373–385. 10.1080/14786419.2015.1022776 25813519

[B222] Negi S. Das D. K. Pahari S. Nadeem S. Agrewala J. N. (2019). Potential Role of Gut Microbiota in Induction and Regulation of Innate Immune Memory. Front. Immunol. 10, 2441. 10.3389/fimmu.2019.02441 31749793 PMC6842962

[B223] Nezhadali A. Akbarpour M. Shirvan B. Z. (2008). Chemical Composition of the Essential Oil from the Aerial Parts ofArtemisia Herba. E-Journal Chem. 5 (3), 557–561. 10.1155/2008/730453

[B224] Ngwa W. Kumar R. Thompson D. Lyerly W. Moore R. Reid T. E. (2020). Potential of Flavonoid-Inspired Phytomedicines against COVID-19. Molecules 25 (11), 2707. 10.3390/molecules25112707 PMC7321405 32545268

[B225] Ni J. Xiao H. Weng L. Wei X. Xu Y. (2011). Blocking Group-Directed Diastereoselective Total Synthesis of (±)-α-Noscapine. Tetrahedron 67 (29), 5162–5167. 10.1016/j.tet.2011.05.060

[B226] Obrenovich M. E. M. (2018). Leaky Gut, Leaky Brain. Microorganisms 6 (4), 107. 10.3390/microorganisms6040107 PMC6313445 30340384

[B227] Ohkoshi E. Nagashima T. Sato H. Fujii Y. Nozawa K. Nagai M. (2009). Simple Preparation of Baicalin from Scutellariae Radix. J. Chromatogr. A. 1216 (11), 2192–2194. 10.1016/j.chroma.2008.03.059 18407279

[B228] Olennikov D. Partilkhaev V. (2012). Isolation and Densitometric HPTLC Analysis of Rutin, Narcissin, Nicotiflorin, and Isoquercitrin inCaragana Spinosashoots. J. Planar Chromatogr. - Mod. TLC 25 (1), 30–35. 10.1556/jpc.25.2012.1.5

[B229] Olivar J. E. Sy K. A. Villanueva C. V. Alejandro G. J. D. Tan M. A. (2018). Alkaloids as Chemotaxonomic Markers from the Philippine Endemic Uncaria Perrottetii and Uncaria Lanosa F. Philippinensis. J. King Saud Univ. - Sci. 30 (2), 283–285. 10.1016/j.jksus.2017.12.008

[B230] Omrani A. S. Saad M. M. Baig K. Bahloul A. Abdul-Matin M. Alaidaroos A. Y. (2014). Ribavirin and Interferon Alfa-2a for Severe Middle East Respiratory Syndrome Coronavirus Infection: a Retrospective Cohort Study. Lancet Infect. Dis. 14 (11), 1090–1095. 10.1016/s1473-3099(14)70920-x 25278221 PMC7106357

[B231] Oso B. J. Adeoye A. O. Olaoye I. F. (2020). Pharmacoinformatics and Hypothetical Studies on Allicin, Curcumin, and Gingerol as Potential Candidates against COVID-19-Associated Proteases. J. Biomol. Struct. Dyn., 1–12. 10.1080/07391102.2020.1813630 32876538

[B232] Park H. R. Yoon H. Kim M. K. Lee S. D. Chong Y. (2012). Synthesis and Antiviral Evaluation of 7-O-Arylmethylquercetin Derivatives against SARS-Associated Coronavirus (SCV) and Hepatitis C Virus (HCV). Arch. Pharm. Res. 35 (1), 77–85. 10.1007/s12272-012-0108-9 22297745 PMC7090976

[B233] Peng-fei L. Fu-gen H. Bin-bin D. Tian-sheng D. Xiang-lin H. Ming-qin Z. (2012). Purification and Antioxidant Activities of Baicalin Isolated from the Root of Huangqin (Scutellaria Baicalensis Gcorsi). J. Food Sci. Technol. 50 (3), 615–619. 10.1007/s13197-012-0857-y 24425963 PMC3602557

[B234] Péter Zomborszki Z. Kúsz N. Csupor D. Peschel W. (2019). Rhodiosin and Herbacetin in Rhodiola Rosea Preparations: Additional Markers for Quality Control. Pharm. Biol. 57 (1), 295–305. 10.1080/13880209.2019.1577460 31356124 PMC6711108

[B235] Phillips M. A. Stewart M. A. Woodling D. L. Xie Z. -R. (2018). “Has Molecular Docking Ever Brought us a Medicine,” in Molecular Docking.

[B236] Phillipson J. D. Hemingway S. R. (1973). Indole and Oxindole Alkaloids from Uncaria Bernaysia. Phytochemistry 12 (6), 1481–1487. 10.1016/0031-9422(73)80588-6

[B237] Prakash S. Elavarasan N. Subashini K. Kanaga S. Dhandapani R. Sivanandam M. (2020). Isolation of Hesperetin - A Flavonoid from Cordia Sebestena Flower Extract through Antioxidant Assay Guided Method and its Antibacterial, Anticancer Effect on Cervical Cancer via *In Vitro* and In Silico Molecular Docking Studies. J. Mol. Struct. 1207, 127751. 10.1016/j.molstruc.2020.127751

[B238] Prasad A. Muthamilarasan M. Prasad M. (2020). Synergistic Antiviral Effects against SARS-CoV-2 by Plant-Based Molecules. Plant Cel Rep 39 (9), 1109–1114. 10.1007/s00299-020-02560-w PMC7303273 32561979

[B239] Prasad S. Tyagi A. K. (2015). Curcumin and its Analogues: a Potential Natural Compound against HIV Infection and AIDS. Food Funct. 6 (11), 3412–3419. 10.1039/c5fo00485c 26404185

[B240] Przekwas A. Chen Z. (2020). Washing Hands and the Face May Reduce COVID-19 Infection. Med. Hypotheses 144, 110261. 10.1016/j.mehy.2020.110261 33254560 PMC7481347

[B241] Qi W. Yue S. J. Sun J. H. Simpkins J. W. Zhang L. Yuan D. (2014). Alkaloids from the Hook-Bearing branch of Uncariarhynchophylla and Their Neuroprotective Effects against Glutamate-Induced HT22 Cell Death. J. Asian Nat. Prod. Res. 16 (8), 876–883. 10.1080/10286020.2014.918109 24899363 PMC4446702

[B242] Quimque M. T. J. Notarte K. I. R. Fernandez R. A. T. Mendoza M. A. O. Liman R. A. D. Lim J. A. K. (2020). Virtual Screening-Driven Drug Discovery of SARS-CoV2 Enzyme Inhibitors Targeting Viral Attachment, Replication, post-translational Modification and Host Immunity Evasion Infection Mechanisms. J. Biomol. Struct. Dyn. 39, 1–18. 10.1080/07391102.2020.1776639 PMC7309309 32476574

[B243] Rabehaja D. J. Ihandriharison H. Ramanoelina P. A. Ratsimamanga-Urverg S. Bighelli A. Casanova J. (2013). Leaf Oil from Vepris Madagascarica (Rutaceae), Source of (E)-Anethole. Nat. Prod. Commun. 8 (8), 1165–1166. 10.1177/1934578x1300800835 24079195

[B244] Raeiszadeh M. Adeli B. (2020). A Critical Review on Ultraviolet Disinfection Systems against COVID-19 Outbreak: Applicability, Validation, and Safety Considerations. ACS Photon. 7 (11), 2941–2951. 10.1021/acsphotonics.0c01245 37556269

[B245] Rajput M. S. Mathur V. Agrawal P. Chandrawanshi H. K. Pilaniya U. (2011). Fibrinolytic Activity of Kaempferol Isolated from the Fruits of Lagenaria Siceraria (Molina) Standley. Nat. Prod. Res. 25 (19), 1870–1875. 10.1080/14786419.2010.540760 21861768

[B246] Reichling J. Neuner A. Sharaf M. Harkenthal M. Schnitzler P. (2009). Antiviral Activity of Rhus Aromatica (Fragrant Sumac) Extract against Two Types of Herpes Simplex Viruses in Cell Culture. Pharmazie 64 (8), 538–541. 19746844

[B247] Richart S. M. Li Y. L. Mizushina Y. Chang Y. Y. Chung T. Y. Chen G. H. (2018). Synergic Effect of Curcumin and its Structural Analogue (Monoacetylcurcumin) on Anti-influenza Virus Infection. J. Food Drug Anal. 26 (3), 1015–1023. 10.1016/j.jfda.2017.12.006 29976394 PMC9303033

[B248] Riou J. Althaus C. L. (2020). Pattern of Early Human-To-Human Transmission of Wuhan 2019 Novel Coronavirus (2019-nCoV), December 2019 to January 2020. Euro Surveill. 25 (4), 2000058. 10.2807/1560-7917.Es.2020.25.4.2000058 PMC7001239 32019669

[B249] Rooban B. N. Sasikala V. Gayathri Devi V. Sahasranamam V. Abraham A. (2012). Prevention of Selenite Induced Oxidative Stress and Cataractogenesis by Luteolin Isolated from Vitex Negundo. Chem. Biol. Interact 196 (1-2), 30–38. 10.1016/j.cbi.2012.01.005 22342831

[B250] Rooks M. G. Garrett W. S. (2016). Gut Microbiota, Metabolites and Host Immunity. Nat. Rev. Immunol. 16 (6), 341–352. 10.1038/nri.2016.42 27231050 PMC5541232

[B251] Rücker G. Breitmaier E. Zhang G.-L. Mayer R. (1994). Alkaloids from Dactylicapnos Torulosa. Phytochemistry 36 (2), 519–523. 10.1016/s0031-9422(00)97106-1

[B252] Sadraei H. Asghari G. Emami S. (2013). Inhibitory Effect of Rosa Damascena Mill Flower Essential Oil, Geraniol and Citronellol on Rat Ileum Contraction. Res. Pharm. Sci. 8 (1), 17–23. 24459472 PMC3895296

[B253] Saeed S. A. Farnaz S. Simjee R. U. Malik A. (1993). Triterpenes and B-Sitosterol from Piper Betle: Isolation, Antiplatelet and Anti-inflammatory Effects. Biochem. Soc. Trans. 21 (4), 462S. 10.1042/bst021462s 8132030

[B254] Saitoh T. Noguchi H. Shibata S. (1978). A New Isoflavone and the Corresponding Isoflavanone of Licorice Root. Chem. Pharm. Bull. 26 (1), 144–147. 10.1248/cpb.26.144

[B255] Sakurai Y. Kolokoltsov A. A. Chen C. C. Tidwell M. W. Bauta W. E. Klugbauer N. (2015). Ebola Virus. Two-Pore Channels Control Ebola Virus Host Cell Entry and Are Drug Targets for Disease Treatment. Science 347 (6225), 995–998. 10.1126/science.1258758 25722412 PMC4550587

[B256] Sato Y. Latham H. G. (2002). The Isolation of Diosgenin from Solanum Xanthocarpum1. J. Am. Chem. Soc. 75 (23), 6067. 10.1021/ja01119a532

[B257] Schirmer M. Smeekens S. P. Vlamakis H. Jaeger M. Oosting M. Franzosa E. A. (2016). Linking the Human Gut Microbiome to Inflammatory Cytokine Production Capacity. Cell 167 (4), 1897–1136. 10.1016/j.cell.2016.10.02010.1016/j.cell.2016.11.046 27984736

[B258] Selim S. Al Jaouni S. (2015). Anticancer and Apoptotic Effects on Cell Proliferation of Diosgenin Isolated from Costus Speciosus (Koen.) Sm. BMC Complement. Altern. Med. 15 (1), 301. 10.1186/s12906-015-0836-8 26329920 PMC4556405

[B259] Shah S. B. Hariharan U. Chawla R. (2021). Common Anti-COVID-19 Drugs and Their Anticipated Interaction with Anesthetic Agents. J. Anaesthesiol Clin. Pharmacol. 37 (2), 160–170. 10.4103/joacp.JOACP_461_20 34349362 PMC8289657

[B260] Shahat A. A. (2008). Procyanidins fromAdansonia Digitata. Pharm. Biol. 44 (6), 445–450. 10.1080/13880200600798510

[B261] Shang Z. H. Hou Y. Long R. J. (2012). Chemical Composition of Essential Oil of Artemisia Nanschanica Krasch. From Tibetan Plateau. Ind. Crops Prod. 40, 35–38. 10.1016/j.indcrop.2012.02.027

[B262] Shengqiang T. Jizhong Y. Gang C. Lou J. (2009). Purification of Rutin and Nicotiflorin from the Flowers of Edgeworthia Chrysantha Lindl. By High-Speed Counter-current Chromatography. J. Chromatogr. Sci. 47 (5), 341–344. 10.1093/chromsci/47.5.341 19476699

[B263] Shervington L. A. Li B. S. Shervington A. A. Alpan N. Patel R. Muttakin U. (2018). A Comparative HPLC Analysis of Myricetin, Quercetin and Kaempferol Flavonoids Isolated from Gambian and Indian Moringa Oleifera Leaves. Ijc 10 (4), 28. 10.5539/ijc.v10n4p28

[B264] Shin D. Mukherjee R. Grewe D. Bojkova D. Baek K. Bhattacharya A. (2020). Papain-like Protease Regulates SARS-CoV-2 Viral Spread and Innate Immunity. Nature 587 (7835), 657–662. 10.1038/s41586-020-2601-5 32726803 PMC7116779

[B265] Shirataki Y. Yokoe I. Noguchi M. Tomimori T. Komatsu M. (1988). Studies on the Constituents of Sophora Species. XXII. Constituents of the Root of Sophora Moorcroftiana BENTH. Ex BAKER.(1). Chem. Pharm. Bull. 36 (6), 2220–2225. 10.1248/cpb.36.2220

[B266] Shirole R. L. Shirole N. L. Saraf M. N. (2015). *In Vitro* relaxant and Spasmolytic Effects of Essential Oil of Pistacia Integerrima Stewart Ex Brandis Galls. J. Ethnopharmacol 168, 61–65. 10.1016/j.jep.2015.02.001 25732838

[B267] Shoji T. Mutsuga M. Nakamura T. Kanda T. Akiyama H. Goda Y. (2003). Isolation and Structural Elucidation of Some Procyanidins from Apple by Low-Temperature Nuclear Magnetic Resonance. J. Agric. Food Chem. 51 (13), 3806–3813. 10.1021/jf0300184 12797747

[B268] Silverstein L. J. Swanson B. G. Moffett D. (1996). Procyanidin from Black Beans (Phaseolus vulgaris) Inhibits Nutrient and Electrolyte Absorption in Isolated Rat Ileum and Induces Secretion of Chloride Ion. J. Nutr. 126 (6), 1688–1695. 10.1093/jn/126.6.1688 8648444

[B269] Singh R. Singh B. Singh S. Kumar N. Kumar S. Arora S. (2008). Anti-free Radical Activities of Kaempferol Isolated from Acacia Nilotica (L.) Willd. Ex. Del. Toxicol. Vitro 22 (8), 1965–1970. 10.1016/j.tiv.2008.08.007 18805478

[B270] Singh R. K. Chang H. W. Yan D. Lee K. M. Ucmak D. Wong K. (2017). Influence of Diet on the Gut Microbiome and Implications for Human Health. J. Transl Med. 15 (1), 73. 10.1186/s12967-017-1175-y 28388917 PMC5385025

[B271] Singhal T. (2020). A Review of Coronavirus Disease-2019 (COVID-19). Indian J. Pediatr. 87 (4), 281–286. 10.1007/s12098-020-03263-6 32166607 PMC7090728

[B272] Singla R. Mishra A. Joshi R. Jha S. Sharma A. R. Upadhyay S. (2020). Human Animal Interface of SARS-CoV-2 (COVID-19) Transmission: a Critical Appraisal of Scientific Evidence. Vet. Res. Commun. 44 (3-4), 119–130. 10.1007/s11259-020-09781-0 32926266 PMC7487339

[B273] Srinivasan R. Natarajan D. Shivakumar M. S. (2015). Antioxidant Compound Quercetin-3-O-α-L-Rhamnoside(1→6)- β-D-glucose (Rutin) Isolated from Ethyl Acetate Leaf Extracts of Memecylon Edule Roxb (Melastamataceae). Free Rad. Antiox. 5 (1), 35–42. 10.5530/fra.2015.1.6

[B274] Srinivasan R. Natarajan D. Subramaniam Shivakumar M. Nagamurugan N. (2016). Isolation of Fisetin from Elaeagnus Indica Serv. Bull. (Elaeagnaceae) with Antioxidant and Antiproliferative Activity. Fra 6 (2), 145–150. 10.5530/fra.2016.2.3

[B275] Stohs S. J. Sabatka J. J. Obrist J. J. Rosenberg H. (1974). Sapogenins of Yucca Glauca Tissue Cultures. Lloydia 37 (2), 504–505. 10.1016/s0031-9422(00)91240-8 4437310

[B276] Su B. N. Pawlus A. D. Jung H. A. Keller W. J. McLaughlin J. L. Kinghorn A. D. (2005). Chemical Constituents of the Fruits of *Morinda citrifolia* (Noni) and Their Antioxidant Activity. J. Nat. Prod. 68 (4), 592–595. 10.1021/np0495985 15844957

[B277] Su S. Wong G. Shi W. Liu J. Lai A. C. K. Zhou J. (2016). Epidemiology, Genetic Recombination, and Pathogenesis of Coronaviruses. Trends Microbiol. 24 (6), 490–502. 10.1016/j.tim.2016.03.003 27012512 PMC7125511

[B278] Sugita-Konishi Y. Hara-Kudo Y. Amano F. Okubo T. Aoi N. Iwaki M. (1999). Epigallocatechin Gallate and Gallocatechin Gallate in green tea Catechins Inhibit Extracellular Release of Vero Toxin from Enterohemorrhagic *Escherichia coli* O157:H7. Biochim. Biophys. Acta 1472 (1-2), 42–50. 10.1016/s0304-4165(99)00102-6 10572924

[B279] Sundaram R L. L. Sali V. K. Vasanthi H. R. (2018). Protective Effect of Rutin Isolated from Spermococe Hispida against Cobalt Chloride-Induced Hypoxic Injury in H9c2 Cells by Inhibiting Oxidative Stress and Inducing Apoptosis. Phytomedicine 51, 196–204. 10.1016/j.phymed.2018.09.229 30466617

[B280] Syah Y. M. Ghisalberti E. L. (2010). Phenolic Derivatives with an Irregular Sesquiterpenyl Side Chain from Macaranga Pruinosa. Nat. Prod. Commun. 5 (2), 219–222. 10.1177/1934578x1000500209 20334130

[B281] Tachikawa E. Takahashi M. Kashimoto T. (2000). Effects of Extract and Ingredients Isolated from Magnolia Obovata Thunberg on Catecholamine Secretion from Bovine Adrenal Chromaffin Cells. Biochem. Pharmacol. 60 (3), 433–440. 10.1016/s0006-2952(00)00343-9 10856439

[B282] Tahara S. Ingham J. L. Nakahara S. Mizutani J. Harborne J. B. (1984). Fungitoxic Dihydrofuranoisoflavones and Related Compounds in white Lupin, Lupinus Albus. Phytochemistry 23 (9), 1889–1900. 10.1016/s0031-9422(00)84936-5

[B283] Tahir ul Qamar M. Alqahtani S. M. Alamri M. A. Chen L. L. (2020). Structural Basis of SARS-CoV-2 3CLpro and Anti-COVID-19 Drug Discovery from Medicinal Plants. J. Pharm. Anal. 10 (4), 313–319. 10.1016/j.jpha.2020.03.009 32296570 PMC7156227

[B284] Takeda Y. Murata T. Jamsransuren D. Suganuma K. Kazami Y. Batkhuu J. (2020). Saxifraga Spinulosa-Derived Components Rapidly Inactivate Multiple Viruses Including SARS-CoV-2. Viruses 12 (7), 699. 10.3390/v12070699 PMC7411974 32605306

[B285] Tang W. H. Kitai T. Hazen S. L. (2017). Gut Microbiota in Cardiovascular Health and Disease. Circ. Res. 120 (7), 1183–1196. 10.1161/circresaha.117.309715 28360349 PMC5390330

[B286] Tao L. T. Huang T. L. Zheng D. W. Zou X. (2020a). Case of Professor Xu ZOU's Acupuncture Technique for "benefiting Kidney and Strengthening Anti-pathogenic Qi" in Promoting the Absorption of COVID-19. World J. Acupunct Moxibustion 30 (3), 167–170. 10.1016/j.wjam.2020.07.008 32837109 PMC7377728

[B287] Tao Q. Du J. Li X. Zeng J. Tan B. Xu J. (2020b). Network Pharmacology and Molecular Docking Analysis on Molecular Targets and Mechanisms of Huashi Baidu Formula in the Treatment of COVID-19. Drug Dev. Ind. Pharm. 46 (8), 1345–1353. 10.1080/03639045.2020.1788070 32643448 PMC7441778

[B288] Thanh Tam N. Thien D. D. Sung T. V. Thi Hoang Anh N. Thuy T. T. Trung K. H. (2016). Evaluation of Ursolic Acid as the Main Component Isolated from Catharanthus Roseus against Hyperglycemia. Ilns 50, 7–17. 10.18052/www.scipress.com/ILNS.50.7

[B290] Tran K. Cimon K. Severn M. Pessoa-Silva C. L. Conly J. (2012). Aerosol Generating Procedures and Risk of Transmission of Acute Respiratory Infections to Healthcare Workers: a Systematic Review. PLoS One 7 (4), e35797. 10.1371/journal.pone.0035797 22563403 PMC3338532

[B291] van der Lelie D. Taghavi S. Cristea I. M. (2020). COVID-19 and the Gut Microbiome: More Than a Gut Feeling. mSystems 5 (4), e00453. 10.1128/mSystems.00453-20 32694127 PMC7566280

[B292] van Doremalen N. Bushmaker T. Morris D. H. Holbrook M. G. Gamble A. Williamson B. N. (2020). Aerosol and Surface Stability of SARS-CoV-2 as Compared with SARS-CoV-1. N. Engl. J. Med. 382 (16), 1564–1567. 10.1056/NEJMc2004973 32182409 PMC7121658

[B293] Vandeputte D. Falony G. Vieira-Silva S. Tito R. Y. Joossens M. Raes J. (2016). Stool Consistency Is Strongly Associated with Gut Microbiota Richness and Composition, Enterotypes and Bacterial Growth Rates. Gut 65 (1), 57–62. 10.1136/gutjnl-2015-309618 26069274 PMC4717365

[B294] Veeramani C. Alsaif M. A. Al-Numair K. S. (2018). Herbacetin, a Flaxseed Flavonoid, Ameliorates High Percent Dietary Fat Induced Insulin Resistance and Lipid Accumulation through the Regulation of Hepatic Lipid Metabolizing and Lipid-Regulating Enzymes. Chem. Biol. Interact 288, 49–56. 10.1016/j.cbi.2018.04.009 29653099

[B295] Vijayakumar B. G. Ramesh D. Joji A. Jayachandra Prakasan J. Kannan T. (2020). In Silico pharmacokinetic and Molecular Docking Studies of Natural Flavonoids and Synthetic Indole Chalcones against Essential Proteins of SARS-CoV-2. Eur. J. Pharmacol. 886, 173448. 10.1016/j.ejphar.2020.173448 32768503 PMC7406432

[B296] Vivek-Ananth R. P. Rana A. Rajan N. Biswal H. S. Samal A. (2020). In Silico Identification of Potential Natural Product Inhibitors of Human Proteases Key to SARS-CoV-2 Infection. Molecules 25 (17), 3822. 10.3390/molecules25173822 PMC7504347 32842606

[B297] Waage S. K. Hedin P. A. Grimley E. (1984). A Biologically-Active Procyanidin from Machaerium Floribundum. Phytochemistry 23 (12), 2785–2787. 10.1016/0031-9422(84)83016-2

[B298] Wang H. Guo T. Yang Y. Yu L. Pan X. Li Y. (2019). Lycorine Derivative LY-55 Inhibits EV71 and CVA16 Replication through Downregulating Autophagy. Front Cel Infect Microbiol 9, 277. 10.3389/fcimb.2019.00277 PMC6692562 31448243

[B299] Wang H. Nair M. G. Strasburg G. M. Chang Y. C. Booren A. M. Gray J. I. (1999). Antioxidant and Antiinflammatory Activities of Anthocyanins and Their Aglycon, Cyanidin, from Tart Cherries. J. Nat. Prod. 62 (2), 294–296. 10.1021/np980501m 10075763

[B300] Wang J. Zhao S. Liu M. Zhao Z. Xu Y. Wang P. (2020a). ACE2 Expression by Colonic Epithelial Cells Is Associated with Viral Infection, Immunity and Energy Metabolism. Immun. Energ. Metab. 15 (11), e0241955. 10.1101/2020.02.05.20020545 PMC12891528 41554833

[B301] Wang L. Wang X. Yuan X. Zhao B. (2011). Simultaneous Analysis of Diosgenin and Sarsasapogenin in Asparagus Officinalis Byproduct by Thin-Layer Chromatography. Phytochem. Anal. 22 (1), 14–17. 10.1002/pca.1244 20799270

[B302] Wang S. J. Tong Y. Lu S. Yang R. Liao X. Xu Y. F. (2010). Anti-inflammatory activity of myricetin isolated from Myrica rubra Sieb. et Zucc. leaves. Planta Med. 76 (14), 1492–1496. 10.1055/s-0030-1249780 20383816

[B303] Wang W. Xu Y. Gao R. Lu R. Han K. Wu G. (2020b). Detection of SARS-CoV-2 in Different Types of Clinical Specimens. Jama. 10.1001/jama.2020.3786 PMC7066521 32159775

[B304] Wei J. Zhao J. Han M. Meng F. Zhou J. (2020a). SARS-CoV-2 Infection in Immunocompromised Patients: Humoral versus Cell-Mediated Immunity. J. Immunother. Cancer 8 (2), e000862. 10.1136/jitc-2020-000862 32727811 PMC7431770

[B305] Wei T. Z. Wang H. Wu X. Q. Lu Y. Guan S. H. Dong F. Q. (2020b). In Silico Screening of Potential Spike Glycoprotein Inhibitors of SARS-CoV-2 with Drug Repurposing Strategy. Chin. J. Integr. Med. 26 (9), 663–669. 10.1007/s11655-020-3427-6 32740825 PMC7395204

[B306] Worldometer (2020). COVID 19 Coronavirus Pandemic. [Online]. Available: https://www.worldometers.info/coronavirus/(Accessed 11 22, 2020 2020).

[B307] Wu C. Y. Jan J. T. Ma S. H. Kuo C. J. Juan H. F. Cheng Y. S. (2004). Small Molecules Targeting Severe Acute Respiratory Syndrome Human Coronavirus. Proc. Natl. Acad. Sci. U S A. 101 (27), 10012–10017. 10.1073/pnas.0403596101 15226499 PMC454157

[B308] Wu X. Liu Y. Sheng W. Sun J. Qin G. (2007). Chemical Constituents of Isatis Indigotica. Planta Med. 63 (01), 55–57. 10.1055/s-2006-957604 17252328

[B309] Wu X. Zhao Y. Haytowitz D. B. Chen P. Pehrsson P. R. (2019). Effects of Domestic Cooking on Flavonoids in Broccoli and Calculation of Retention Factors. Heliyon 5 (3), e01310. 10.1016/j.heliyon.2019.e01310 30899833 PMC6407093

[B310] Xiao F. Tang M. Zheng X. Liu Y. Li X. Shan H. (2020). Evidence for Gastrointestinal Infection of SARS-CoV-2. Gastroenterology 158 (6), 1831. 10.1053/j.gastro.2020.02.055 32142773 PMC7130181

[B311] Xing Y. H. Ni W. Wu Q. Li W. J. Li G. J. Wang W. D. (2020). Prolonged Viral Shedding in Feces of Pediatric Patients with Coronavirus Disease 2019. J. Microbiol. Immunol. Infect. 53 (3), 473–480. 10.1016/j.jmii.2020.03.021 32276848 PMC7141453

[B312] Yan J. Tong S. Chu J. Sheng L. Chen G. (2004). Preparative Isolation and Purification of Syringin and Edgeworoside C from Edgeworthia Chrysantha Lindl by High-Speed Counter-current Chromatography. J. Chromatogr. A. 1043 (2), 329–332. 10.1016/j.chroma.2004.05.087 15330108

[B313] Yan R. Zhang Y. Li Y. Xia L. Guo Y. Zhou Q. (2020). Structural Basis for the Recognition of SARS-CoV-2 by Full-Length Human ACE2. Science 367 (6485), 1444–1448. 10.1126/science.abb2762 32132184 PMC7164635

[B314] Yang C. W. Lee Y. Z. Kang I. J. Barnard D. L. Jan J. T. Lin D. (2010). Identification of Phenanthroindolizines and Phenanthroquinolizidines as Novel Potent Anti-coronaviral Agents for Porcine Enteropathogenic Coronavirus Transmissible Gastroenteritis Virus and Human Severe Acute Respiratory Syndrome Coronavirus. Antivir. Res 88 (2), 160–168. 10.1016/j.antiviral.2010.08.009 20727913 PMC7114283

[B315] Yang D. S. Peng W. B. Yang Y. P. Liu K. C. Li X. L. Xiao W. L. (2015). Cytotoxic Prenylated Flavonoids from Macaranga Indica. Fitoterapia 103, 187–191. 10.1016/j.fitote.2015.04.002 25861749

[B316] Yang J. Petitjean S. J. L. Koehler M. Zhang Q. Dumitru A. C. Chen W. (2020a). Molecular Interaction and Inhibition of SARS-CoV-2 Binding to the ACE2 Receptor. Nat. Commun. 11 (1), 4541. 10.1038/s41467-020-18319-6 32917884 PMC7486399

[B317] Yang M.-H. Patel A. V. Blunden G. Turner C. H. O'Neill M. J. Lewist J. A. (1993). Crabbine, an Aporphine Alkaloid from Corydalis Lutea. Phytochemistry 33 (4), 943–945. 10.1016/0031-9422(93)85313-g

[B318] Yang Y. Peng F. Wang R. Yange K. Guan K. Jiang T. (2020b). The Deadly Coronaviruses: The 2003 SARS Pandemic and the 2020 Novel Coronavirus Epidemic in China. J. Autoimmun. 109, 102434. 10.1016/j.jaut.2020.102434 32143990 PMC7126544

[B319] Yepes-Pérez A. F. Herrera-Calderon O. Sánchez-Aparicio J. E. Tiessler-Sala L. Maréchal J. D. Cardona-G W. (2020). Investigating Potential Inhibitory Effect of Uncaria Tomentosa (Cat's Claw) against the Main Protease 3CLpro of SARS-CoV-2 by Molecular Modeling. Evid. Based Complement. Alternat Med. 2020, 4932572. 10.1155/2020/4932572 33029165 PMC7532411

[B320] Yi L. Li Z. Yuan K. Qu X. Chen J. Wang G. (2004). Small Molecules Blocking the Entry of Severe Acute Respiratory Syndrome Coronavirus into Host Cells. J. Virol. 78 (20), 11334–11339. 10.1128/JVI.78.20.11334-11339.2004 15452254 PMC521800

[B321] Yin M. Zhang P. Yu F. Zhang Z. Cai Q. Lu W. (2017). Grape Seed Procyanidin B2 Ameliorates Hepatic Lipid Metabolism Disorders in Db/db Mice. Mol. Med. Rep. 16 (3), 2844–2850. 10.3892/mmr.2017.6900 28677803

[B322] Yoo Y. M. Nam J. H. Kim M. Y. Choi J. Park H. J. (2008). Pectolinarin and Pectolinarigenin of Cirsium Setidens Prevent the Hepatic Injury in Rats Caused by D-Galactosamine via an Antioxidant Mechanism. Biol. Pharm. Bull. 31 (4), 760–764. 10.1248/bpb.31.760 18379079

[B323] Yu F. Harada H. Yamasaki K. Okamoto S. Hirase S. Tanaka Y. (2008). Isolation and Functional Characterization of a Beta-Eudesmol Synthase, a New Sesquiterpene Synthase from Zingiber Zerumbet Smith. FEBS Lett. 582 (5), 565–572. 10.1016/j.febslet.2008.01.020 18242187

[B324] Yu R. Chen L. Lan R. Shen R. Li P. (2020). Computational Screening of Antagonists against the SARS-CoV-2 (COVID-19) Coronavirus by Molecular Docking. Int. J. Antimicrob. Agents 56 (2), 106012. 10.1016/j.ijantimicag.2020.106012 32389723 PMC7205718

[B325] Zahra Ahmadian Dehaghani D. Gholamreza Asghari A. Masoud Sadeghi Dinani D. (2017). Isolation and Identification of Nicotiflorin and Narcissin from the Aerial Parts of Peucedanum Aucheri Boiss. JAST-A 7 (1). 10.17265/2161-6256/2017.01.007

[B326] Zandi K. Musall K. Oo A. Cao D. Liang B. Hassandarvish P. (2021). Baicalein and Baicalin Inhibit SARS-CoV-2 RNA-Dependent-RNA Polymerase. Microorganisms 9 (5), 893. 10.3390/microorganisms9050893 33921971 PMC8143456

[B327] Zhang Y. Dong H. Wang M. Zhang J. (2016). Quercetin Isolated from *Toona sinensis* Leaves Attenuates Hyperglycemia and Protects Hepatocytes in High-Carbohydrate/High-Fat Diet and Alloxan Induced Experimental Diabetic Mice. J. Diabetes Res. 2016, 8492780. 10.1155/2016/8492780 27975068 PMC5126429

[B328] Zhang Y. N. Zhang Q. Y. Li X. D. Xiong J. Xiao S. Q. Wang Z. (2020a). Gemcitabine, Lycorine and Oxysophoridine Inhibit Novel Coronavirus (SARS-CoV-2) in Cell Culture. Emerg. Microbes Infect. 9 (1), 1170–1173. 10.1080/22221751.2020.1772676 32432977 PMC7448857

[B329] Zhang Z. J. Wu W. Y. Hou J. J. Zhang L. L. Li F. F. Gao L. (2020b). Active Constituents and Mechanisms of Respiratory Detox Shot, a Traditional Chinese Medicine Prescription, for COVID-19 Control and Prevention: Network-Molecular Docking-LC-MSE Analysis. J. Integr. Med. 18 (3), 229–241. 10.1016/j.joim.2020.03.004 32307268 PMC7195604

[B330] Zhao J. Zhang S. You S. Liu T. Xu F. Ji T. (2017a). Hepatoprotective Effects of Nicotiflorin from Nymphaea candida against Concanavalin A-Induced and D-Galactosamine-Induced Liver Injury in Mice. Int. J. Mol. Sci. 18 (3), 587. 10.3390/ijms18030587 PMC5372603 28282879

[B331] Zhao X. Cui Q. Fu Q. Song X. Jia R. Yang Y. (2017b). Antiviral Properties of Resveratrol against Pseudorabies Virus Are Associated with the Inhibition of IκB Kinase Activation. Sci. Rep. 7 (1), 8782. 10.1038/s41598-017-09365-0 28821840 PMC5562710

[B332] Zheng J. (2020). SARS-CoV-2: an Emerging Coronavirus that Causes a Global Threat. Int. J. Biol. Sci. 16 (10), 1678–1685. 10.7150/ijbs.45053 32226285 PMC7098030

[B333] Zheng S. Baak J. P. Li S. Xiao W. Ren H. Yang H. (2020). Network Pharmacology Analysis of the Therapeutic Mechanisms of the Traditional Chinese Herbal Formula Lian Hua Qing Wen in Corona Virus Disease 2019 (COVID-19), Gives Fundamental Support to the Clinical Use of LHQW. Phytomedicine 79, 153336. 10.1016/j.phymed.2020.153336 32949888 PMC7474845

[B334] Zheng Z.-P. Cheng K.-W. Chao J. Wu J. Wang M. (2008). Tyrosinase Inhibitors from Paper mulberry (Broussonetia Papyrifera). Food Chem. 106 (2), 529–535. 10.1016/j.foodchem.2007.06.037

[B335] Zhou J. Gupta K. Aggarwal S. Aneja R. Chandra R. Panda D. (2003). Brominated Derivatives of Noscapine Are Potent Microtubule-Interfering Agents that Perturb Mitosis and Inhibit Cell Proliferation. Mol. Pharmacol. 63 (4), 799–807. 10.1124/mol.63.4.799 12644580

[B336] Zhou Y. Liu X. Yang Z. (2019). Characterization of Terpene Synthase from Tea Green Leafhopper Being Involved in Formation of Geraniol in Tea (Camellia Sinensis) Leaves and Potential Effect of Geraniol on Insect-Derived Endobacteria. Biomolecules 9 (12), 808. 10.3390/biom9120808 PMC6995508 31801241

[B337] Zou J. C. Huang L. (1985). Minor Constituents of Qing Dai, a Traditional Chinese Medicine. I. Isolation, Structural Determination and Synthesis of Tryptanthrin and Qingdainone. Yao Xue Xue Bao 20 (1), 45–51. 3839621

[B338] Zuo T. Liu Q. Zhang F. Lui G. C.-Y. Tso E. Y. Yeoh Y. K. (2020). Depicting SARS-CoV-2 Faecal Viral Activity in Association with Gut Microbiota Composition in Patients with COVID-19. Gut, gutjnl–2020. 10.1136/gutjnl-2020-322294 PMC7385744 32690600

