# Supplementary figures and images for: Natural Products for the Prevention and Control of the COVID-19 Pandemic: Sustainable Bioresources
Source: Front Pharmacol. 2021 Dec 1;12:758159. doi: 10.3389/fphar.2021.758159 (PMC8671886; doi:10.3389/fphar.2021.758159)

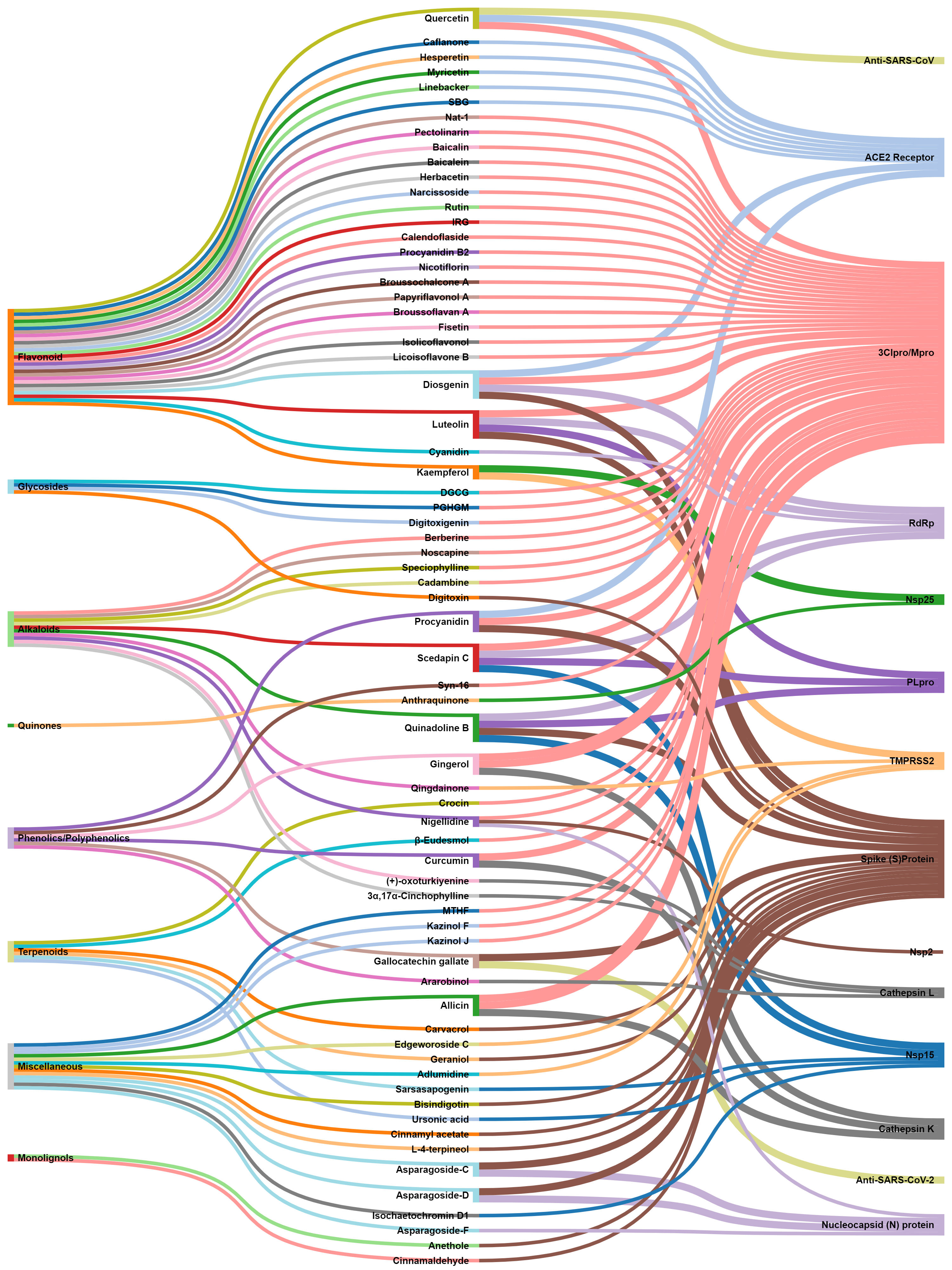

Supplement: Supplementary file 1 [file Image2.PNG]

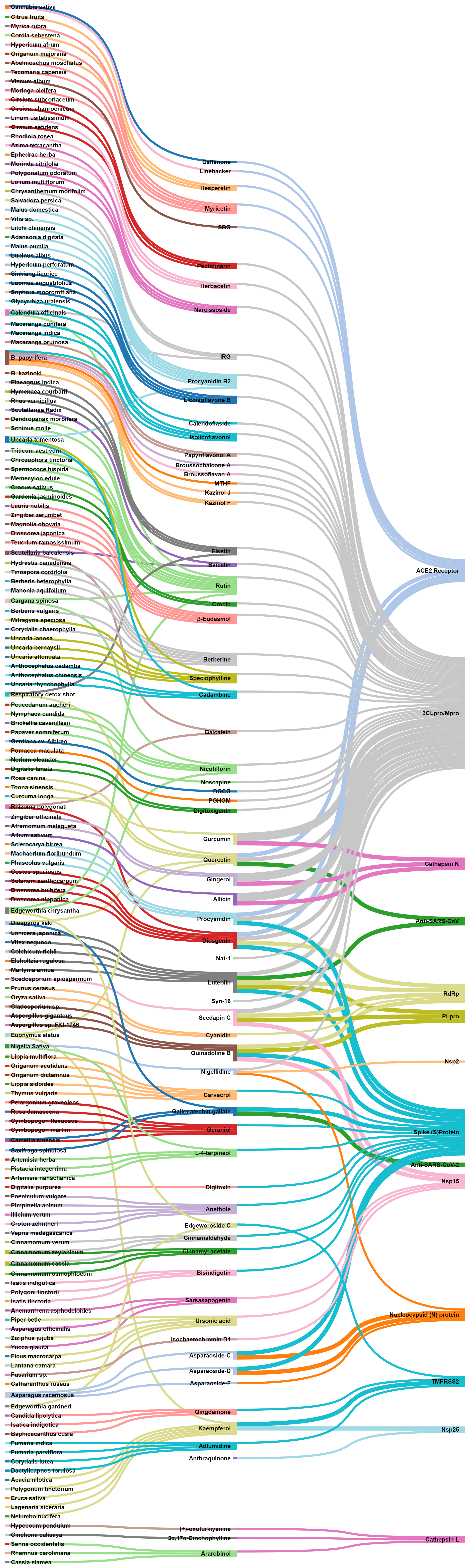

Supplement: Supplementary file 2 [file Image1.PNG]

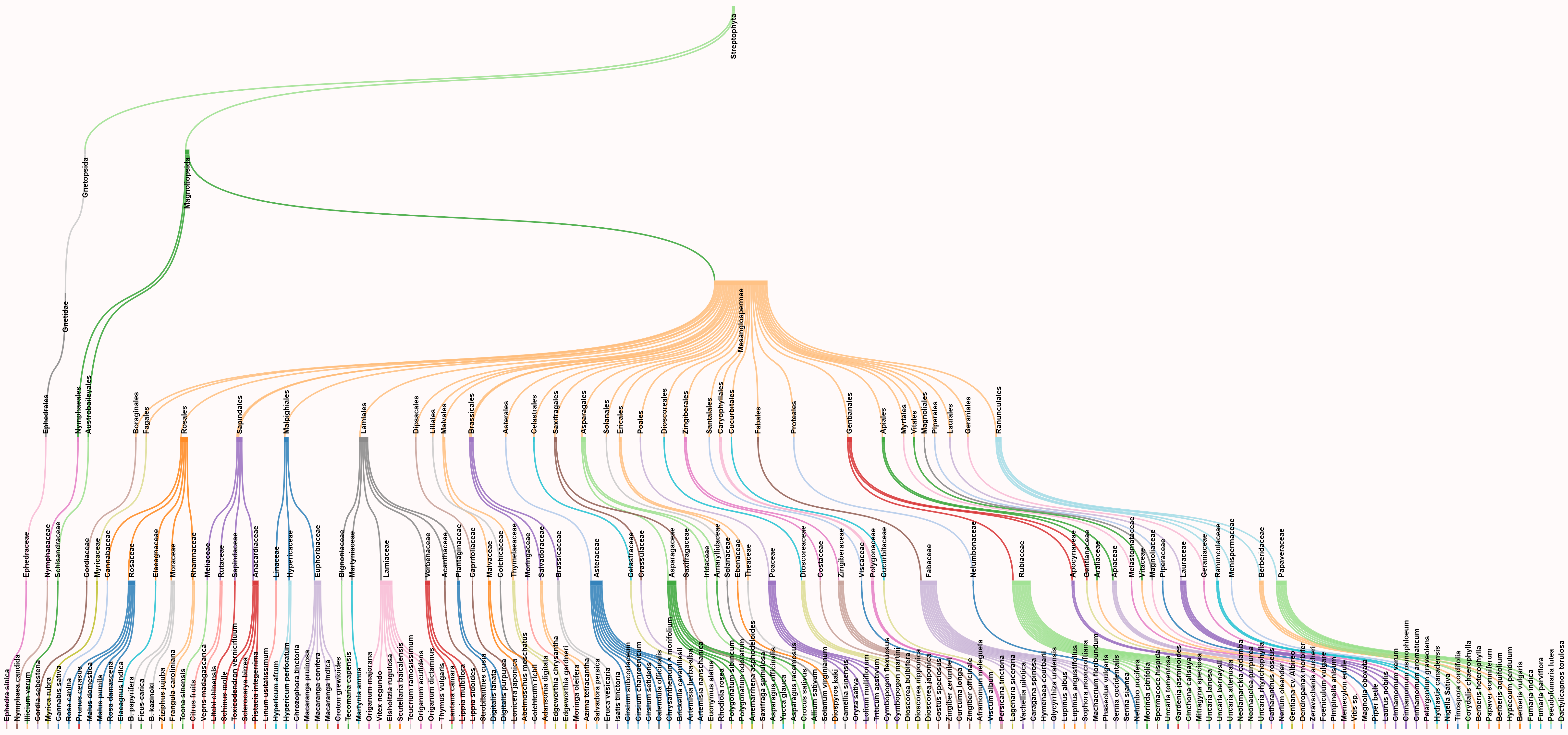

Supplement: Supplementary file 3 [file Image3.PNG]
